# Tensor Decomposition Reveals Concurrent Evolutionary Convergences and Divergences and Correlations with Structural Motifs in Ribosomal RNA

**DOI:** 10.1371/journal.pone.0018768

**Published:** 2011-04-29

**Authors:** Chaitanya Muralidhara, Andrew M. Gross, Robin R. Gutell, Orly Alter

**Affiliations:** 1 Institute for Cellular and Molecular Biology, University of Texas at Austin, Austin, Texas, United States of America; 2 Bioinformatics and Systems Biology Program, University of California at San Diego, San Diego, California, United States of America; 3 Section of Integrative Biology and Center for Computational Biology and Bioinformatics, University of Texas at Austin, Austin, Texas, United States of America; 4 Scientific Computing and Imaging (SCI) Institute and Departments of Bioengineering and Human Genetics, University of Utah, Salt Lake City, Utah, United States of America; Université Paris Sud, France

## Abstract

Evolutionary relationships among organisms are commonly described by using a hierarchy derived from comparisons of ribosomal RNA (rRNA) sequences. We propose that even on the level of a single rRNA molecule, an organism's evolution is composed of multiple pathways due to concurrent forces that act independently upon different rRNA degrees of freedom. Relationships among organisms are then compositions of coexisting pathway-dependent similarities and dissimilarities, which cannot be described by a single hierarchy. We computationally test this hypothesis in comparative analyses of 16S and 23S rRNA sequence alignments by using a tensor decomposition, i.e., a framework for modeling composite data. Each alignment is encoded in a cuboid, i.e., a third-order tensor, where nucleotides, positions and organisms, each represent a degree of freedom. A tensor mode-1 higher-order singular value decomposition (HOSVD) is formulated such that it separates each cuboid into combinations of patterns of nucleotide frequency variation across organisms and positions, i.e., “eigenpositions” and corresponding nucleotide-specific segments of “eigenorganisms,” respectively, independent of a-priori knowledge of the taxonomic groups or rRNA structures. We find, in support of our hypothesis that, first, the significant eigenpositions reveal multiple similarities and dissimilarities among the taxonomic groups. Second, the corresponding eigenorganisms identify insertions or deletions of nucleotides exclusively conserved within the corresponding groups, that map out entire substructures and are enriched in adenosines, unpaired in the rRNA secondary structure, that participate in tertiary structure interactions. This demonstrates that structural motifs involved in rRNA folding and function are evolutionary degrees of freedom. Third, two previously unknown coexisting subgenic relationships between Microsporidia and Archaea are revealed in both the 16S and 23S rRNA alignments, a convergence and a divergence, conferred by insertions and deletions of these motifs, which cannot be described by a single hierarchy. This shows that mode-1 HOSVD modeling of rRNA alignments might be used to computationally predict evolutionary mechanisms.

## Introduction

The ribosomal RNA (rRNA) is an essential component of the ribosome, the cellular organelle that associates the cell's genotype with its phenotype by catalyzing protein synthesis in all known organisms, and therefore also underlies cellular evolution. RNAs are thought to be among the most primordial macromolecules. This is because an RNA template, similar to a DNA template, can be used to synthesize DNA and RNA, while RNA, similar to proteins, can form three-dimensional structures and catalyze reactions. It was suggested, therefore, that rRNA sequences and structures, that are similar or dissimilar among groups of organisms, are indicative of the relative evolutionary pathways of these organisms [1]–[3]. Advances in sequencing technologies have resulted in an abundance of rRNA sequences from organisms spanning all taxonomic groups. Today, the small subunit ribosomal RNA (16S rRNA) is the gene with the largest number of determined sequences.

Evolutionary relationships among organisms are commonly described by using a hierarchy derived from comparisons of rRNA sequences. Secondary and tertiary structure models of rRNAs are also being derived from comparisons of rRNA sequences by using sequence conservation and covariation analyses, and assuming that sequence positions with similar patterns of variation across multiple organisms are base-paired in the rRNA structure [4]–[7]. The determination of the high-resolution crystal structures of the ribosome [8]–[10] substantiate these structure models, with approximately 97% of the proposed base pairs present in the crystal structures [11].

We propose that even on the level of a single rRNA molecule, an organism's evolution is composed of multiple pathways [12] due to concurrent evolutionary forces that act independently upon different rRNA degrees of freedom [13] of multiple types, i.e., different nucleotides in different positions of the molecule, that might correspond to different structural and functional components of the molecule, and in different groups of organisms. The relationships among organisms are then compositions of coexisting evolutionary pathway-dependent rRNA similarities and dissimilarities, which cannot be described by a single hierarchy.

We computationally test this hypothesis in comparative analyses of 16S and 23S rRNA sequence alignments [14] by using a tensor decomposition, i.e., a mathematical framework for modeling composite data with multiple types of degrees of freedom. Each alignment is encoded in a cuboid, i.e., a third-order tensor, where the nucleotides, positions and organisms, each represent a possible degree of freedom. We note that an encoding that transforms a sequence alignment into a second-order tensor, i.e., a matrix, effectively introduces into the analysis assumptions regarding the relations among the nucleotides in the rRNA structure. Similarly, in the analysis of the tensor unfolded into a matrix, some of the degrees of freedom are lost and much of the information in the alignment might also be lost. A tensor mode-1 higher-order singular value decomposition (HOSVD) [15]–[18] is, therefore, formulated such that it separates each cuboid into combinations of patterns of nucleotide frequency variation across organisms and positions, i.e., “eigenpositions” and corresponding nucleotide-specific segments of “eigenorganisms,” respectively, independent of a-priori knowledge of the taxonomic groups [19] or rRNA structures.

We find, in support of our hypothesis that, first, the significant eigenpositions reveal multiple similarities and dissimilarities among the taxonomic groups, some known and some previously unknown. Second, the corresponding eigenorganisms identify insertions or deletions of nucleotides exclusively conserved within the corresponding groups, that map out entire substructures, some known and some previously unknown, and are enriched in adenosines, unpaired in the rRNA secondary structure, that participate in tertiary structure interactions [20]–[26]. This demonstrates that structural motifs involved in rRNA folding and function are evolutionary degrees of freedom. Third, two previously unknown coexisting subgenic relationships between Microsporidia [27]–[29] and Archaea [30], [31] are revealed in both the 16S and 23S rRNAs, a convergence and a divergence, conferred by insertions and deletions of these motifs, which cannot be described by a single hierarchy.

These analyses show that the mode-1 HOSVD provides a mathematical framework for the modeling of rRNA sequence alignments, independent of a-priori knowledge of the taxonomic groups and their relationships, or the rRNA structures, where the mathematical variables represent biological reality [32]. The significant eigenpositions and corresponding nucleotide-specific segments of eigenorganisms represent multiple subgenic evolutionary relationships of convergence and divergence and correlations with structural motifs, some known and some previously unknown, that are consistent with current biological understanding of the 16S and 23S rRNAs. Mode-1 HOSVD modeling of rRNA sequence alignments, therefore, might be used to computationally predict evolutionary mechanisms, i.e., evolutionary pathways and the underlying structural changes that these pathways are correlated, possibly even coordinated with.

## Methods

**Figure 1:**
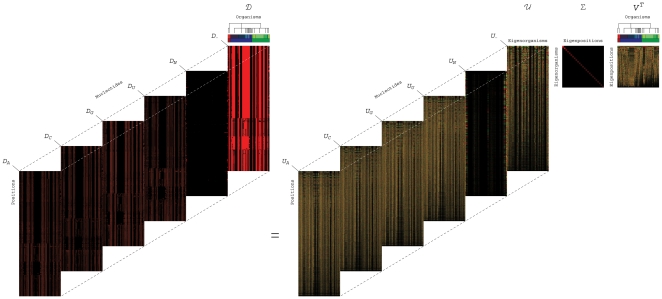
Mode-1 HOSVD of the 16S rRNA sequence alignment. Organisms, positions and sequence elements, each represent a degree of freedom in the alignment encoded in a cuboid (Equation 1). Mode-1 HOSVD (Equation 2) separates the alignment into combinations of “eigenpositions” and nulceotide-specific segments of “eigenorganisms,” i.e., patterns of nucleotide frequency variation across the organisms and positions, with increase (red), no change (black) and decrease in the nucleotide frequency (green) relative to the average frequency across the organisms and positions. It was shown that SVD provides a framework for modeling DNA microarray data [32]: The mathematical variables, significant patterns uncovered in the data, correlate with activities of cellular elements, such as regulators or transcription factors. The mathematical operations simulate experimental observation of the correlations and possibly causal coordination of these activities. Recent experimental results [16] demonstrate that SVD modeling of DNA microarray data can be used to correctly predict previously unknown cellular mechanisms [15], [36]. We now show that mode-1 HOSVD, which is computed by using SVD (Equation 3), provides a framework for modeling rRNA sequence alignments: The mathematical variables, significant patterns of nucleotide frequency variation, represent multiple subgenic evolutionary relationships of convergence and divergence among the organisms, some known and some previously unknown, and correlations with structural motifs. Our mode-1 HOSVD analyses of 16S and 23 rRNA alignments support the hypothesis that even on the level of a single rRNA molecule, an organism's evolution is composed of multiple pathways due to concurrent forces that act independently upon different rRNA degrees of freedom. These analyses demonstrate that entire rRNA substructures and unpaired adenosines, i.e., rRNA structural motifs which are involved in rRNA folding and function, are evolutionary degrees of freedom. These analyses also show that mode-1 HOSVD modeling of rRNA alignments might be used to computationally predict evolutionary mechanisms, i.e., evolutionary pathways and the underlying structural changes that these pathways are correlated, possibly even coordinated with.

To computationally test whether the mathematical nature of evolutionary relationships is that of a hierarchy or a composition, we analyze alignments of 339 16S and 75 23S rRNA sequences from the Comparative RNA Website (CRW) [14], representing all 16S and 23S sequences for which a secondary structure model is available. The organisms corresponding to these sequences are from different National Center for Biotechnology Information (NCBI) Taxonomy Browser groups [19]: The 339 16S organisms include 21 Archaea, 175 Bacteria and 143 Eukarya (Datasets S1, S2, S3); the 75 23S organisms include six Archaea, 57 Bacteria and 12 Eukarya (Datasets S4, S5, S6). The alignments tabulate six sequence elements or “nucleotides,” i.e., A, C, G and U nucleotides, unknown (“N”) and gap (“
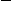
”), across 339 organisms and 3249 sequence positions for the 16S, or 75 organisms and 6636 sequence positions for the 23S, with A, C, G or U nucleotides, but not an unknown or a gap, in at least 1% of the 339 or 75 organisms, respectively. A six-bit binary encoding [33],
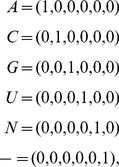
(1)


transforms each alignment matrix into a cuboid, i.e., a third-order tensor, of six “slices,” one slice for each nucleotide, tabulating the frequency of this nucleotide across the organisms and positions, where the nucleotides, positions and organisms, each represent a possible degree of freedom (Figure 1 and Mathematica Notebooks S1, S2, S3, S4). Note that an encoding that transforms a sequence alignment into a second-order tensor, i.e., a matrix, effectively introduces into the analysis assumptions regarding the relations among the nucleotides in the RNA structure. Similarly, in the analysis of the tensor unfolded into a matrix, some of the degrees of freedom are lost and much of the information in the alignment might also be lost.

To comparatively analyze the 16S and 23S rRNA alignments, therefore, we use a tensor mode-1 higher-order singular value decomposition (HOSVD) [15], [16]. We formulate the mode-1 HOSVD such that it transforms each *K*-organisms 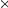 *L* = 6-nucleotides 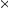 *M*-positions tensor 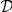 into the reduced and diagonalized *K*-“eigenpositions” 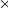 *K*-“eigenorganisms” matrix 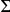, by using the *K*-eigenorganisms 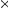 *L* = 6-nucleotides 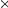 *M*-positions transformation tensor 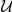 and the *K*-organisms 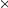 *K*-eigenpositions transformation matrix 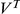,
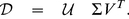
(2)


The mode-1 HOSVD is computed from the singular value decomposition (SVD) [17], [18] of each data tensor unfolded along the *K*-organisms axis such that its nucleotide-specific slices 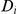 are appended along the organisms axis,
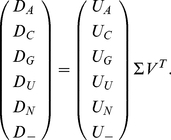
(3)


The transformation tensor 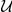 is obtained by stacking the nucleotide-specific slices 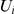 along the organisms axis.

The significance of each eigenposition and the corresponding eigenorganism is defined in terms of the fraction of the overall information that these orthogonal patterns of nucleotide frequency variation across the *K*-organisms and *L* = 6-nucleotides 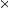 *M*-positions, respectively, capture in the data tensor and is proportional to the corresponding singular value that is listed in 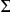. These singular values are ordered in decreasing order, such that the patterns are ordered in decreasing order of their relative significance. We find that the seven and five most significant eigenpositions and corresponding eigenorganisms uncovered in the 16S and 23S alignments, respectively, capture 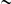
88% and 87% of the nucleotide frequency information in these alignments (Figures S1 and S2 in Appendix S1). In both alignments, the most significant eigenposition is approximately invariant across the organisms and correlates with the average frequency of all nucleotides across the positions [34], with the correlation 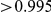. The correlation of each nucleotide-specific segment of the most significant eigenorganism with the average frequency of this nucleotide across the positions is 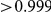.

We interpret the remaining eigenpositions and the nucleotide-specific segments of the corresponding eigenorganisms as patterns of nucleotide frequency variation relative to these averages. We find that the patterns uncovered in the 16S and 23S are qualitatively similar. Note also that these patterns are robust to variations in the selection of organisms for the 16S and 23S alignments, and therefore also to variations in the rRNA positions that each alignment spans.

## Results

**Figure 2:**
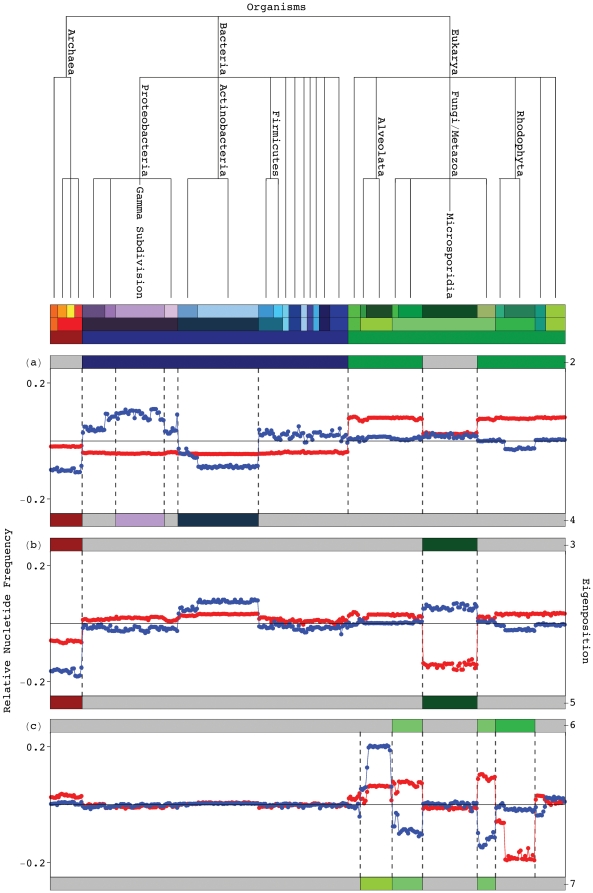
Significant 16S eigenpositions. Line-joined graphs of the second through seventh 16S eigenpositions, i.e., patterns of nucleotide frequency across the organisms, and their correlation with the taxonomic groups in the 16S alignment, classified according to the top six hierarchical levels of the NCBI Taxonomy Browser [19] (Figure S1 in Appendix S1). (*a*) The second most significant eigenposition (red) differentiates the Eukarya excluding the Microsporidia from the Bacteria, as indicated by the color bar (Table 1). The fourth (blue) distinguishes between the Gamma Proteobacteria and the Actinobacteria and Archaea. (*b*) The third (red) and fifth (blue) eigenpositions describe similarities and dissimilarities among the Archaea and Microsporidia, respectively. (*c*) The sixth (red) and seventh (blue) eigenpositions differentiate the Fungi/Metazoa excluding the Microsporidia from the Rhodophyta and the Alveolata, respectively.

We find, in support of our hypothesis, that, first, the significant eigenpositions uncovered in both the 16S and 23S alignments, starting with the second most significant eigenposition in each alignment (Figure 2, and Figure S3 in Appendix S1) reveal multiple similarities and dissimilarities among the taxonomic groups, some known and some previously unknown. To biologically interpret the mathematical patterns of nucleotide frequency variation across the organisms, i.e., the eigenpositions, we correlate and anticorrelate each eigenposition with increased relative nucleotide frequency across a taxonomic group according to the NCBI Taxonomy Browser annotations [19] of the two groups of *k* organisms each, with largest and smallest levels of nucleotide frequency in this eigenposition among all *K* organisms, respectively. The *P*-value of a given association is calculated assuming hypergeometric probability distribution of the *J* annotations among the *K* organisms, and of the subset of 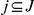 annotations among the subset of *k* organisms, as described [35], 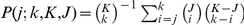 (Tables 1 and 2).

**Table 1:** Enrichment of the significant 16S eigenpositions in taxonomic groups.

Eigenposition	Correlated				Anticorrelated			
	Taxonomic Group	*j*	*J*	*P*-value	Taxonomic Group	*j*	*J*	*P*-value
2	Eukarya	75	107	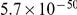	Bacteria	75	175	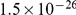
	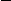 Microsporidia							
3					Archaea	57	57	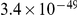
					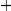 Microsporidia			
4	Gamma	32	32	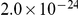	Actinobacteria	72	74	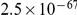
	Proteobacteria				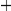 Archaea			
5	Microsporidia	29	36	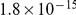	Archaea	21	21	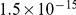
6	Fungi/Metazoa	32	32	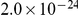	Rhodophyta	26	26	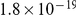
	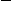 Microsporidia							
7	Alveolata	21	21	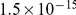	Fungi/Metazoa	32	32	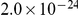
					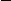 Microsporidia			

Probabilistic significance of the enrichment of the *k* = 75 organisms, with largest relative nucleotide frequency increase or decrease in each of the significant eigenpositions, in the respective taxonomic groups. The *P*-value of each enrichment is calculated as described [35], assuming hypergeometric probability distribution of the *J* annotations among the *K* = 339 organisms, and of the subset of 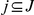 annotations among the subset of *k* = 75 organisms.

**Table 2:** Enrichment of the significant 23S eigenpositions in taxonomic groups.

Eigenposition	Correlated				Anticorrelated			
	Taxonomic Group	*j*	*J*	*P*-value	Taxonomic Group	*j*	*J*	*P*-value
2	Eukarya	8	8	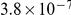	Bacteria	15	57	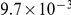
	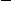 Microsporidia							
3					Archaea	10	10	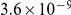
					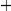 Microsporidia			
4	Proteobacteria	15	23	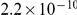	Firmicutes	12	13	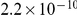
5	Microsporidia	4	4	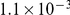	Archaea	6	6	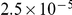

Probabilistic significance of the enrichment of the *k* = 15 organisms, with largest relative nucleotide frequency increase or decrease in each of the significant eigenpositions, in the respective taxonomic groups. The *P*-value of each enrichment is calculated as described [35], assuming hypergeometric probability distribution of the *J* annotations among the *K* = 75 organisms, and of the subset of 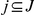 annotations among the subset of *k* = 15 organisms.

In both alignments, the second most significant eigenposition captures the dissimilar between the Eukarya excluding the Microsporidia and the Bacteria. These patterns of relative nucleotide frequency across the organisms correlate with increased frequency across the Eukarya excluding the Microsporidia and decreased frequency across the Bacteria, with both *P*-values 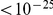 and 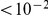 in the 16S and 23S alignments, respectively. The fourth 16S eigenposition correlates with increased nucleotide frequency across the Gamma Proteobacteria and decreased frequency across the Actinobacteria and Archaea, with both *P*-values 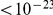. Note that the Gamma Proteobacteria and the Actinobacteria are the two largest bacterial groups in this alignment. The fourth 23S eigenposition captures the dissimilar between the Proteobacteria and the Firmicutes, the two largest bacterial groups in this alignment. In both alignments, the third and fifth eigenpositions capture the similar and dissimilar between the Archaea and Microsporidia, respectively. In the 16S alignment, the sixth and seventh eigenpositions identify dissimilarities among the Fungi/Metazoa excluding the Microsporidia and the Rhodophyta and separately the Alveolata, respectively.

Second, we find that, consistent with the eigenpositions, the corresponding 16S and 23S eigenorganisms identify positions of nucleotides that are approximately conserved within the respective taxonomic groups, but not among them. These positions are significantly enriched in conserved sequence gaps which map out entire substructures inserted or deleted in the 16S and 23S rRNAs of one taxonomic group relative to another as well as adenosines, unpaired in the rRNA secondary structure, most of which participate in tertiary structure interactions and map to the same substructures. To biologically interpret the mathematical patterns of nucleotide-specific frequency variation across the positions, i.e., the eigenorganisms, we consider the *m* positions with largest increase or decrease in the relative nucleotide frequency in each nucleotide-specific segment of each eigenorganism (Tables 3 and 4). These positions exhibit the frequency variations across the organisms that are most correlated or anticorrelated, respectively, with the corresponding eigenposition. We calculate the *P*-value of the enrichment of these positions in sequence and structure motifs conserved across the corresponding taxonomic groups by assuming hypergeometric probability distribution of the *N* conserved motifs among the *M* positions, and of the subset of 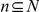 motifs among the subset of *m* positions, as described [35], 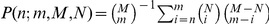.

**Table 3:** Enrichment of the significant 16S eigenorganisms sequence and structure motifs exclusively conserved in taxonomic groups.

Eigenorganism	NucleotideSegment	StructureMotif	Correlated				Anticorrelated			
			Taxonomic Group	*n*	*N*	*P*-value	Taxonomic Group	*n*	*N*	*P*-value
2	A	Unpaired A	Eukarya 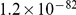 Microsporidia	48	66	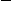	Bacteria	50	50	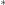
	Gap	Gap		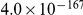 124	211	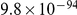		57	58	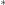
		Unpaired A	Bacteria	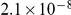 13	50	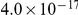				
3	C	Helix					Archaea 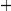 Microsporidia	76	1148	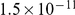
	G							68	1148	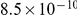
	U							65	1148	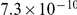
	Gap	Gap						6	6	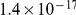
4	A	Unpaired A	GammaProteobacteria	11	11	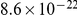				
	Gap	Helix	Actinobacteria 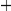 Archaea	34	153	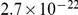				
5	A	Unpaired A					Archaea	14	14	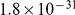
	G	Helix						84	933	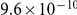
	C		Microsporidia	58	947	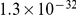		85	933	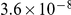
	U			55	947	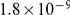		57	933	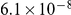
	Gap	Unpaired A	Archaea	7	14	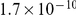				
6	A		Fungi/Metazoa 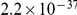 Microsporidia	9	16	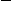	Rhodophyta	25	27	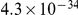
7			Alveolata	25	31	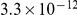	Fungi/Metazoa 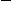 Microsporidia	10	16	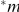

Probabilistic significance of the enrichment of the *m* = 100 positions with largest frequency increase or decrease in each of the nulceotide-specific segments of the significant eigenorganisms (except for the gap segment of the second eigenorganism, where the largest nucleotide frequency increase is shared by 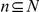 = 124 positions) in sequence and structure motifs exclusively conserved in the corresponding taxonomic groups. The *P*-value of each enrichment is calculated assuming, for each nucleotide, hypergeometric probability distribution of the *N* conserved motifs among the *M* = 3249 positions, and of the subset of 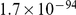 motifs among the subset of *m* positions. Exclusive sequence gap conservation is defined as conservation of gaps within at least 80% of the organisms of the corresponding taxonomic group but in less than 20% of the remaining organisms. Exclusive unpaired A nucleotide conservation is defined as conservation of an adenosine within at least 80% of the organisms of the group but in less than 20% of the remaining organisms, together with greater frequency of unpaired nucleotides within the group rather than among the remaining organisms. Helix conservation is defined as conservation of base-paired nucleotides within at least 60% of the organisms of the group.

**Table 4:** Enrichment of the significant 23S eigenorganisms in sequence and structure motifs exclusively conserved in taxonomic groups.

Eigenorganism	NucleotideSegment	StructureMotif	Correlated				Anticorrelated			
			Taxonomic Group	*n*	*N*	*P*-value	Taxonomic Group	*n*	*N*	*P*-value
2	A	Unpaired A	Eukarya 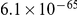 Microsporidia	59	59	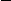	Bacteria	41	41	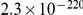
	Gap	Gap		136	145	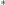		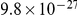 14	14	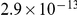
		Unpaired A	Bacteria	15	41	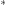	Eukarya 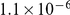 Microsporidia	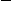 8	59	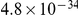
3	A			28	41	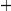	Archaea 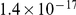 Microsporidia	11	11	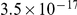
	Gap	Gap		12	14	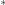		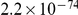 41	45	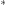
		Unpaired A					Bacteria	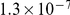 8	41	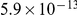
4	A		Proteobacteria	8	8	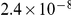	Firmicutes	5	5	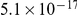
5			Microsporidia	16	31	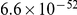	Archaea	39	49	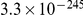
	Gap	Gap		191	387	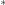		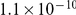 15	59	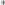
		Unpaired A					Microsporidia	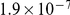 9	31	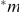

Probabilistic significance of the enrichment of the *m* = 200 positions with largest frequency increase or decrease in each of the nulceotide-specific segments of the significant eigenorganisms (except for the gap segments of the second, third and fifth eigenorganisms, where the largest nucleotide frequency decrease is shared by 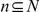 = 91, 100 and 199 positions, respectively) in sequence and structure motifs exclusively conserved in the corresponding taxonomic groups. The *P*-value of each enrichment is calculated assuming, for each nucleotide, hypergeometric probability distribution of the *N* conserved motifs among the *M* = 6636 positions, and of the subset of 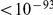 motifs among the subset of *m* positions. Exclusive sequence gap conservation is defined as conservation of gaps within at least 80% of the organisms of the corresponding taxonomic group but in less than 20% of the remaining organisms. Exclusive unpaired A nucleotide conservation is defined as conservation of an adenosine within at least 80% of the organisms of the group but in less than 20% of the remaining organisms, together with greater frequency of unpaired nucleotides within the group rather than among the remaining organisms.

**Figure 3:**
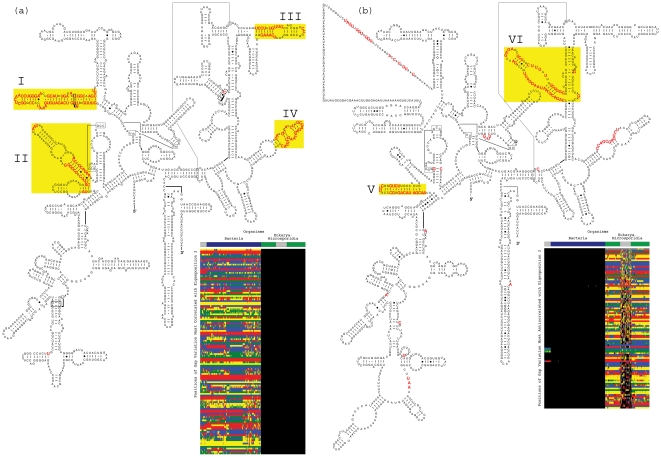
Sequence gaps exclusive to Eukarya or Bacteria 16S rRNAs. The second most significant 16S eigenorganism identifies gaps exclusively conserved in either the Eukarya excluding the Microsporidia or the Bacteria (Table 3) that map out known as well as previously unrecognized, entire substructures deleted or inserted, respectively, in the Eukarya relative to the Bacteria. (*a*) The 124 positions with largest increase in relative nucleotide frequency in the gap segment of the second eigenorganism, i.e., the 124 positions of gap variation across the organisms most correlated with the second eigenposition, map out the exclusively conserved known substructures [31] I and II and the previously unrecognized substructures III and IV in the secondary structure model of the bacterium *E. coli* [14]. These 124 positions are also displayed in the inset raster, ordered by their significance, with the most significant position at the top. The nucleotides are color-coded A (red), C (green), G (blue), U (yellow), unknown (gray) and gap (black). The color bars highlight the taxonomic groups that are differentiated by the second 16S eigenposition and eigenorganism, i.e., the Eukarya excluding the Microsporidia and the Bacteria. (*b*) Of the 100 positions of gap variation across the organisms most anticorrelated with the second eigenposition, 99 map out the substructures V and VI in the secondary structure model of the eukaryote *S. cerevisiae*. The 100th position is an unknown nucleotide at the 3′-end of the molecule, which is not displayed. These 100 positions are also displayed in the inset raster.

The positions identified by the eigenorganisms include entire substructures inserted or deleted in the structure of one taxonomic group relative to another. Consider for example the 124 positions with largest nucleotide frequency increase in the gap segment of the second most significant 16S eigenorganism, i.e., the positions for which the frequency of gaps across the organisms is most correlated with the second eigenposition. These positions are enriched in sequence gaps conserved in the Eukarya excluding the Microsporidia (Figure S4*a* in Appendix S1). The 100 positions with largest frequency decrease are enriched in gaps conserved in the Bacteria (Figure S4*b* in Appendix S1). Both *P*-values 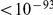. Mapped onto the secondary structure models of the bacterium *E. coli* and the eukaryote *S. cerevisiae* [14], these positions map out known as well as previously unrecognized insertions and deletions of not only isolated nucleotides but entire substructures in the Eukarya with respect to the Bacteria [31] (Figure 3). Similarly, the positions identified by the gap segment of the second 23S eigenorganism map out entire substructures inserted and deleted in 23S rRNAs of the Eukarya relative to the Bacteria (Figures S5 and S6 in Appendix S1).

In addition, the eigenorganisms identify adenosines that are unpaired in the rRNA secondary structure and are conserved exclusively in the respective taxonomic groups. The majority of these adenosines participate in tertiary structure interactions, and some also map to the same substructures. Previous comparative studies observed that the A nucleotides are usually unpaired in the secondary structure models of rRNA, while most of the C, G and U nucleotides are base-paired [6]. It was also noted that these unpaired adenosines are especially abundant in tertiary structure motifs, such as tetraloops, i.e., the four-base hairpin loops that cap many rRNA double helices [7]. Experimental observations of intra- and intermolecular interactions involving these loops and other motifs rich in unpaired adenosines [20] suggested a role for these unpaired nucleotides in a universal mode of RNA helical packing [21], [22] as well as in the accuracy and specificity of the translational function of the rRNA protein synthesis [23]–[26].

**Figure 4:**
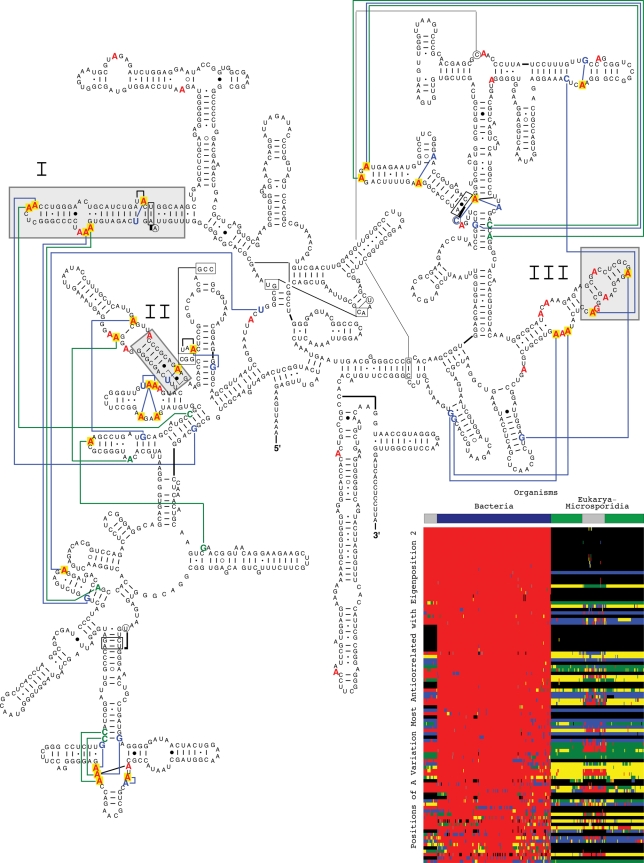
Unpaired adenosines exclusive to Bacteria 16S rRNAs. The 100 positions identified in the A nucleotide segment of the second 16S eigenorganism with the largest decrease in relative nucleotide frequency include all 50 positions (red) in the alignment with unpaired A nucleotides exclusively conserved in the Bacteria. Of these 50 positions, 28 (yellow) map to known tertiary interactions in the crystal structure of the bacterium *T. thermophilus* [8], plotted on the secondary structure model of the bacterium *E. coli* [14]. These include 22 base-base interactions (blue) and eight base-backbone interactions (green). Of the 50 positions of unpaired A nucleotides exclusively conserved in the Bacteria, 13 correspond to gaps exclusively conserved in the Eukarya excluding the Microsporidia. These 13 positions map to the entire 16S rRNA substructures that are deleted in the Eukarya with respect to the Bacteria (gray), identified by the gap segment of the second eigenorganism (Figure 3). These 100 positions identified in the A nucleotide segment of the second 16S eigenorganism are displayed in the inset raster, ordered by their significance, with the most significant position at the top. The nucleotides are color-coded A (red), C (green), G (blue), U (yellow), unknown (gray) and gap (black). The color bars highlight the taxonomic groups that are differentiated by the second eigenposition and eigenorganism, i.e., the Eukarya excluding the Microsporidia and the Bacteria.

We find the positions with largest nucleotide frequency increase in the A segment of the second 16S eigenorganism to be enriched in unpaired adenosines, which are exclusively conserved in the Eukarya excluding the Microsporidia (Figures S7 and S8 in Appendix S1), whereas the positions with largest decrease include all 50 unpaired adenosines exclusively conserved in the Bacteria (Figure 4). Both *P*-values 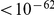. Note that 13 of these 50 unpaired adenosines exclusively conserved in the Bacteria, map to rRNA substructures that are deleted in Eukarya, with the corresponding *P*-value 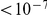. Of these 50 unpaired adenosines, the crystal structure of the bacterium *T. thermophilus* [8] reveals that 28 are involved in tertiary base-base or base-backbone interactions. Similarly, the positions identified by the A nucleotide segment of the second 23S eigenorganism are enriched in unpaired adenosines exclusively conserved in the Eukarya excluding the Microsporidia and in the Bacteria, most of which map to the substructures inserted or deleted in the Eukarya with respect to the Bacteria, respectively (Figures S5 and S9 in Appendix S1).

We find a similar enrichment of unpaired A nucleotides exclusively conserved in the taxonomic groups identified by the fourth through seventh 16S eigenpositions and by the third through fifth 23S eigenpositions. The 100 positions with largest frequency increase or decrease in the A nucleotide segment of the fourth, fifth, sixth or seventh 16S eigenorganism, i.e., the positions for which the A nucleotide frequency across the organisms is most correlated or anticorrelated, respectively, with the fourth, fifth, sixth or seventh eigenposition, include all or most of the unpaired A nucleotides exclusively conserved in either the Gamma Proteobacteria, Archaea, Rhodophyta, Alveolata or Fungi/Metazoa excluding the Microsporidia, respectively, with all *P*-values 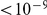. The 200 positions with largest frequency increase or decrease in the A nucleotide segment of the third, fourth or fifth 23S eigenorganism include all or most of the unpaired A nucleotides exclusively conserved in either the Proteobacteria, Firmicutes, Archaea or Microsporidia, respectively, with all *P*-values 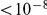.

These results demonstrate that an organism's evolutionary pathway is correlated and possibly also causally coordinated with insertions or deletions of entire rRNA substructures and unpaired adenosines, i.e., these structural motifs which are involved in rRNA folding and function are evolutionary degrees of freedom.

Third, we find that, two previously unknown coexisting subgenic relationships between Microsporidia [27]–[29] and Archaea [30], [31] are revealed in both the 16S and 23S rRNAs, a convergence and a divergence, that are conferred by insertions and deletions of these structural motifs. The Microsporidia are eukaryotic single cell intracellular parasites of small genomes, ribosomes and rRNAs, which lack mitochondria and most other membrane-bound cellular organelles. Early comparative studies of single rRNA molecules suggested that the Microsporidia are most dissimilar to all other eukaryotes [27], while more recent comparative studies of multiple genes revealed that Microsporidia are most similar to the Fungi [28], [29]. The Archaea are single cell prokaryotes of extremely small genomes. Archaeal rRNAs are more similar to bacterial rather than eukaryotic rRNAs. Archaeal ribosomal proteins, however, are more similar to eukaryotic rather than bacterial ribosomal proteins [30], [31].

**Figure 5:**
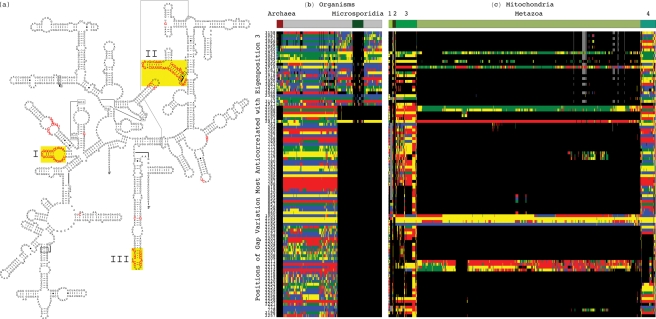
Sequence gaps exclusive to both Archaea and Microsporidia 16S rRNAs. The 100 positions identified in the gap segment of the third 16S eigenorganism with the largest decrease in relative nucleotide frequency map out entire substructures in the Bacteria 16S rRNAs that are convergently lost in the Archaea and the Microsporidia. (*a*) The 100 gaps conserved in both the Archaea and Microsporidia map to the entire substructures I–III in the secondary structure model of the bacterium *E. coli* [14]. (*b*) Raster display of the 100 positions of conserved gaps in both the Archaea and Microsporidia across the alignment. (*c*) Raster display of the same 100 positions across an alignment of 858 mitochondrial 16S rRNA sequences show gaps conserved in most Metazoa. The other groups of Eukarya represented in the mitochondrial alignment are Alveolata (1), Euglenozoa (2), Fungi (3) and Rhodophyta and Viridiplantae (4). The nucleotides are color-coded A (red), C (green), G (blue), U (yellow), unknown (gray) and gap (black). The color bars highlight the taxonomic groups.

In both 16S and 23S alignments, the third most significant eigenposition captures the similarities among the two taxonomic groups and correlates with decreased nucleotide frequency across both the Archaea and Microsporidia relative to all other organisms with the *P*-values 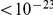 and 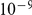, respectively. The 100 positions with largest nucleotide frequency decrease in the gap segment of the third 16S eigenorganism identify all six gaps exclusively conserved in both the Archaea and Microsporidia with the corresponding *P*-value 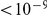. Mapped onto the secondary structure model of the bacterium *E. coli*, these 100 positions identify deletions of not only isolated nucleotides but entire substructures in the Archaea and Microsporidia with respect to the Bacteria (Figure 5*a*), indicating a convergent loss in both the Archaea and Microsporidia with respect to the Bacteria as well as the Eukarya. We observe that these same positions (Figure 5*b*) also identify nucleotides that are deleted in the metazoan mitochondrial 16S rRNA sequences (Figure 5*c*, and Dataset S7), suggesting that these similarities among the Archaea and Microsporidia may be explained by evolutionary forces that act to reduce the genome sizes of the Archaea and Microsporidia. Similarly, the 100 positions with largest nucleotide frequency decrease in the gap segment of the third 23S eigenorganism identify 41 of the 45 gaps and all 11 unpaired adenosines that are exclusively conserved in both the Archaea and Microsporidia with the corresponding *P*-values 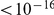 (Figures S10 and S11 in Appendix S1). Note that in the 16S alignment, the third eigenorganism also identifies positions of helices, i.e., base-paired nucleotides, that are exclusively conserved in both the Archaea and Microsporidia (Figure S12 in Appendix S1).

The fifth 16S and 23S eigenpositions both capture the dissimilarities between Archaea and Microsporidia and correlate with increased and decreased frequency across the Microsporidia and the Archaea, with the *P*-values 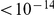 and 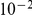, respectively. In the gap segment of the 16S fifth eigenorganism, the 100 positions with largest nucleotide frequency increase include seven of the 14 unpaired A nucleotides exclusively conserved in the Archaea, implying that these seven unpaired adenosines are exclusively missing in the Microsporidia (Figure S13 in Appendix S1). The 100 positions with largest nucleotide frequency decrease in the A nucleotide segment of this eigenorganism include all 14 unpaired A nucleotides exclusively conserved in the Archaea (Figure S14 in Appendix S1). These same positions in the mitochodrial 16S rRNA do not follow a trend similar to either the Archaea or the Microsporidia. Similarly, the gap segment of the 23S fifth eigenorganism identifies 191 of the 387 and 15 of the 59 sequence gaps exclusive to the Microsporidia and the Archaea, respectively, with both *P*-values 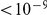, mapping out entire substructures deleted and inserted in the Microsporidia relative to the Archaea. The A nucleotide segment of this eigenorganism identifies 16 of the 31 and 39 of the 49 unpaired adenosines exclusively conserved in the Microsporidia and Archaea, respectively, with both *P*-values 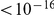 (Figures S15–S17 in Appendix S1).

Together, the third and fifth eigenpositions and eigenorganisms, in both the 16S and 23S rRNA alignments, reveal two previously unknown coexisting subgenic relationships of similarity and dissimilarity, i.e., convergence and divergence, between the Archaea and Microsporidia. These two relationships might correspond to two independent evolutionary pathways. The similarity among Microsporidia and Archaea in terms of their 16S and 23S rRNAs may be explained by evolutionary forces that act to reduce the genome sizes of these organisms. A single hierarchy cannot describe both these relationships of coexisting pathway-dependent similarity and dissimilarity.

These results support our hypothesis that even on the level of a single rRNA molecule, an organism's evolution is composed of multiple pathways due to concurrent evolutionary forces that act independently upon different rRNA degrees of freedom.

## Discussion

It was shown that the SVD provides a mathematical framework for the modeling of DNA microarray data, where the mathematical variables and operations represent biological reality [32]: The variables, significant patterns uncovered in the data, correlate with activities of cellular elements, such as regulators or transcription factors. The operations, such as classification, rotation or reconstruction in subspaces of these patterns, were shown to simulate experimental observation of the correlations and possibly even the causal coordination of these activities. Recent experimental results [16] verify a computationally predicted mechanism of regulation [15], [36], demonstrating that SVD modeling of DNA microarray data can be used to correctly predict previously unknown cellular mechanisms.

We now show that the mode-1 HOSVD, which is computed by using the SVD, provides a mathematical framework for the modeling of rRNA sequence alignments, independent of a-priori knowledge of the taxonomic groups and their relationships, or the rRNA structures, where the mathematical variables, significant eigenpositions and corresponding nucleotide-specific segments of eigenorganisms, represent multiple subgenic evolutionary relationships of convergence and divergence, some known and some previously unknown, and correlations with structural motifs, that are consistent with current biological understanding of the 16S and 23S rRNAs.

Our mode-1 HOSVD analyses of 16S and 23S rRNA sequence alignments support the hypothesis that even on the level of a single rRNA molecule, an organism's evolution is composed of multiple pathways due to concurrent evolutionary forces that act independently upon different rRNA degrees of freedom. These analyses demonstrate that entire rRNA substructures and unpaired adenosines, i.e., rRNA structural motifs which are involved in rRNA folding and function, are evolutionary degrees of freedom. These analyses also show that mode-1 HOSVD modeling of rRNA sequence alignments might be used to computationally predict evolutionary mechanisms, i.e., evolutionary pathways and the underlying structural changes that these pathways are correlated, possibly even coordinated with.

## Supporting Information

Appendix S1 A PDF format file, readable by Adobe Acrobat Reader.
(PDF)

Mathematica Notebook S1 **Mode-1 higher-order singular value decomposition (HOSVD) of the 16S rRNA alignment.** A Mathematica 8 code file, executable by Mathematica 8 and readable by Mathematica Player, freely available at http://www.wolfram.com/products/player/.
(SITX)

Mathematica Notebook S2 **Mode-1 higher-order singular value decomposition (HOSVD) of the 16S rRNA alignment.** A PDF format file, readable by Adobe Acrobat Reader.
(PDF)

Mathematica Notebook S3 **Mode-1 HOSVD of the 23S rRNA alignment.** A Mathematica 8 code file, executable by Mathematica 8 and readable by Mathematica Player, freely available at http://www.wolfram.com/products/player/.
(NB) Click here for additional data file.

Mathematica Notebook S4 **Mode-1 HOSVD of the 23S rRNA alignment.** A PDF format file, readable by Adobe Acrobat Reader.
(PDF)

Dataset S1 **Taxonomy annotations of the organisms in the 16S rRNA alignment.** A tab-delimited text format file, readable by both Mathematica and Microsoft Excel, reproducing the National Center for Biotechnology Information (NCBI) Taxonomy Browser [19] annotations of the 339 organisms in the 16S alignment.
(TXT)

Dataset S2
**16S rRNA alignment.** Tab-delimited text format files, readable by both Mathematica and Microsoft Excel, reproducing the alignment of 16S rRNA sequences from the Comparative RNA Website (CRW) [14], tabulating six sequence elements, i.e., A, C, G and U nucleotides, unknown (“N”) and gap (“
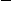
”), across the 339 organisms and the 3249 sequence positions.(TXT)

Dataset S3
**Base-pairing of the positions of the 16S rRNA alignment.** Tab-delimited text format files, readable by both Mathematica and Microsoft Excel, reproducing the base-pairing of the positions of the 16S rRNA alignment in the secondary structure models of the 16S sequences from the CRW [14], tabulating base-paired (“Y”) and unpaired (“N”) nucleotides as well as gaps (“
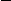
”), across the 339 organisms and the 3249 sequence positions.(TXT)

Dataset S4 **Taxonomy annotations of the organisms in the 23S rRNA alignment.** A tab-delimited text format file, readable by both Mathematica and Microsoft Excel, reproducing the NCBI Taxonomy Browser [19] annotations of the 75 organisms in the 23S alignment.
(TXT)

Dataset S5
**23S rRNA alignment.** Tab-delimited text format files, readable by both Mathematica and Microsoft Excel, reproducing the alignment of 23S rRNA sequences from the CRW [14], tabulating six sequence elements, i.e., A, C, G and U nucleotides, unknown (“N”) and gap (“
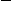
”), across the 75 organisms and the 6636 sequence positions.(TXT)

Dataset S6
**Base-pairing of the positions of the 16S rRNA alignment.** Tab-delimited text format files, readable by both Mathematica and Microsoft Excel, reproducing the base-pairing of the positions of the 23S rRNA alignment in the secondary structure models of the 23S sequences from the CRW [14], tabulating base-paired (“Y”) and unpaired (“N”) nucleotides as well as gaps (“
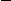
”), across the 75 organisms and the 6636 sequence positions.(TXT)

Dataset S7 **Mitochondrial 16S rRNA alignment with taxonomy annotations of the organisms.** Tab-delimited text format files, readable by both Mathematica and Microsoft Excel, reproducing the alignment of 858 mitochondrial 16S rRNA sequences from the CRW [14], tabulating six sequence elements across the 858 organisms and the 3249 sequence positions, as well as reproducing the NCBI Taxonomy Browser [19] annotations of the 858 organisms.
(TXT)
