# Supplementary material for: Tensor Decomposition Reveals Concurrent Evolutionary Convergences and Divergences and Correlations with Structural Motifs in Ribosomal RNA
Source: PLoS One. 2011 Apr 29;6(4):e18768. doi: 10.1371/journal.pone.0018768 (PMC3094155; doi:10.1371/journal.pone.0018768)
Supplement: Appendix S1 — A PDF format file, readable by Adobe Acrobat Reader. (PDF) [file pone.0018768.s001.pdf]

Supplementary Figures S1–S17

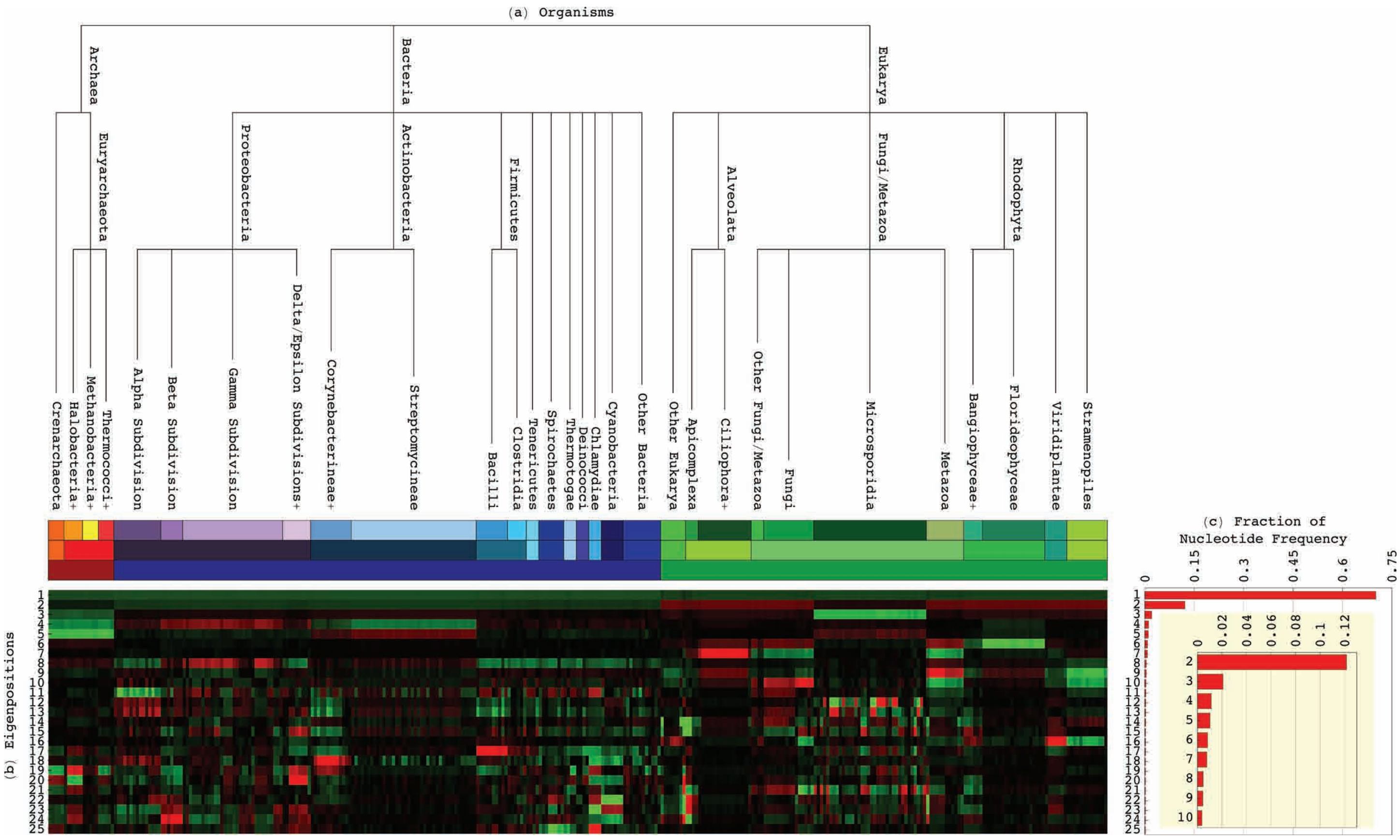

Figure S1. Significant 16S eigenpositions and their correlations with the NCBI Taxonomy Browser taxonomic groups. (*a*) Classification of the organisms in the alignment into taxonomic groups according to the top six hierarchical levels of the NCBI Taxonomy Browser [19] (Figure 2 and Table 1). (*b*) Raster display of the 25 most significant 16S eigenpositions, with increased frequency (red), no change in frequency (black) and decreased frequency (green) relative to the average frequency variation across the organisms, captured by the most significant, i.e., first 16S eigenposition. (*c*) Bar chart of the fractions of nucleotide frequency variation that the 25 most significant eigenpositions capture in the 16S alignment.

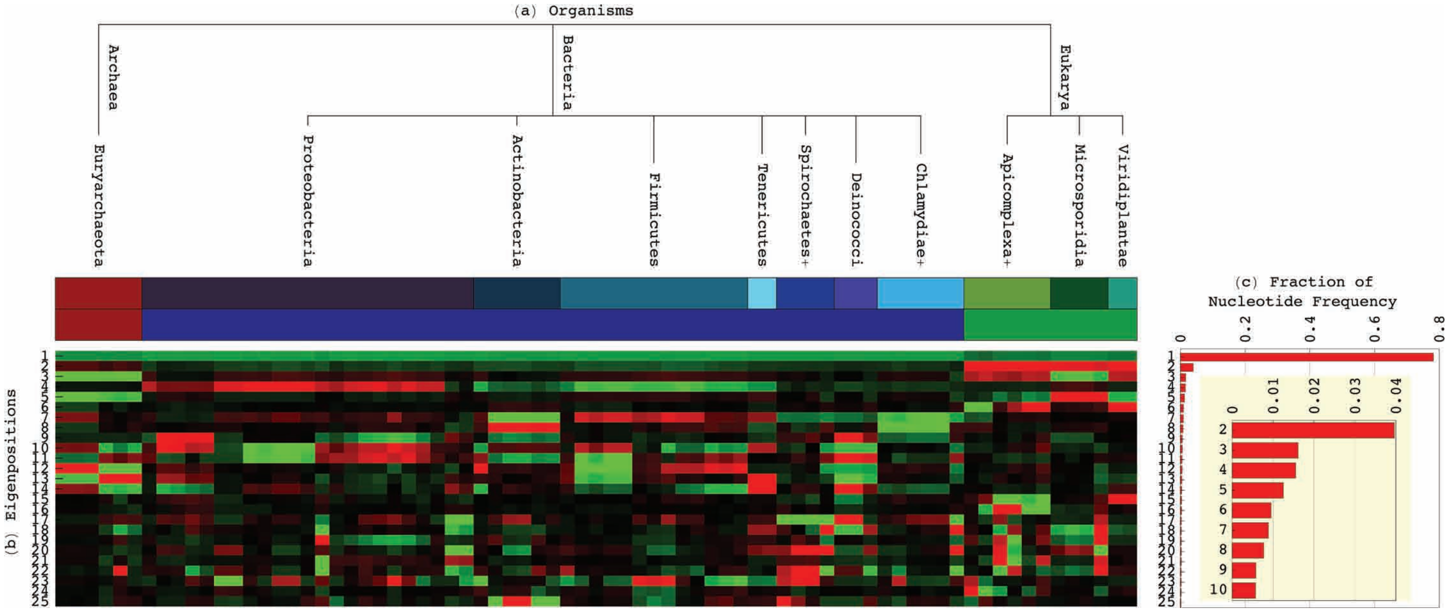

Figure S2. Significant 23S eigenpositions and their correlations with the NCBI Taxonomy Browser taxonomic groups. (*a*) Classification of the organisms in the alignment into taxonomic groups according to the top six hierarchical levels of the NCBI Taxonomy Browser [19] (Figure S3). (*b*) Raster display of the 25 most significant 23S eigenpositions, with increased frequency (red), no change in frequency (black) and decreased frequency (green) relative to the average frequency variation across the organisms, captured by the most significant, i.e., first 23S eigenposition. (*c*) Bar chart of the fractions of nucleotide frequency variation that the 25 most significant eigenpositions capture in the 23S alignment.

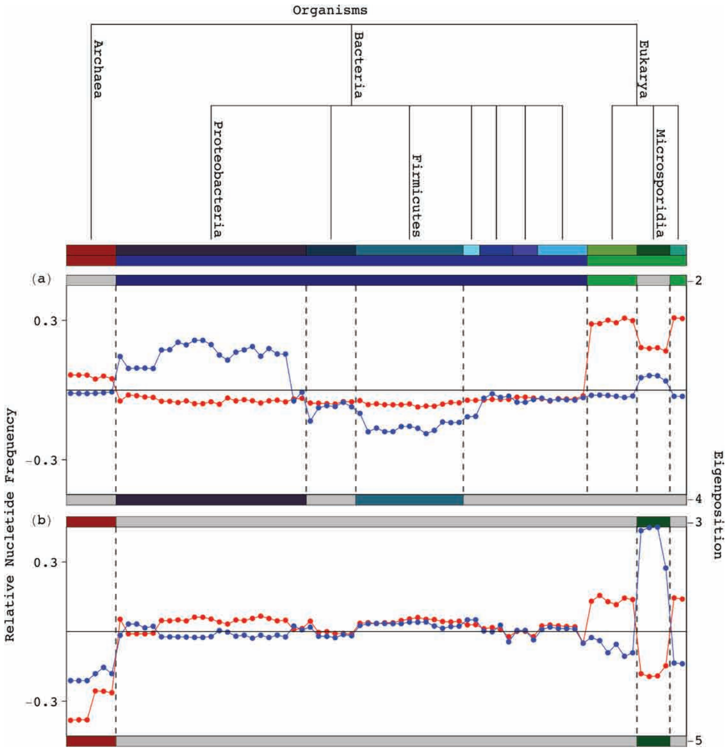

Figure S3. Significant 23S eigenpositions. Line-joined graphs of the second through fifth 23S eigenpositions, i.e., patterns of nucleotide frequency across the organisms, and their correlations with the taxonomic groups in the 23S alignment, classified according to the top six hierarchical levels of the NCBI Taxonomy Browser [19] (Figure S2). (*a*) The second most significant eigenposition (red) differentiates the Eukarya excluding the Microsporidia from the Bacteria, as indicated by the color bar (Table 2). The fourth (blue) distinguishes between the Proteobacteria and the Firmicutes. (*b*) The third (red) and fifth (blue) eigenpositions describe the similarities and dissimilarities among the Archaea and Microsporidia, respectively.

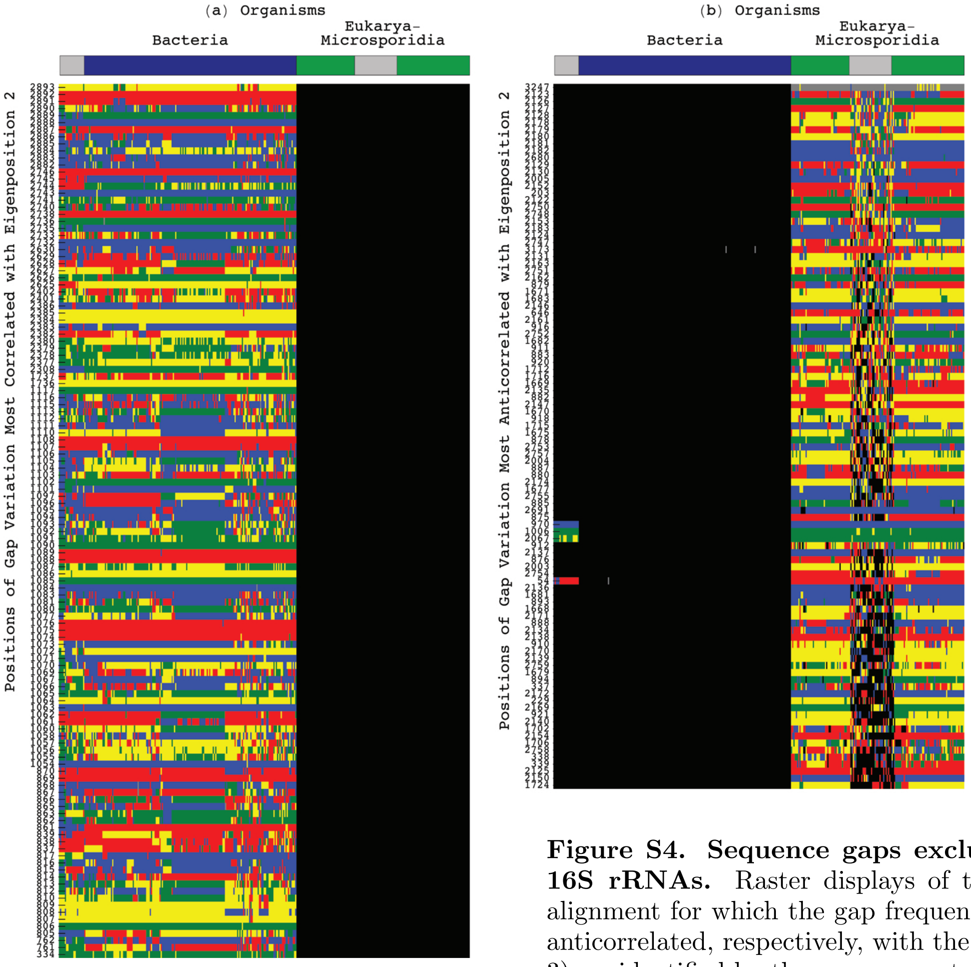

Figure S4. Sequence gaps exclusive to Eukarya or Bacteria 16S rRNAs. Raster displays of the 124 and 100 positions in the alignment for which the gap frequency variation is most correlated or anticorrelated, respectively, with the second 16S eigenposition (Figure 3), as identified by the gap segment of the second eigenorganism. The nucleotides are color-coded A (red), C (green), G (blue), U (yellow), unknown (gray) and gap (black). The color bars highlight the taxonomic groups that are differentiated by the second eigenposition and eigenorganism, i.e., the Eukarya excluding the Microsporidia and the Bacteria. (*a*) The 124 correlated positions display gaps exclusively conserved in the Eukarya. These include 13 of the 50 positions with unpaired A nucleotides exclusively conserved in the Bacteria (Figure 4). (*b*) The 100 anticorrelated positions display gaps exclusively conserved in the Bacteria. These include 8 of the 66 positions with unpaired A nucleotides exclusively conserved in the Eukarya (Figure S7).

Figure S5 (on p. A-4). Sequence gaps and unpaired adenosines exclusive to Eukarya excluding Microsporidia or Bacteria 23S rRNAs. The second most significant eigenorganism identifies gaps exclusively conserved in either the Eukarya excluding the Microsporidia or the Bacteria (Table 4) that map out entire substructures deleted or inserted, respectively, in the Bacteria relative to the Eukarya. The same eigenorganism also identifies unpaired adenosines, exclusively conserved in either the Eukarya excluding the Microsporidia or the Bacteria, some of which map to the same substructures. (*a*) The 200 positions with largest increase in relative nucleotide frequency in the gap segment of the second eigenorganism, i.e., the 200 positions of gap variation across the organisms most correlated with the second eigenposition (green), map out entire substructures in the secondary structure model of the bacterium *E. coli* (yellow). The 200 positions with largest frequency decrease in the A nucleotide segment of the same eigenorganism, identify all 41 unpaired A nucleotides that are exclusively conserved in the Bacteria (red). Of these, 15 correspond to gaps conserved in the Eukarya excluding the Microsporidia. (*b*) The 91 positions of gap variation across the organisms most anticorrelated with the second eigenposition (green) map out entire substructures in the secondary structure model of the eukaryote *S. cerevisiae* (yellow). The 200 positions with largest frequency increase in the A nucleotide segment of the same eigenorganism, identify all 59 unpaired A nucleotides that are exclusively conserved in the Eukarya excluding the Microsporidia (red). Of these, eight correspond to gaps conserved in the Bacteria.

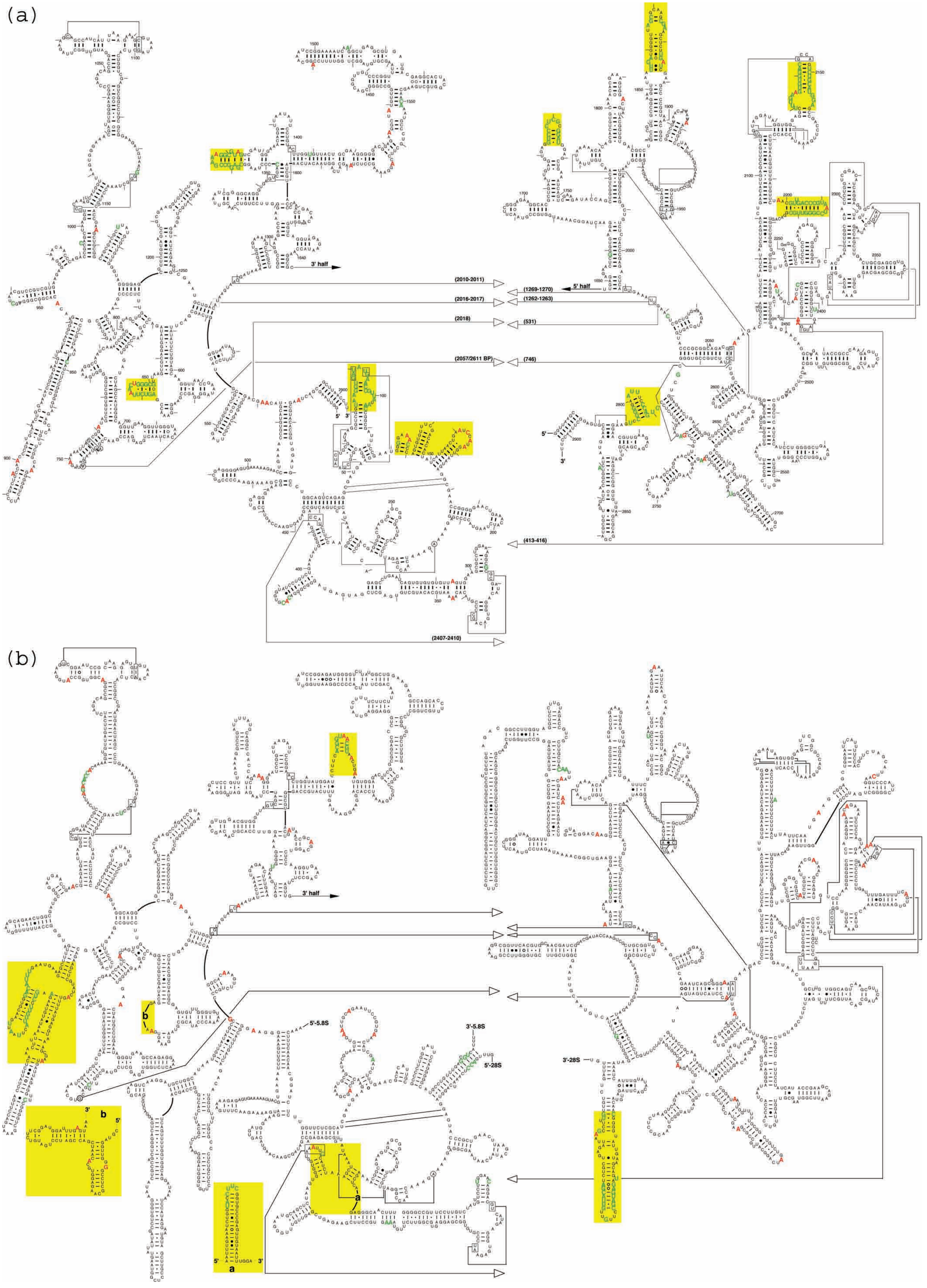

Figure S5 (Captions on p. A-3).

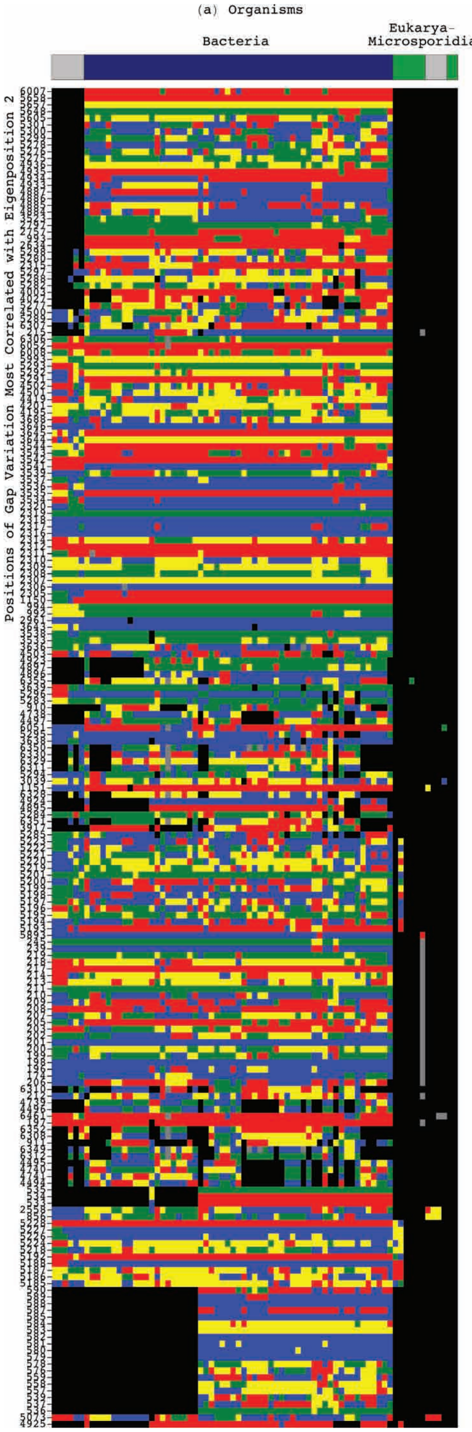

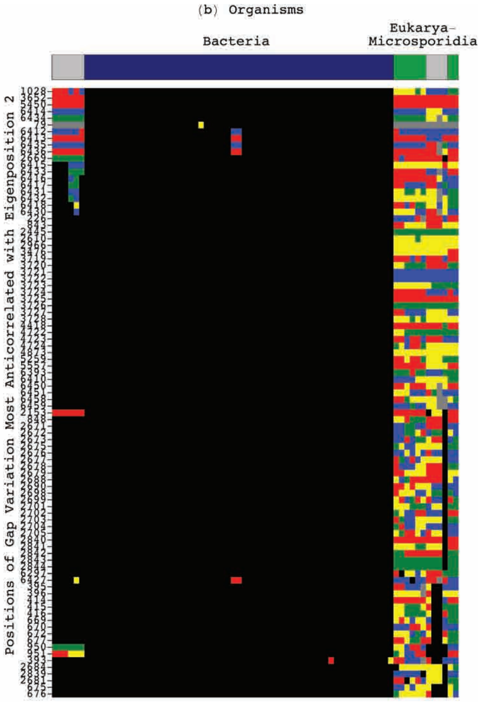

Figure S6. Sequence gaps exclusive to Eukarya excluding Microsporidia or Bacteria 23S rRNAs. Raster displays of the 200 and 91 positions in the 23S alignment for which the gap frequency variation is most correlated or anticorrelated, respectively, with the second 23S eigenposition (Figure S3), as identified by the gap segment of the second eigenorganism. The nucleotides are color-coded A (red), C (green), G (blue), U (yellow), unknown (gray) and gap (black). The color bars highlight the taxonomic groups that are differentiated by the second 23S eigenposition and eigenorganism, i.e., the Eukarya excluding the Microsporidia and the Bacteria (Table 4). (*a*) The 200 correlated positions display gaps exclusively conserved in the Eukarya. These include 15 of the 41 positions with unpaired A nucleotides exclusively conserved in the Bacteria. (*b*) The 91 anticorrelated positions display gaps exclusively conserved in the Bacteria. These include eight of the 59 positions with unpaired A nucleotides exclusively conserved in the Eukarya excluding the Microsporidia.

Figure S7 (on p. A-6). Adenosines exclusive to Eukarya excluding Microsporidia 16S rRNAs. The 100 positions identified in the A nucleotide segment of the second 16S eigenorganism with the largest increase in relative nucleotide frequency include 48 (red) of the 66 positions in the alignment with unpaired A nucleotides exclusively conserved in the Eukarya excluding the Microsporidia (Table 3) plotted on the secondary structure model of *S. cerevisiae* [14]. Eight of these 48 positions correspond to gaps conserved exclusively in the Bacteria, and map to the 16S rRNA substructures that are inserted in the Eukarya with respect to the Bacteria, and identified by the gap segment of the second eigenorganism (Figure 3). These 100 positions are displayed in the inset raster, ordered by their significance, with the most significant position at the top. The color bars highlight the Eukarya excluding the Microsporidia and the Bacteria.

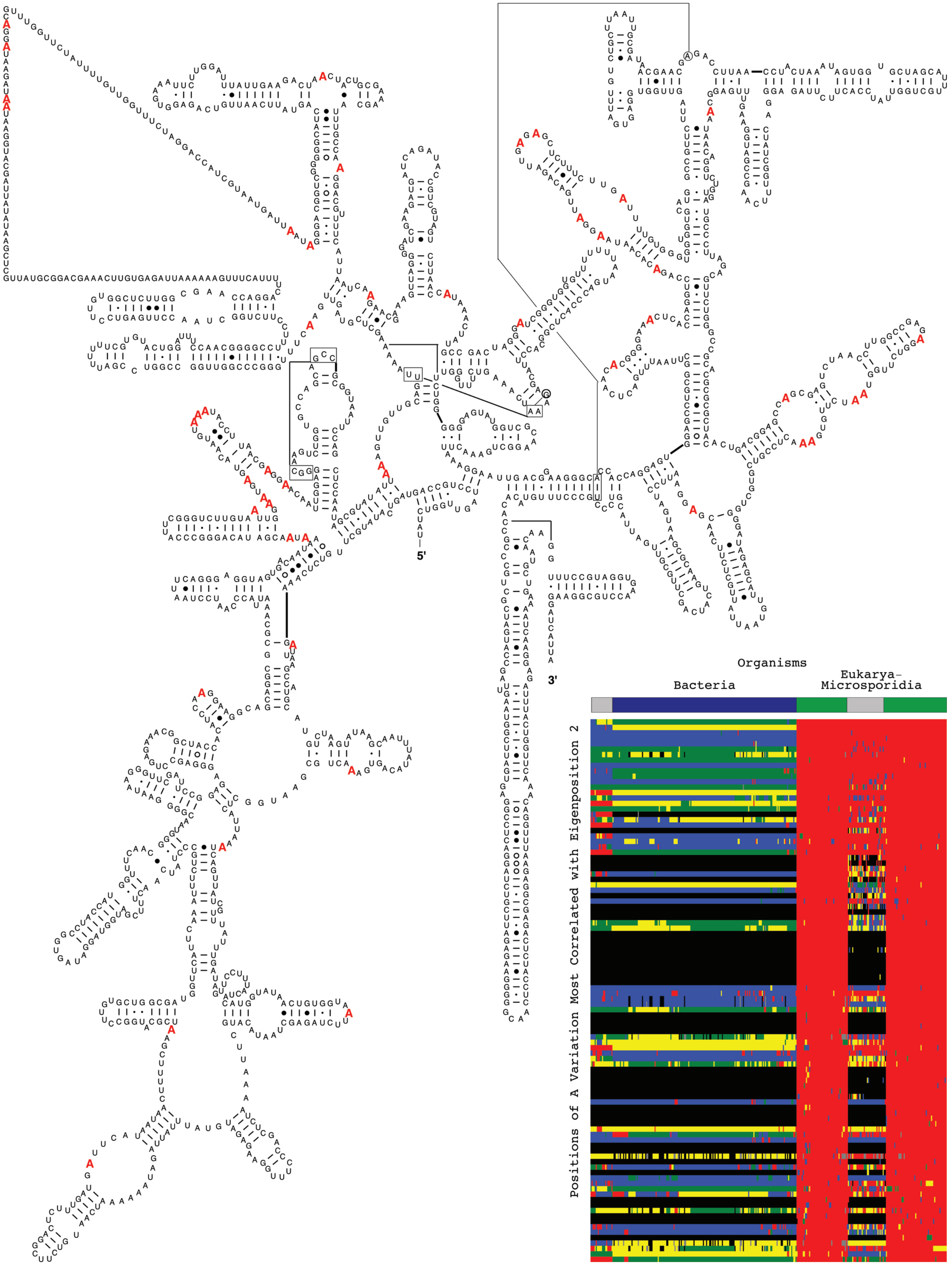

Figure S7 (captions on p. A-5).

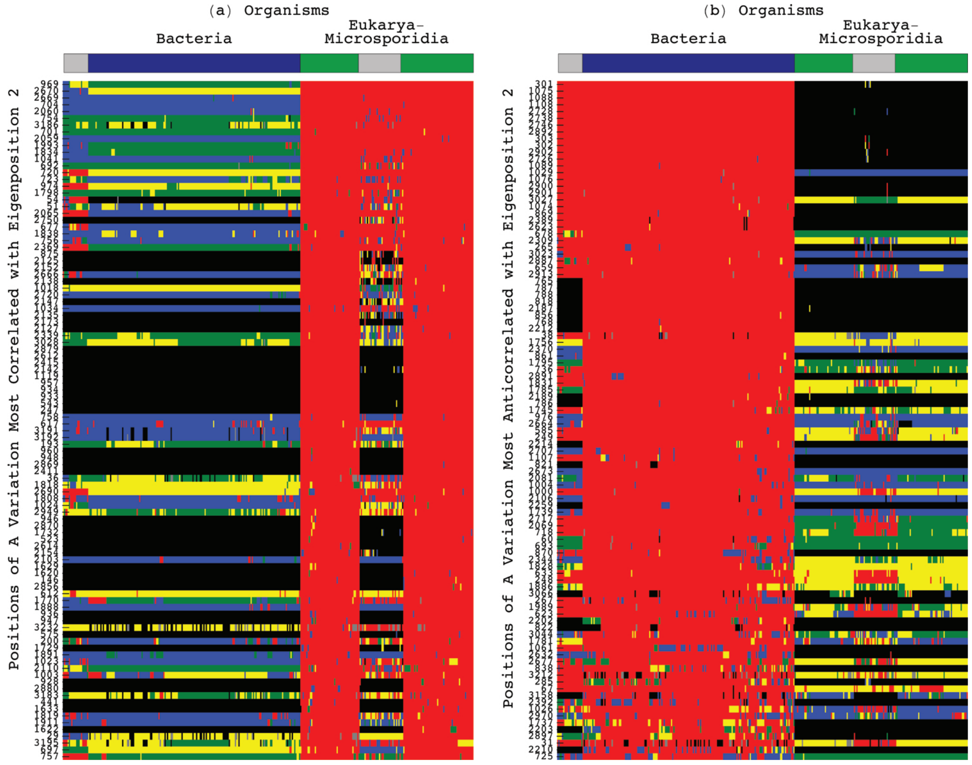

Figure S8. Adenosines exclusive to Eukarya excluding Microsporidia or Bacteria 16S rRNAs. Raster displays of the 100 positions in the alignment for which the A nucleotide frequency variation is most correlated or anticorrelated with the second 16S eigenposition, as identified by the A segment of the second 16S eigenorganism. The color bars highlight the Eukarya excluding the Microsporidia and the Bacteria. (*a*) The 100 correlated positions include 48 of the 66 unpaired A nucleotides exclusively conserved in the Eukarya excluding the Microsporidia (Figure S7). (*b*) The 100 anticorrelated positions include all 50 unpaired A nucleotides exclusively conserved in the Bacteria (Figure 4).

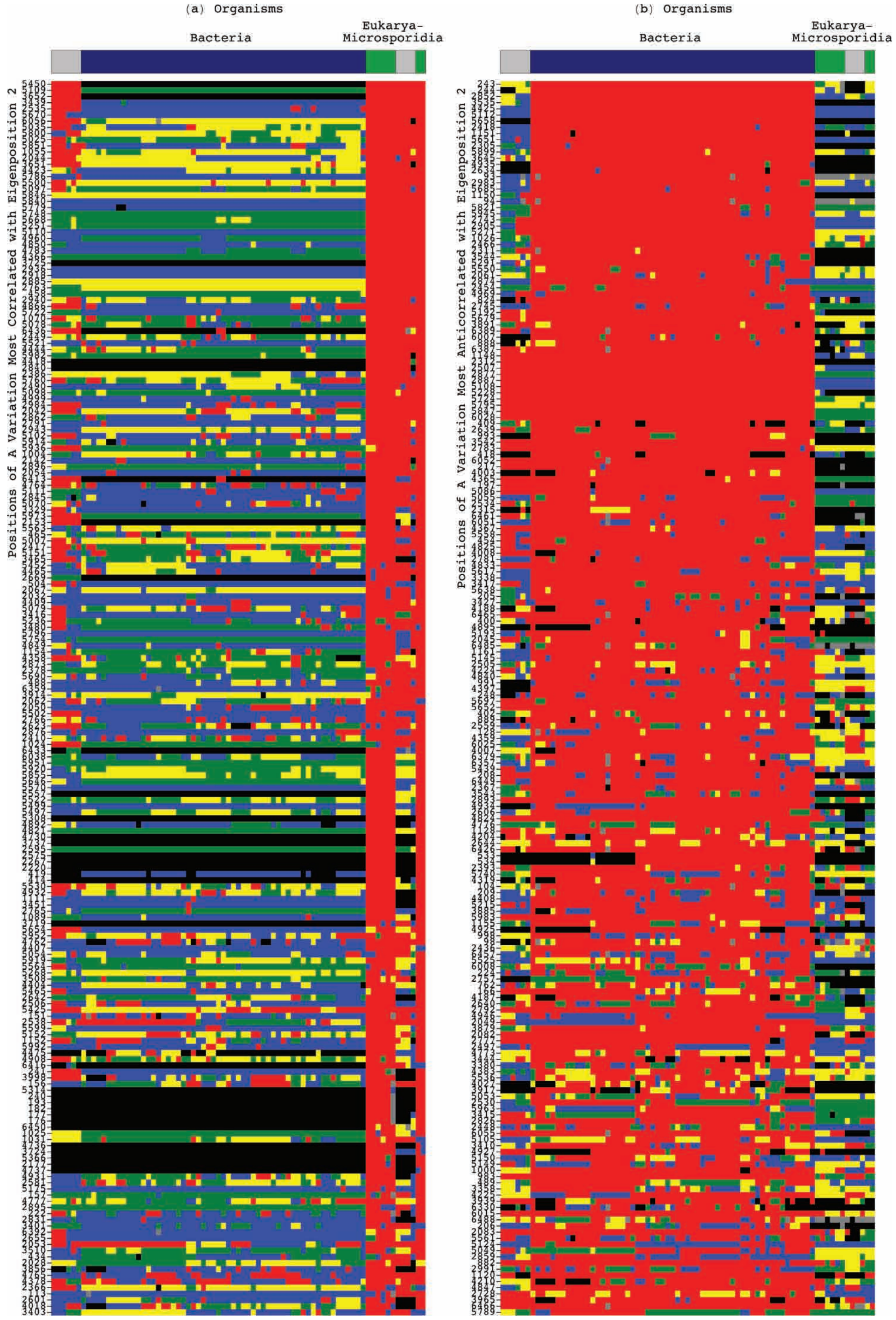

Figure S9. Adenosines exclusive to Eukarya excluding Microsporidia or Bacteria 23S rRNAs. Raster displays of the 200 positions in the 23S alignment for which the A nucleotide frequency variation is most correlated or anticorrelated with the second 23S eigenposition, as identified by the A segment of the second 23S eigenorganism (Figure S5). The color bars highlight the taxonomic groups of the Eukarya excluding the Microsporidia and the Bacteria. (*a*) The 200 correlated positions include all 59 unpaired A nucleotides exclusively conserved in the Eukarya excluding the Microsporidia. (*b*) The 200 anticorrelated positions include all 41 unpaired A nucleotides exclusively conserved in the Bacteria.

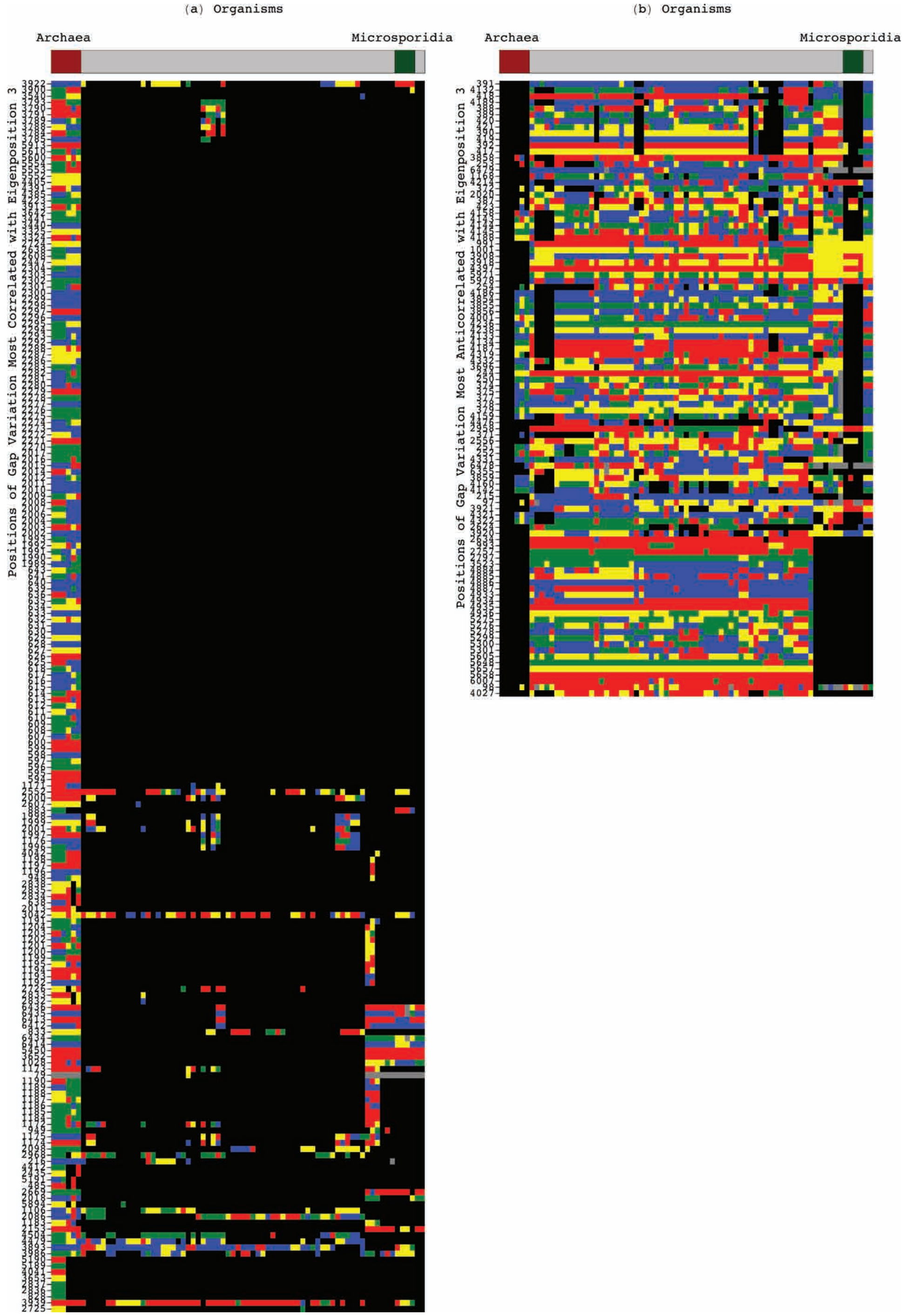

Figure S10. Sequence gaps exclusive to Bacteria or Archaea and Microsporidia 23S rRNAs. Raster displays of the 200 and 100 positions in the 23S alignment for which the gap frequency variation is most correlated or anticorrelated, respectively, with the third 23S eigenposition, as identified by the gap segment of the third 23S eigenorganism. The color bars highlight the Archaea and Microsporidia, the groups that are differentiated by the third eigenposition and eigenorganism from all other organisms in the alignment. (*a*) The 200 correlated positions include gaps conserved in the Bacteria. (*b*) The 100 anticorrelated positions display gaps exclusively conserved in the Archaea and Microsporidia. These 100 anticorrelated positions include 8 of the 41 unpaired A nucleotides exclusively conserved in the Bacteria (Figure S5*a*).

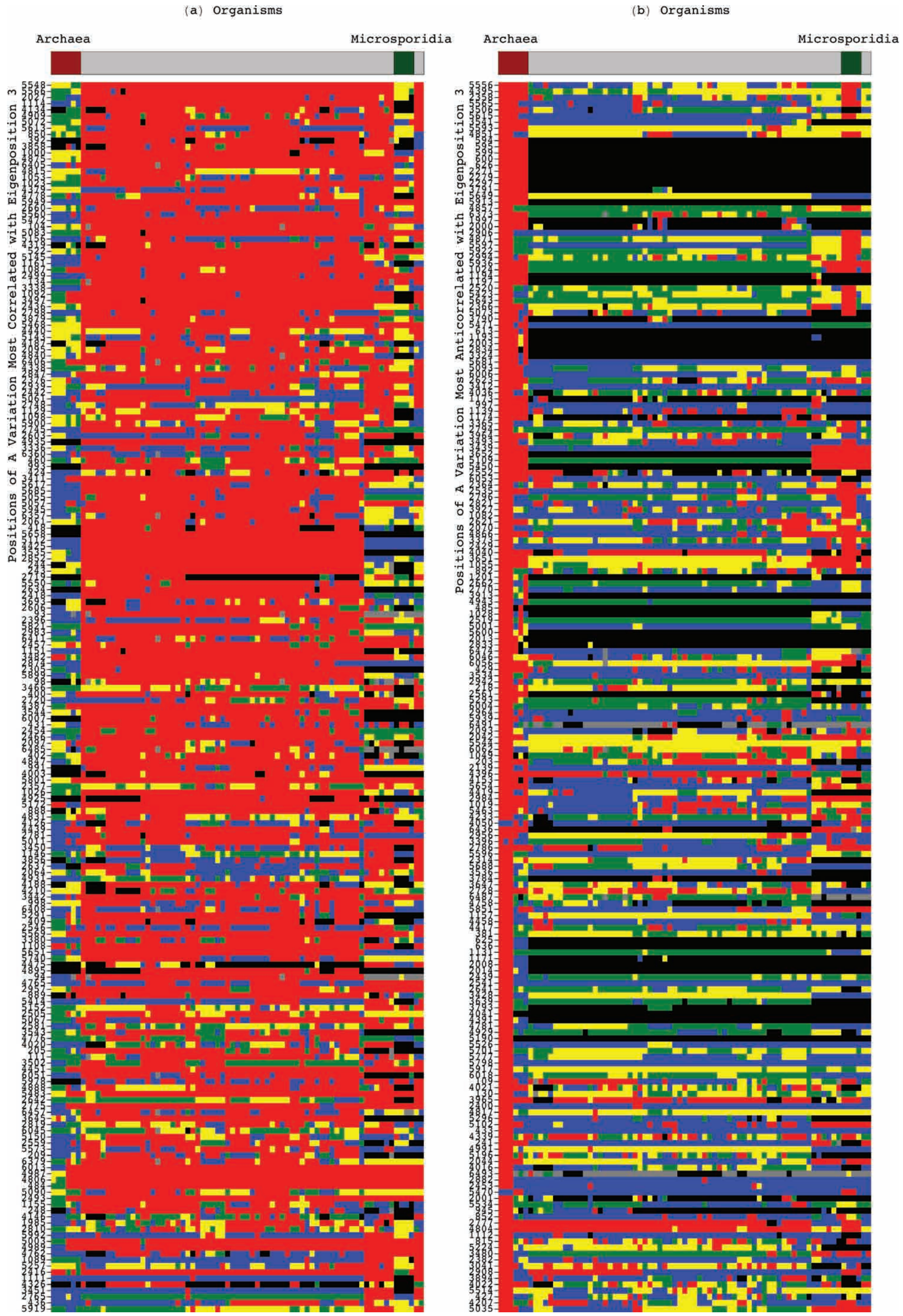

Figure S11. Adenosines exclusive to Bacteria or Archaea and Microsporidia 23S rRNAs. Raster displays of the 200 positions in the 23S alignment for which the A nucleotide frequency variation is most correlated or anticorrelated with the third 23S eigenposition, as identified by the A segment of the third 23S eigenorganism. The color bars highlight the Archaea and Microsporidia, the groups that are differentiated by the third eigenposition and eigenorganism from all other organisms in the alignment. (*a*) The 200 correlated positions include 28 of 41 unpaired A nucleotides exclusively conserved in the Bacteria. (*b*) The 200 anticorrelated positions include all 11 unpaired A nucleotides exclusively conserved in the Archaea and Microsporidia.

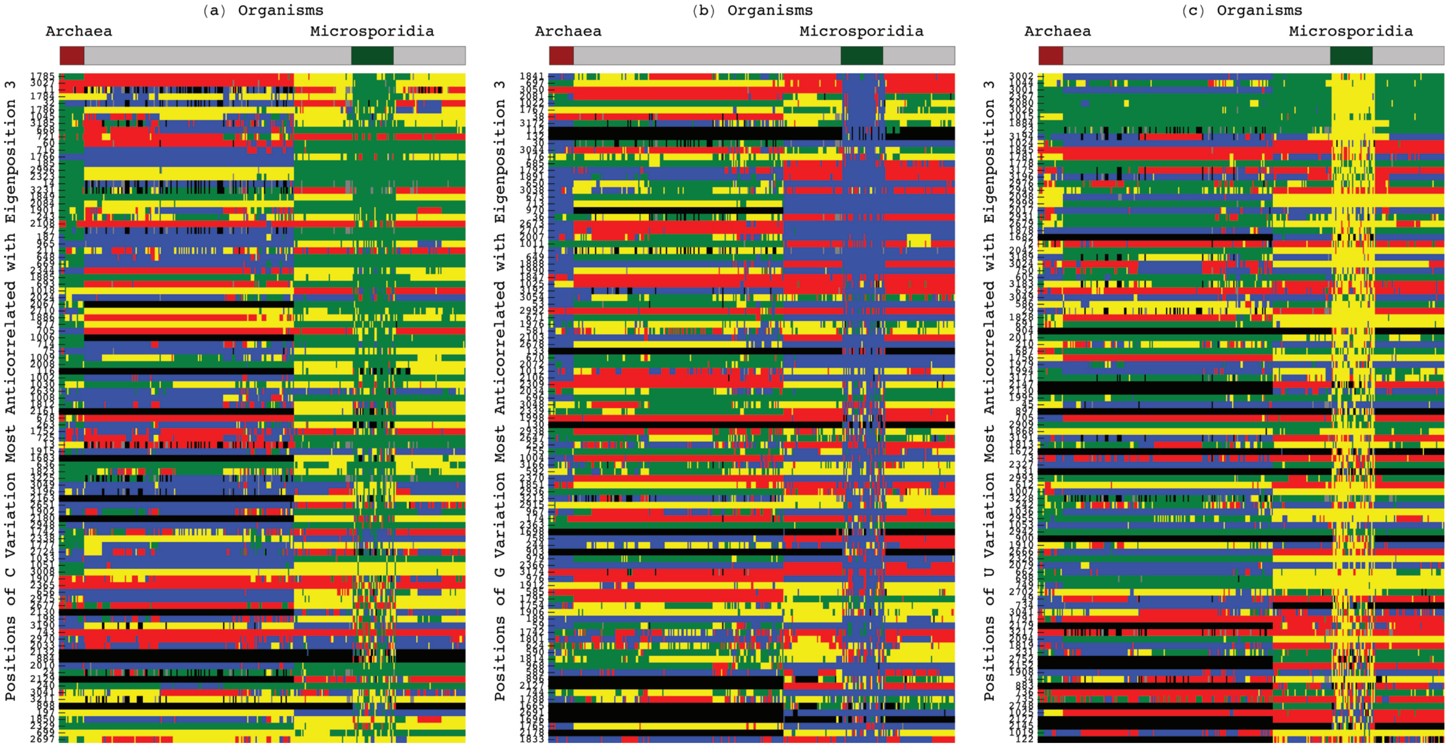

Figure S12. Helices exclusive to Archaea and Microsporidia 16S rRNAs. Raster displays of the 100 positions each, identified in the (*a*) C, (*b*) G and (*c*) U nucelotide segments of the third 16S eigenorganism with the largest decrease in relative nucleotide frequency. These positions are enriched in helices, i.e., base-paired nucleotides, exclusively conserved in both the Archaea and Microsporidia, with the P-values $< 10^{-9}$ (Table 3). The color bars highlight the Archaea and Microsporidia, the groups that are differentiated by the third 16S eigenposition and eigenorganism from all other organisms in the alignment.

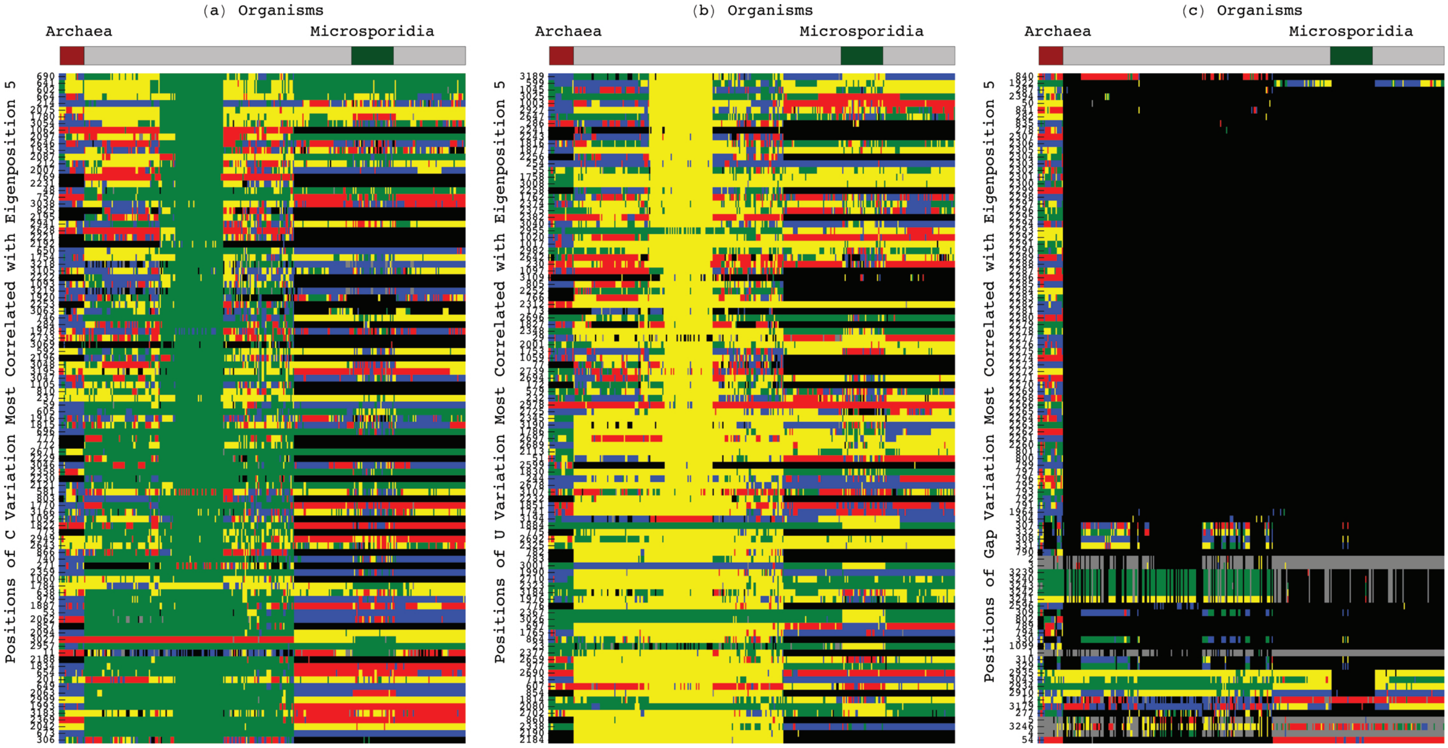

Figure S13. Helices and gaps exclusive to Microsporidia vs. Archaea 16S rRNAs. Raster displays of the 100 positions each identified in the (*a*) C and (*b*) U nucleotide and (*c*) gap segments of the fifth 16S eigenorganism with the largest increase in relative nucleotide frequency. The color bars highlight the taxonomic groups that are differentiated by the fifth eigenposition and eigenorganism, i.e, the Archaea and Microsporidia. The 100 positions with largest nucleotide frequency increase in the C and U segments of the fifth eigenorganism are enriched in helices exclusively conserved in the Microsporidia. In the gap segment, the 100 positions with largest nucleotide frequency increase include seven of the 14 unpaired A nucleotides exclusively conserved in the Archaea, implying that these seven unpaired adenosines are exclusively missing in the Microsporidia.

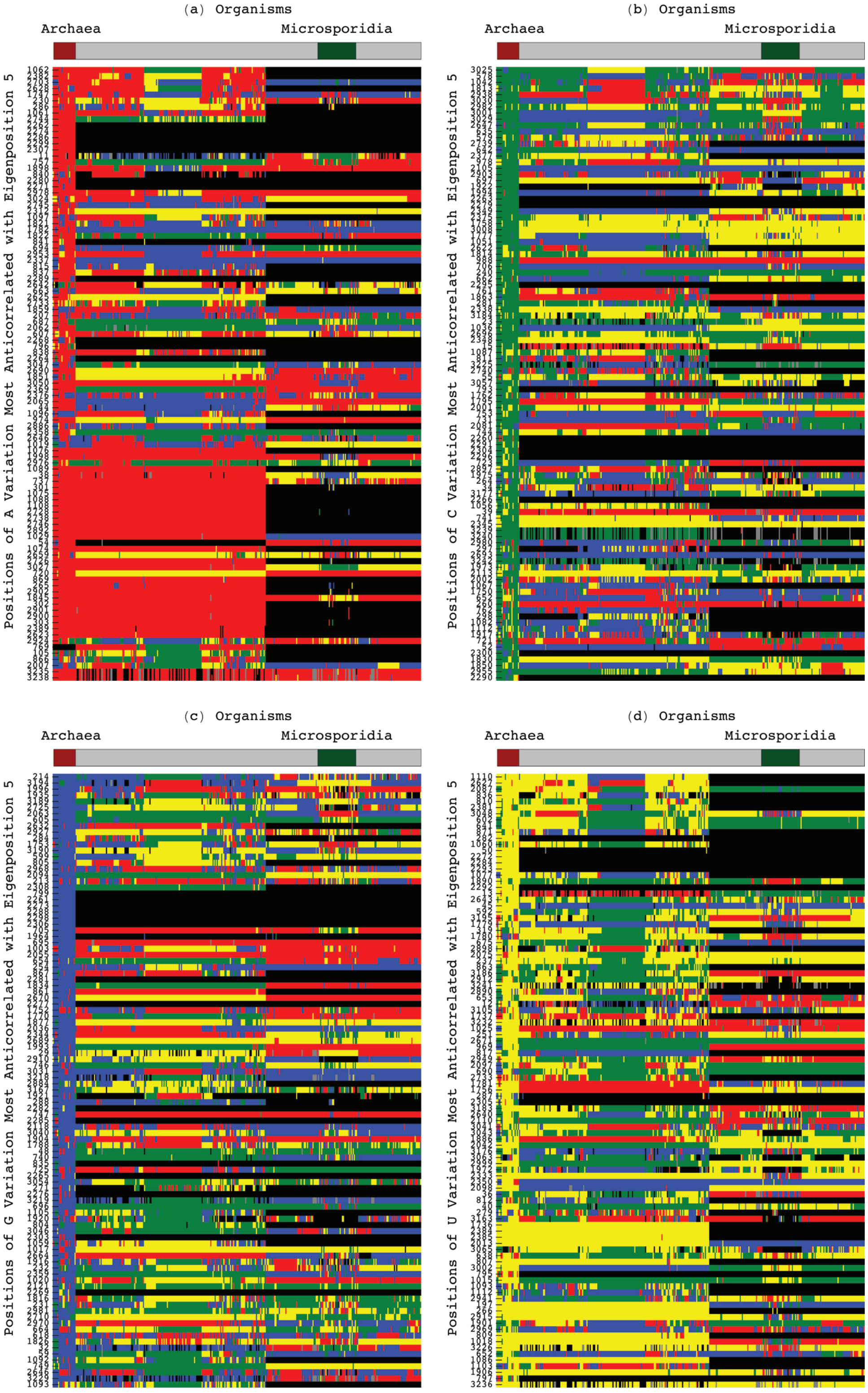

Figure S14 (captions on p. A-14).

Figure S14 (p. A-13). Helices and adenosines exclusive to Archaea vs. Microsporidia 16S rRNAs. Raster displays of the 100 positions each identified in the (*a*) A, (*b*) C, (*c*) G and (*d*) U nucleotide segments of the fifth 16S eigenorganism with the largest decrease in relative nucleotide frequency. The nucleotides are color-coded A (red), C (green), G (blue), U (yellow), unknown (gray) and gap (black). The color bars highlight the taxonomic groups that are differentiated by the fifth eigenposition and eigenorganism, i.e, the Archaea and Microsporidia. The 100 positions with largest nucleotide frequency decrease in the A segment of the fifth 16S eigenorganism include all 14 unpaired A nucleotides exclusively conserved in the Archaea. In the C, G and U segments, the 100 positions with largest nucleotide frequency decrease are enriched in helices exclusively conserved in the Archaea with the P-values $< 10^{-8}$ (Table 3).

Figure S15 (p. A-15). Sequence gaps and unpaired adenosines exclusive to Microsporidia vs. Archaea 23S rRNAs. The fifth most significant 23S eigenorganism identifies gaps exclusively conserved in either the Microsporidia or the Archaea (Table 4) that map out entire substructures deleted or inserted, respectively, in the Microsporidia relative to the Archaea. The same eigenorganism also identifies unpaired adenosines, exclusively conserved in either the Microsporidia or the Archaea, some of which map to the same substructures. (*a*) The 200 positions with largest increase in relative nucleotide frequency in the gap segment of the fifth eigenorganism, i.e., the 200 positions of gap variation across the organisms most correlated with the fifth eigenposition (green), map out entire substructures in the secondary structure model of the archaeum *M. jannaschii* (yellow). The 200 positions with largest frequency decrease in the A nucleotide segment of the same eigenorganism identify 39 of the 49 unpaired A nucleotides that are exclusively conserved in the Archaea (red). (*b*) The 199 positions of gap variation across the organisms most anticorrelated with the fifth eigenposition (green) map out entire substructures in the secondary structure model of the microsporidium *E. cuniculi* (yellow). The 200 positions with largest frequency increase in the A nucleotide segment of the same eigenorganism identify 16 of the 31 unpaired A nucleotides that are exclusively conserved in the Microsporidia (red). Of these, nine correspond to gaps conserved in the Archaea.

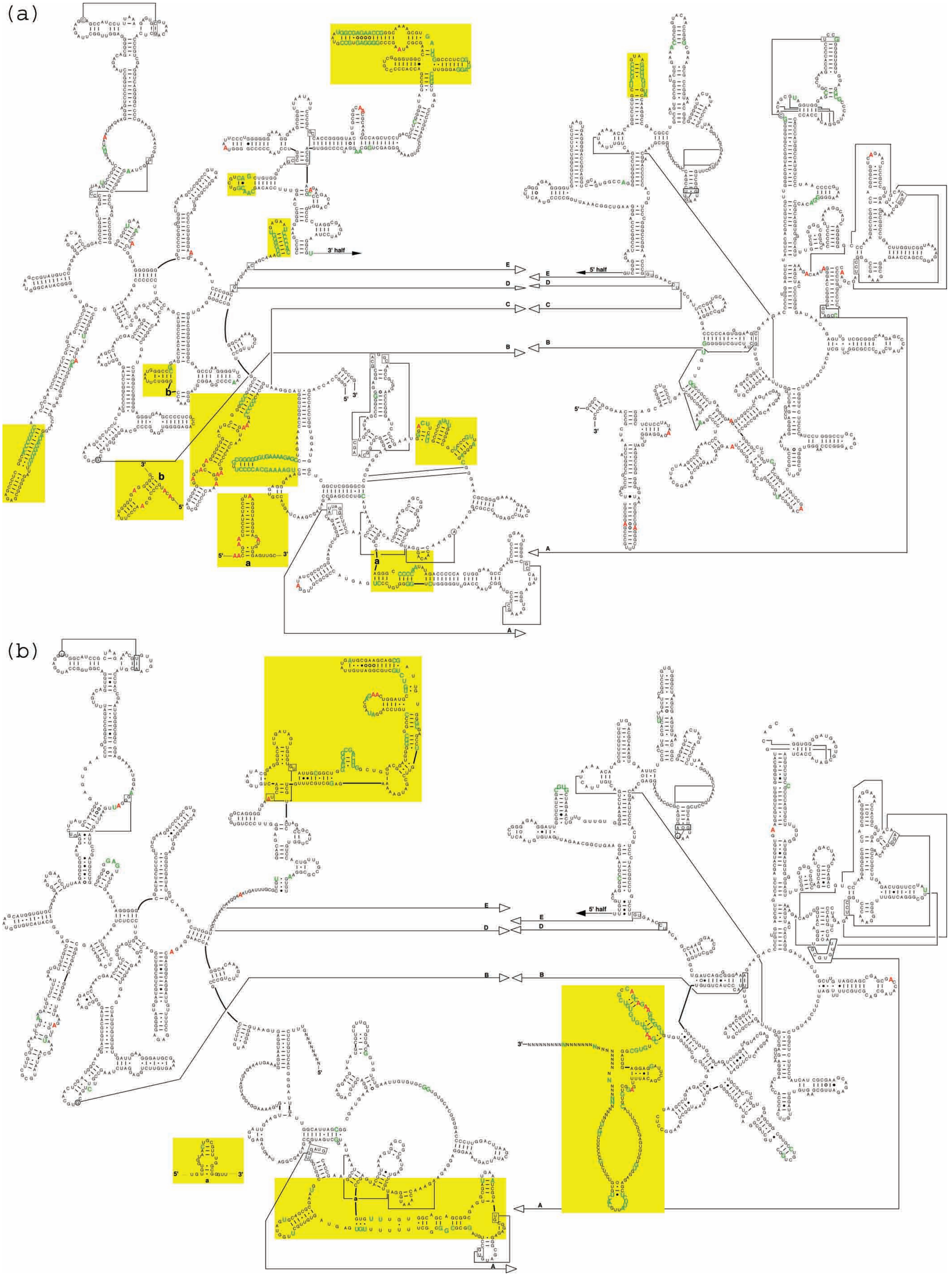

Figure S15 (captions on p. A-14).

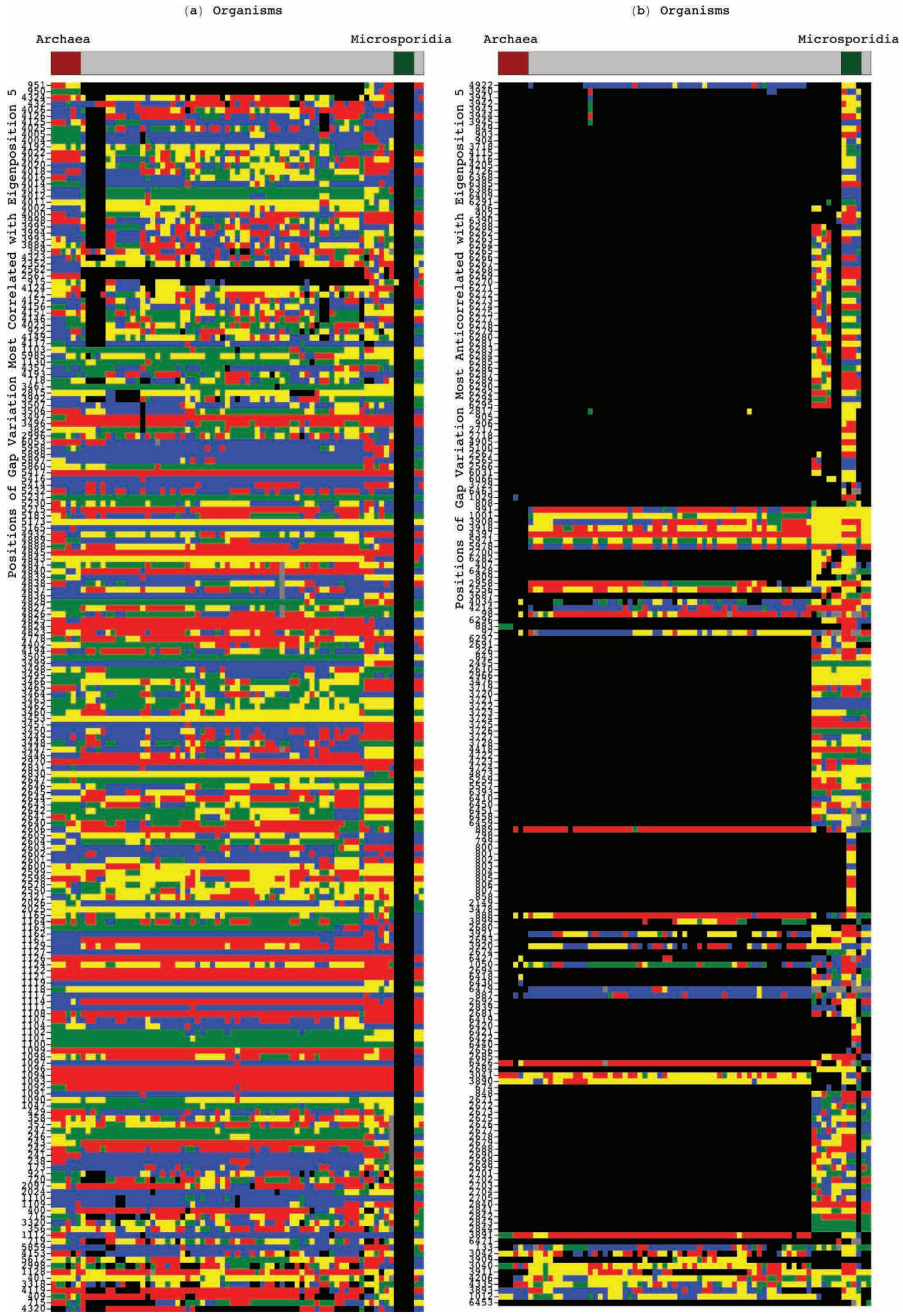

Figure S16. Sequence gaps exclusive to Microsporidia vs. Archaea 23S rRNAs. Raster displays of the 200 and 199 positions in the 23S alignment for which the A nucleotide frequency variation is most correlated or anticorrelated with the fifth 23S eigenposition, as identified by the gap segment of the fifth 23S eigenorganism. The color bars highlight the taxonomic groups that are differentiated by the fifth eigenposition and eigenorganism, i.e., the Archaea and the Microsporidia. (*a*) The 200 correlated positions include all 59 unpaired A nucleotides exclusively conserved in the Eukarya excluding the Microsporidia. (*b*) The 199 anticorrelated positions include all 41 unpaired A nucleotides exclusively conserved in the Bacteria.

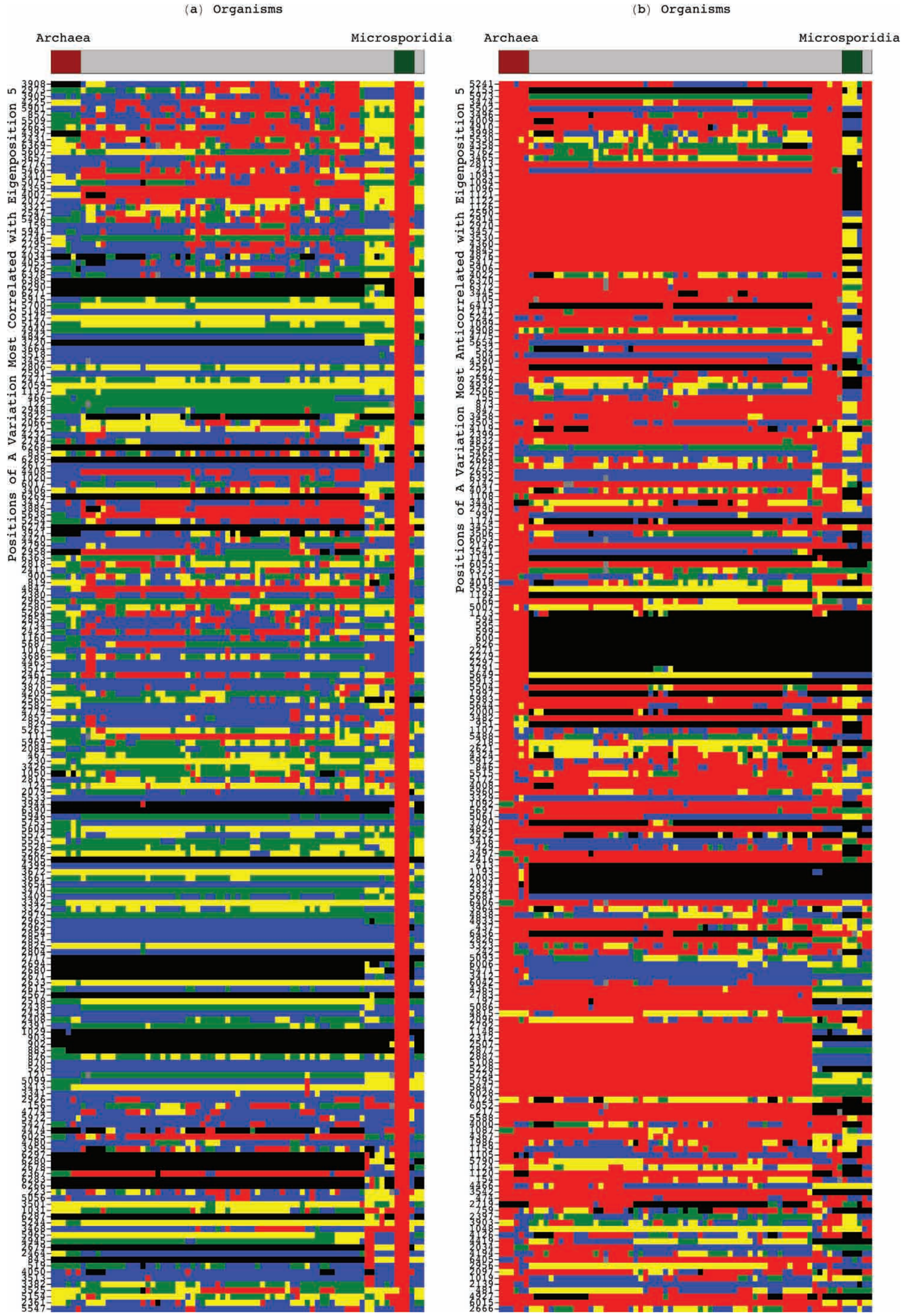

Figure S17. Adenosines exclusive to Microsporidia vs. Archaea 23S rRNAs. Raster displays of the 200 positions in the 23S alignment for which the A nucleotide frequency variation is most correlated or anticorrelated with the fifth 23S eigenposition, as identified by the A nucleotide segment of the fifth 23S eigenorganism. The color bars highlight the Archaea and the Microsporidia. (*a*) The 200 correlated positions include 16 of the 31 unpaired A nucleotides exclusively conserved in the Microsporidia. (*b*) The 200 anticorrelated positions include 39 of the 49 unpaired A nucleotides exclusively conserved in the Archaea.
