# Supplementary material for: Tensor Decomposition Reveals Concurrent Evolutionary Convergences and Divergences and Correlations with Structural Motifs in Ribosomal RNA
Source: PLoS One. 2011 Apr 29;6(4):e18768. doi: 10.1371/journal.pone.0018768 (PMC3094155; doi:10.1371/journal.pone.0018768)

(* © Andrew M. Gross and Orly Alter 2011, All Rights Reserved *)

```
(* Mode-1 HOSVD of 16S rRNA Sequence Alignment from 339 Organisms *)

(* Initialize *)

Clear["Global`*"]
organismAnnotations = 2;
positionAnnotations = 1;

(* Define Path to Datasets *)

path = "Desktop/rRNA/Data/";

(* Read Alignment *)

stream = path <> "Dataset_S2.txt";
matrix = Import[stream, "Table"];

alignment = Transpose[Drop[ matrix, organismAnnotations, positionAnnotations]];
positionNames = Drop[matrix[[All, positionAnnotations]], organismAnnotations];
organismNames = Drop[matrix[[organismAnnotations]], positionAnnotations];
{organisms, positions} = Dimensions[alignment];
Clear[stream, matrix];

(* Compute Mode-1 HOSVD *)

(* Convert Alignment to Third-Order Tensor *)

{vecA, vecC, vecG, vecU, vecN, vecGap} = DiagonalMatrix[Table[1.0, {6}]];
data16S = ReplaceAll[alignment,
   {"A" → vecA, "C" → vecC, "G" → vecG, "U" → vecU, "N" → vecN, "-" → vecGap}];
data16S = Flatten[ Transpose[data16S, {3, 1, 2}], 1];

{eigenorganisms, eigenexpressions, eigenpositions} =
  SingularValueDecomposition[data16S, Min[Dimensions[data16S]]];
eigenexpressions = Diagonal[eigenexpressions];
eigenexpressions = Select[eigenexpressions, # > 0 &];
eigenpositions = Transpose[eigenpositions];
eigenpositions[[4]] = -eigenpositions[[4]];
eigenorganisms[[All, 4]] = -eigenorganisms[[All, 4]];
eigenpositions[[7]] = -eigenpositions[[7]];
eigenorganisms[[All, 7]] = -eigenorganisms[[All, 7]];
degrees = Dimensions[eigenexpressions]〚1〛;
fractions = eigenexpressions^2 / Sum[eigenexpressions〚a〛^2, {a, 1, degrees}];
entropy = -N[Sum[fractions〚a〛 Log[fractions〚a〛], {a, 1, degrees}] / Log[degrees]];
entropy = N[ Round[100 entropy] / 100]

0.28
```

```
(* Read Taxonomy Annotations *)

stream = path <> "Dataset_S1.txt";
matrix = Import[stream, "Table"];
taxonomy = Transpose[Drop[matrix, 1, 3]];
Clear[stream, matrix];

(* Define Function to Find All Positions of a Group *)

parse[group_] := {
  regX = StringSplit[group, " + "];
  If[Dimensions[regX][[1]] > 1, {
    groupPositions = {},
    Do[{
      level = Min[Position[taxonomy, regX[[i]]][[All, 1]]],
      groupPositions = Union[groupPositions, Flatten[Position[taxonomy[[level]], regX[[i]]]]]
     }, {i, 1, Dimensions[regX][[1]]}]},
   {regX = StringSplit[group, " - "],
    If[Dimensions[regX][[1]] > 1, {
      level = Min[Position[taxonomy, regX[[1]]][[All, 1]]],
      groupPositions = Flatten[Position[taxonomy[[level]], regX[[1]]]],
      Do[{
        level = Min[Position[taxonomy, regX[[i]]][[All, 1]]],
        groupPositions =
         Complement[groupPositions, Flatten[Position[taxonomy[[level]], regX[[i]]]]]
       }, {i, 2, Dimensions[regX][[1]]}]},
     {level = Min[Position[taxonomy, group][[All, 1]]],
      groupPositions = Flatten[Position[taxonomy[[level]], group]]
     }]}];
  groupPositions}

(* Calculate Enrichment of Significant Eigenpositions in Taxonomic Groups *)

(* Define Cutoffs *)

groupSize = 75;
pValueCutoff = .05;

(* Count Taxonomy Annotations *)

taxonomicGroups = Table[Union[taxonomy[[i]]], {i, 1, 8}];
countTable = Table[{taxonomicGroups[[a, b]], Table[0, {6}, {3}]},
   {a, 1, Dimensions[taxonomicGroups][[1]]}, {b, 1, Dimensions[taxonomicGroups[[a]]][[1]]}];
```

```
Do[{
   annotations = taxonomy[[i]];
   stages = taxonomicGroups[[i]];
   numbers =
    Flatten[Table[{Count[annotations, stages[[a]]]}, {a, 1, Dimensions[stages][[1]]}]];
   annotations = Table[{annotations[[a]]}, {a, 1, organisms}];
   Do[{
     eigenposition = Table[{eigenpositions[[f, a]]}, {a, 1, organisms}],
     pattern = Sort[Join[eigenposition, annotations, 2], OrderedQ[{#2, #1}] &][[All, 2]],
     Do[
      countTable[[i, a, 2, f - 1]] = {
        numbers[[a]],
        Count[pattern[[1 ;; groupSize]], stages[[a]]],
        Count[pattern[[organisms - groupSize + 1 ;; organisms]], stages[[a]]]},
      {a, 1, Dimensions[stages][[1]]}]},
    {f, 2, 7}]},
  {i, 1, 5}];

flatCountTable = Flatten[countTable, 1];
getNumbers[annotation_, eigenposition_] :=
 flatCountTable[[Position[flatCountTable[[All, 1]], annotation][[1, 1]]]][[2, eigenposition - 1]]

(* Compute Significance of Eigenposition Association with Taxonomic Groups *)

getEnrichment[group_, eigenposition_, tail_] := {
  regX = StringSplit[group, " + "];
  If[Dimensions[regX][[1]] > 1,
   nums = Sum[getNumbers[regX[[a]], eigenposition], {a, 1, Dimensions[regX][[1]]}],
   {regX = StringSplit[group, " - "],
    If[Dimensions[regX][[1]] > 1,
     nums = getNumbers[regX[[1]], eigenposition] -
       Sum[getNumbers[regX[[a]], eigenposition], {a, 2, Dimensions[regX][[1]]}],
     nums = getNumbers[group, eigenposition]]}];
  If[tail == "Correlated", tailGroup = 2, tailGroup = 3];
  pValue = Sum[N[PDF[HypergeometricDistribution[groupSize, nums[[1]], organisms], k]],
    {k, nums[[tailGroup]], groupSize}];
  {eigenposition, group, nums[[tailGroup]], nums[[1]], ScientificForm[pValue, 2]}}

corrTable =
  acorrTable =
   {{"", "Taxonomic Group", "n", "N", "P-value"}};
corrList = {
   {2, "Eukarya - Microsporidia"},
   {4, "Gamma_Subdivision"},
   {5, "Microsporidia"},
   {6, "Fungi/Metazoa - Microsporidia"},
   {7, "Alveolata"}};
acorrList = {
   {2, "Bacteria"},
   {3, "Archaea + Microsporidia"},
   {4, "Actinobacteria + Archaea"},
   {5, "Archaea"},
   {6, "Rhodophyta"},
   {7, "Fungi/Metazoa - Microsporidia"}};
```

```
Do[
 corrTable = Append[
   corrTable,
   getEnrichment[corrList[[a, 2]],
     corrList[[a, 1]],
      "Correlated"][[1]]],
 {a, 1, Dimensions[corrList][[1]]}]
corrTable = ReplaceAll[corrTable, "Gamma_Subdivision" -> "Gamma Proteobacteria"];
corrTable = ReplaceAll[corrTable, "Fungi/Metazoa - Microsporidia" →
    "Fungi/Metazoa -\n Microsporidia"];
corrTable = Insert[corrTable, {"3", "", SpanFromLeft, SpanFromLeft, SpanFromLeft}, 3];
Do[
 acorrTable = Append[
   acorrTable,
   getEnrichment[acorrList[[a, 2]],
     acorrList[[a, 1]],
      "Anticorrelated"][[1]]],
 {a, 1, Dimensions[acorrList][[1]]}]
acorrTable = ReplaceAll[acorrTable, "Actinobacteria + Archaea" →
    "Actinobacteria +\n Archaea"];
acorrTable = ReplaceAll[acorrTable, "Fungi/Metazoa - Microsporidia" →
    "Fungi/Metazoa -\n Microsporidia"];
xHeadings = {{
    "", "Correlated", SpanFromLeft, SpanFromLeft,
    SpanFromLeft, "Anticorrelated", SpanFromLeft, SpanFromLeft, SpanFromLeft}};
yHeadings = Transpose[
   {{"", "", Rotate["Eigenposition", Pi / 2], SpanFromAbove,
     SpanFromAbove, SpanFromAbove, SpanFromAbove, SpanFromAbove}}];
Grid[Join[yHeadings, Join[xHeadings, Join[corrTable, acorrTable[[All, 2 ;; 5]], 2]], 2],
 Alignment → {Left, Bottom}, Spacings → {Scaled[.01], Scaled[.02]}, Frame → All]
```

		Correlated				Anticorrelated			
		Taxonomic Group	n	N	P-value	Taxonomic Group	n	N	P-value
Eigenposition	2	Eukarya - Microsporidia	75	107	5.7×10^{-50}	Bacteria	75	175	1.5×10^{-26}
	3					Archaea + Microsporidia	57	57	3.4×10^{-49}
	4	Gamma Proteobacteria	32	32	$2. \times 10^{-24}$	Actinobacteria + Archaea	72	74	2.5×10^{-67}
	5	Microsporidia	29	36	1.8×10^{-15}	Archaea	21	21	1.5×10^{-15}
	6	Fungi/Metazoa - Microsporidia	32	32	$2. \times 10^{-24}$	Rhodophyta	26	26	1.8×10^{-19}
	7	Alveolata	21	21	1.5×10^{-15}	Fungi/Metazoa - Microsporidia	32	32	$2. \times 10^{-24}$

```
(* Calculate Enrichment of Motifs Conserved in Taxonomic Groups *)

(* Read Base Pairing *)

stream = path <> "Dataset_S3.txt";
matrix = Import[stream, "Table"];
pairing = Transpose[Drop[matrix, organismAnnotations, positionAnnotations]];
Clear[stream, matrix];

(* Fold Eigenorganisms into a Third-Order Tensor *)

eigenorganismsTensor = Table[eigenorganisms[[Range[a, positions * 6, 6], All]], {a, 1, 6}];
Dimensions[eigenorganismsTensor]

{6, 3249, 339}

(* Define Cutoffs *)

conservationValue1 = 80;
conservationValue2 = 20;
conservationValue3 = 60;
groupSize = 100;
nearZero = .00001;
nucleotides = {"A", "C", "G", "U", "N", "-"};
motifs = {"unpairedA", "unpairedC", "unpairedG", "unpairedU", "unpairedN",
   "helix", "pairedA", "pairedC", "pairedG", "pairedU", "pairedN", "nucGap"};
stages = {"Group", "All"};
allPositions = Range[1, organisms];

(* Calculate Nucleotide and Basepair Frequencies for All Organisms *)

nucleotideCount = Table[Count[alignment[[All, i]], nucleotides[[j]]],
   {i, 1, positions}, {j, 1, Dimensions[nucleotides][[1]]}];
sequenceLength = organisms - nucleotideCount[[All, 6]];
bpCount =
  Table[{Count[pairing[[All, i]], "Y"], Count[pairing[[All, i]], "N"]}, {i, 1, positions}];

ntFrequncyAll = N[(nucleotideCount / organisms) * 100];
bpFrequencyAll = N[(bpCount / sequenceLength) * 100];

(* Calculate Correlation of First Eigenposition and Eigenorganisms with Frequency *)

ones = Table[1, {a, 1, organisms}];
eigenpositions[[1]].(ones / Sqrt[ones.ones])

-0.996439

frequencies = Flatten[ntFrequncyAll];
eigenorganisms[[All, 1]].(frequencies / Sqrt[frequencies.frequencies])

-0.999444
```

```
(* Parse Datasets to Include Only Organisms of a Given Group *)

parseAll[group_] := {
  groupPositions = parse[group][[1]];
  restPositions = Complement[allPositions, groupPositions];
  groupAlignment = alignment[[groupPositions]];
  groupBasePairing = pairing[[groupPositions]];
  restAlignment = alignment[[restPositions]];
  restBasePairing = pairing[[restPositions]];
  groupLength = Dimensions[groupPositions][[1]];
  restLength = Dimensions[restPositions][[1]];
  groupNucleotideCount = Table[Count[groupAlignment[[All, i]], nucleotides[[j]]],
    {i, 1, positions}, {j, 1, Dimensions[nucleotides][[1]]}];
  groupLengths = groupLength - groupNucleotideCount[[All, 6]];
  restLengths = sequenceLength - groupLengths;
  restCount = nucleotideCount - groupNucleotideCount;
  groupLengths = Replace[groupLengths, 0 → nearZero, {1}];
  restLengths = Replace[restLengths, 0 → nearZero, {1}];
  ntFrequencyGroup = N[(groupNucleotideCount / groupLength) * 100];
  ntFrequencyRest = N[(restCount / restLength) * 100];
 }

(* Calculate Basepair Frequencies *)

bpFrequencies[] := {
  bpCountGroup = Table[{Count[groupBasePairing[[All, i]], "Y"],
     Count[groupBasePairing[[All, i]], "N"]}, {i, 1, positions}];
  bpCountRest = bpCount - bpCountGroup;
  bpFrequencyGroup = N[(bpCountGroup / groupLengths) * 100];
  bpFrequencyRest = N[(bpCountRest / restLengths) * 100];
 }

(* Calculate Basepair Annotations *)

bpAnnotations[seg_] := {
  bpFrequencies[];
  segment = If[seg > 6, seg - 6, seg];
  paired = unpaired = nuc = Table["-", {positions}];
  Do[{
    If[(ntFrequncyAll[[i, segment]] > conservationValue1),
     {nuc[[i]] = "All",
      If[bpFrequencyAll[[i, 1]] > bpFrequencyAll[[i, 2]],
       paired[[i]] = "All", unpaired[[i]] = "All"]}],
    If[((ntFrequencyGroup[[i, segment]] > conservationValue1) &&
       (ntFrequencyRest[[i, segment]] < conservationValue2)),
     {nuc[[i]] = "Group",
      If[(bpFrequencyGroup[[i, 1]] > bpFrequencyGroup[[i, 2]]),
       paired[[i]] = "Group", unpaired[[i]] = "Group"]}]},
   {i, 1, positions}],
  Switch[segment,
    1, {pairedA = paired, unpairedA = unpaired, nucA = nuc},
    2, {pairedC = paired, unpairedC = unpaired, nucC = nuc},
    3, {pairedG = paired, unpairedG = unpaired, nucG = nuc},
    4, {pairedU = paired, unpairedU = unpaired, nucU = nuc},
    5, {pairedN = paired, unpairedN = unpaired, nucN = nuc},
    6, {pairedGap = paired, unpairedGap = unpaired, nucGap = nuc}];
 }
```

```
(* Calculate Structure Annotations *)

structAnnotations[] := {
  bpFrequencies[];
  helix = Table["-", {positions}];
  annotation = "Group";
  Do[If[(bpFrequencyGroup[[i, 1]] > conservationValue3 &&
      bpFrequencyGroup[[i, 1]] > bpFrequencyRest[[i, 1]]), helix[[i]] = annotation],
   {i, 1, positions}]
 }

(* Get Enrichment of Motifs Conserved in Taxonomic Groups *)

getEnrichments[group_, tail_, eigenorganism_, motif_, segment_, groupSize_] := {
  segmentNumber = Position[nucleotides, segment][[1, 1]];
  motifNumber = Position[motifs, motif][[1, 1]];
  parseAll[group];
  If[motif == "helix", structAnnotations[], bpAnnotations[motifNumber]];
  annotations = Switch[motifNumber, 1, unpairedA, 2, unpairedC, 3, unpairedG, 4, unpairedU, 5,
    pairedN, 6, helix, 7, pairedA, 8, pairedC, 9, pairedG, 10, pairedU, 11, pairedN, 12, nucGap];
  numbers = Flatten[Table[{Count[annotations, stages[[a]]]}, {a, 1, Dimensions[stages][[1]]}]];
  annotations = Table[{annotations[[a]]}, {a, 1, positions}];
  pattern =
   Sort[Join[eigenorganismsTensor[[segmentNumber, All, {eigenorganism}]], annotations, 2],
     OrderedQ[{#2, #1}] &][[All, 2]];
  countTable = Table[{
     numbers[[a]],
     Count[Flatten[pattern[[1 ;; groupSize]]], stages[[a]]],
     Count[Flatten[pattern[[positions - groupSize + 1 ;; positions]]], stages[[a]]]},
    {a, 1, Dimensions[stages][[1]]}];
  counts = countTable[[1]];
  If[tail == "Correlated", tailGroup = 2, tailGroup = 3];
  pValue = Sum[N[PDF[HypergeometricDistribution[groupSize, counts[[1]], positions], k]],
    {k, counts[[tailGroup]], groupSize}];
  result = {counts[[tailGroup]], counts[[1]], ScientificForm[pValue, 2]}
 }

(* Create Formatted Table of Selected Enrichments *)

addTableRow[eigenorganism_, isEigFirst_, segment_, isSegmentFirst_, motif_, isMotifFirst_,
  isCorr_, corrGroup_, isCorrFirst_, isAnticorr_, acorrGroup_, isAcorrFirst_,
  groupSizeCorr_, groupSizeAcorr_] := {head = {If[isEigFirst, eigenorganism, SpanFromAbove],
    If[isSegmentFirst, segment, SpanFromAbove], If[isMotifFirst, motif, SpanFromAbove]};
  corrSection = If[isCorr, Join[If[isCorrFirst, {corrGroup}, {SpanFromAbove}], getEnrichments[
       corrGroup, "Correlated", eigenorganism, motif, segment, groupSizeCorr][[1]]],
    If[isCorrFirst, {"", SpanFromLeft, SpanFromLeft, SpanFromLeft},
     {SpanFromAbove, SpanFromBoth, SpanFromBoth, SpanFromBoth}]];
  acorrSection = If[isAnticorr,
    Join[If[isAcorrFirst, {acorrGroup}, {SpanFromAbove}], getEnrichments[acorrGroup,
       "Anticorrelated", eigenorganism, motif, segment, groupSizeAcorr][[1]]],
    If[isAcorrFirst, {"", SpanFromLeft, SpanFromLeft, SpanFromLeft},
     {SpanFromAbove, SpanFromBoth, SpanFromBoth, SpanFromBoth}]];
  result = Join[head, corrSection, acorrSection];
  result
 }
```

```
addTableRow[eigenorganism_, isEigFirst_, segment_, isSegmentFirst_, motif_, isMotifFirst_,
  isCorr_, corrGroup_, isCorrFirst_, isAnticorr_, acorrGroup_, isAcorrFirst_] :=
 addTableRow[eigenorganism, isEigFirst, segment, isSegmentFirst, motif, isMotifFirst,
  isCorr, corrGroup, isCorrFirst, isAnticorr, acorrGroup, isAcorrFirst, groupSize, groupSize]

enrichmentTable =
  Join[
   {{"", "", "", "Correlated", SpanFromLeft, SpanFromLeft,
     SpanFromLeft, "Anticorrelated", SpanFromLeft, SpanFromLeft, SpanFromLeft}},
   {{"  ", "Tensor \n Slice", "Structure \n Motif", "Taxonomic \n Group",
     "n", "N", "P-value", "Taxonomic \n Group", "n", "N", "P-value"}},
   addTableRow[2, True, "A", True, "unpairedA", True, True,
    "Eukarya - Microsporidia", True, True, "Bacteria", True],
   addTableRow[2, False, "-", True, "nucGap", True, True,
    "Eukarya - Microsporidia", False, True, "Bacteria", False, 124, 100],
   addTableRow[2, False, "-", False, "unpairedA", True, True,
    "Bacteria", True, False, Null, True, 124, 100],
   addTableRow[3, True, "C", True, "helix", True, False, Null,
    True, True, "Archaea + Microsporidia", True],
   addTableRow[3, False, "G", True, "helix", False, False, Null,
    False, True, "Archaea + Microsporidia", False],
   addTableRow[3, False, "U", True, "helix", False, False, Null,
    False, True, "Archaea + Microsporidia", False],
   addTableRow[3, False, "-", True, "nucGap", True, False, Null,
    False, True, "Archaea + Microsporidia", False],
   addTableRow[4, True, "A", True, "unpairedA", True, True,
    "Gamma_Subdivision", True, False, Null, True],
   addTableRow[4, False, "-", True, "helix", True, True,
    "Actinobacteria + Archaea", True, False, Null, False],
   addTableRow[5, True, "A", True, "unpairedA", True, False, Null, True, True, "Archaea", True],
   addTableRow[5, False, "G", True, "helix", True, False, Null, False, True, "Archaea", False],
   addTableRow[5, False, "C", True, "helix",
    False, True, "Microsporidia", True, True, "Archaea", False],
   addTableRow[5, False, "U", True, "helix", False, True,
    "Microsporidia", False, True, "Archaea", False],
   addTableRow[5, False, "-", True, "unpairedA", True, True, "Archaea", True, False, Null, True],
   addTableRow[6, True, "A", True, "unpairedA", False,
    True, "Fungi/Metazoa - Microsporidia", True, True, "Rhodophyta", True],
   addTableRow[7, True, "A", False, "unpairedA", False, True, "Alveolata",
    True, True, "Fungi/Metazoa - Microsporidia", True]
  ];

yHeadings = Transpose[{Join[{""}, {Rotate["Eigenorganism", Pi / 2]},
     Table[SpanFromAbove, {Dimensions[enrichmentTable][[1]] - 2}]]}];

enrichmentTable = ReplaceAll[enrichmentTable, "-" → "Gap"];
enrichmentTable = ReplaceAll[enrichmentTable, "unpairedA" → "Unpaired A"];
enrichmentTable = ReplaceAll[enrichmentTable, "nucGap" → "Gap"];
enrichmentTable = ReplaceAll[enrichmentTable, "helix" → "Helix"];
enrichmentTable =
  ReplaceAll[enrichmentTable, "Eukarya - Microsporidia" → "Eukarya -\n Microsporidia"];
enrichmentTable = ReplaceAll[enrichmentTable,
   "Archaea + Microsporidia" → "Archaea +\n Microsporidia"];
enrichmentTable = ReplaceAll[enrichmentTable, "Gamma_Subdivision" -> "Gamma\n Proteobacteria"];
enrichmentTable = ReplaceAll[enrichmentTable, "Actinobacteria + Archaea" →
    "Actinobacteria +\n Archaea"];
enrichmentTable = ReplaceAll[enrichmentTable, "Fungi/Metazoa - Microsporidia" →
    "Fungi/Metazoa -\n Microsporidia"];
```

```
Grid[Join[yHeadings, enrichmentTable, 2],
 Alignment → {Left, Bottom}, Spacings → {Scaled[.01], Scaled[.02]}, Frame → All ]
```

				Correlated				Anticorrelated			
		Tensor Slice	Structure Motif	Taxonomic Group	n	N	P-value	Taxonomic Group	n	N	P-value
Eigenorganism	2	A	Unpaired A	Eukarya − Microsporidia	48	66	2.3×10^{-63}	Bacteria	50	50	1.2×10^{-82}
		Gap	Gap		124	211	$4. \times 10^{-167}$		57	58	9.8×10^{-94}
			Unpaired A	Bacteria	13	50	2.1×10^{-8}				
	3	C	Helix					Archaea + Microsporidia	76	1148	$4. \times 10^{-17}$
		G							68	1148	1.5×10^{-11}
		U							65	1148	8.5×10^{-10}
		Gap	Gap						6	6	7.3×10^{-10}
	4	A	Unpaired A	Gamma Proteobacteria	11	11	1.4×10^{-17}				
		Gap	Helix	Actinobacteria + Archaea	34	153	8.6×10^{-22}				
	5	A	Unpaired A					Archaea	14	14	2.7×10^{-22}
		G	Helix						84	933	1.8×10^{-31}
		C		Microsporidia	58	947	9.6×10^{-10}		85	933	1.3×10^{-32}
		U			55	947	3.6×10^{-8}		57	933	1.8×10^{-9}
		Gap	Unpaired A	Archaea	7	14	6.1×10^{-8}				
	6	A		Fungi/Metazoa − Microsporidia	9	16	1.7×10^{-10}	Rhodophyta	25	27	2.2×10^{-37}
	7			Alveolata	25	31	4.3×10^{-34}	Fungi/Metazoa − Microsporidia	10	16	3.3×10^{-12}

```
(* Visualize Mode-1 HOSVD *)

(* Define Display Parameters *)

nucleotides = {"D_A", "D_C", "D_G", "D_U", "D_N", "D_-"};
uslice = {"U_A", "U_C", "U_G", "U_U", "U_N", "U_-"};
x = 195;
y = -130;
d = 117;
i = 0;
j = 0;
xlabelLevel = 797;
matrixLabelLevel = 900;

(* Separate Alignment by Nucleotide *)

{vecA, vecC, vecG, vecU, vecN, vecGap} = DiagonalMatrix[Table[1, {6}]];
binaryAlignmentTensor = ReplaceAll[alignment,
   {"A" → vecA, "C" → vecC, "G" → vecG, "U" → vecU, "N" → vecN, "-" → vecGap}];
binaryAlignmentTensor = Transpose[binaryAlignmentTensor, {2, 3, 1}];

(* Create Set Rasters for 𝒟 Tensor *)

tensorSlicePosition[segment_] :=
 {{(7 - segment) y + i, (segment - 1) (x - d) + j}, {(6 - segment) y + i, (segment + 1) (x - d) + 2 d + j}}

dataTensor = {
   Graphics[{
     Table[Raster[Reverse[binaryAlignmentTensor[[a]]], tensorSlicePosition[a],
       ColorFunction → (Switch[#, 1, Red, 0, Black] &)], {a, 1, 6}],
     Dashed, Line[{{y + i, 7 x - 5 d + j}, {6 y + i, 2 x + j}}],
     Line[{{0 + i, 5 (x - d) + j}, {5 y + i, 0 + j}}],
     White, Line[{{0 + i, 7 x - 5 d + j}, {5 y + i, 2 x + j}}]}],
   Graphics[Text["Positions", {6 y - 20, 324}, {0, 0}, {0, 1}]],
   Graphics[Text["Organisms", {5.5 y + 650, 871}, {0, 0}]],
   Graphics[Text["Nucleotides", {3.5 y, 5 (x - d) + j + 1.4 x}, {1, -1}, {1, .6}]],
   Table[Graphics[
     Line[{{a * y + y, (5 - a) (x - d) + j + 2 x}, {a * y - 20 + y, (5 - a) (x - d) + j + 17 + 2 x}}]], {a, 0, 5}],
   Table[Graphics[Text[StyleForm[nucleotides[[6 - a]], FontSize → 15],
      {(a + 1) * y - 40, (5 - a) (x - d) + j + 30 + 2 x}, {0, 0}]], {a, 0, 5}],
   Graphics[Text[Style["𝒟", 20], {.5 y + i, matrixLabelLevel}, {0, 0}]]
  };

(* Create Set Rasters for 𝒰 Tensor *)

contrast = 150;
uRasters = Table[
   adjustedValue = eigenorganismsTensor[[c, b, a]] * contrast;
   If[adjustedValue > 0,
    {adjustedValue, 0, 0},
    {0, -adjustedValue, 0}],
   {c, 1, 6}, {a, 1, organisms}, {b, 1, positions}];
```

```
i = 870;
uTensor = {
   Graphics[{
     Table[Raster[Reverse[uRasters[[a]]],
       tensorSlicePosition[a], ColorFunction → RGBColor], {a, 1, 6}],
     Dashed, Line[{{y + i, 7 x - 5 d + j}, {6 y + i, 2 x + j}}],
     Line[{{0 + i, 5 (x - d) + j}, {5 y + i, 0 + j}}],
     White, Line[{{0 + i, 7 x - 5 d + j}, {5 y + i, 2 x + j}}]}],
   Graphics[Text["Positions", {6 y - 20 + i, 324 + j}, {0, 0}, {0, 1}]],
   Graphics[Text["Eigenorganisms", {5.5 y + 650 + i, j + xlabelLevel}, {0, 0}]],
   Graphics[Text["Nucleotides", {3.5 y + i, 5 (x - d) + j + 1.4 x}, {1, -1}, {1, .6}]],
   Table[Graphics[Line[
      {{a * y + y + i, (5 - a) (x - d) + j + 2 x}, {a * y - 20 + y + i, (5 - a) (x - d) + j + 17 + 2 x}}]], {a, 0, 5}],
   Table[Graphics[Text[StyleForm[uslice[[6 - a]], FontSize → 15],
      {(a + 1) * y - 40 + i, (5 - a) (x - d) + j + 30 + 2 x}, {0, 0}]], {a, 0, 5}],
   Graphics[Text[Style["𝒰", 20], {.5 y + i, matrixLabelLevel}, {0, 0}]]
  };

(* Create Σ Raster *)

expressionRaster =
  Table[{0, 0, 0}, {Dimensions[eigenexpressions][[1]]}, {Dimensions[eigenexpressions][[1]]}];
Do[expressionRaster[[a, a]] = {Sqrt[eigenexpressions[[a]] / eigenexpressions[[1]]], 0, 0},
  {a, 1, Dimensions[eigenexpressions][[1]]}];

i = i - 8;
eigenValues = {
   Graphics[Raster[Reverse[expressionRaster[[1 ;; 25, 1 ;; 25]]],
     {{180 + y + i, 7 x - 5 d + y + j}, {180 + i, 7 x - 5 d + j}}]],
   Graphics[Text[StyleForm["Σ", FontSize → 20], {180 + .5 y + i, matrixLabelLevel}, {0, 0}]],
   Graphics[Text["Eigenorganisms", {30 + i, j + 324 + j + 5 (x - d)}, {0, 0}, {0, 1}]],
   Graphics[Text["Eigenpositions", {115 + i, j + xlabelLevel}, {0, 0}]]
  };

(* Create V^T Raster *)

contrast = 25;
vTransposeRaster = Table[If[eigenpositions[[a, b]] > 0,
     {eigenpositions[[a, b]] * contrast, 0, 0}, {0, -eigenpositions[[a, b]] * contrast, 0}],
    {a, 1, organisms}, {b, 1, organisms}];

i = i - 8;
vTranspose = {
   Graphics[
    Raster[Reverse[vTransposeRaster], {{360 + y + i, j + 7 x - 5 d + y}, {360 + i, j + 7 x - 5 d}}]],
   Graphics[Text["Eigenpositions", {210 + i, 324 + j + 5 (x - d)}, {0, 0}, {0, 1}]],
   Graphics[Text["Organisms", {293 + i, 871}, {0, 0}]],
   Graphics[Text[StyleForm["V^T", FontSize → 20], {360 + .5 y + i, matrixLabelLevel}, {0, 0}]]
  };

(* Create Taxonomic Color Bars and Hierarchical Tree *)

barHeight = 12;
yOffset = xlabelLevel - barHeight - 6;
xOffset1 = y;
xOffset2 = 360 + y + i;
```

```
bar[group_, level_, red_, green_, blue_] := {
  Graphics[{RGBColor[red, green, blue], EdgeForm[Thickness[.0002]],
    Rectangle[{(Max[parse[group]] + 1) * N[(-y / organisms)] + xOffset1, level * barHeight + yOffset},
     {Min[parse[group]] * N[(-y / organisms)] + xOffset1, barHeight + level * barHeight + yOffset}],
    Rectangle[{(Max[parse[group]] + 1) * N[(-y / organisms)] + xOffset2, level * barHeight + yOffset},
     {Min[parse[group]] * N[(-y / organisms)] + xOffset2,
      barHeight + level * barHeight + yOffset}]}],
  If[level == 1, lineUp[group, 20, 35],
   lineUp[group, 5, 20]]}

lineUp[group_, startHeight_, endHeight_] := {
  pos = ( Min[parse[group]] + Max[parse[group]] + 1) * (.5 * N[(-y / organisms)]);
  Graphics[Line[{{pos + xOffset1, startHeight + barHeight * 3 + yOffset},
     {pos + xOffset1, endHeight + barHeight * 3 + yOffset}}]],
  Graphics[Line[{{pos + xOffset2, startHeight + barHeight * 3 + yOffset},
     {pos + xOffset2, endHeight + barHeight * 3 + yOffset}}]]}

connect[group1_, group2_, height_] := {
  pos1 = ( Min[parse[group1]] + Max[parse[group1]] + 1) * (.5 * N[(-y / organisms)]);
  pos2 = ( Min[parse[group2]] + Max[parse[group2]] + 1) * (.5 * N[(-y / organisms)]);
  Graphics[Line[{{pos1 + xOffset1, height + barHeight * 3 + yOffset},
     {pos2 + xOffset1, height + barHeight * 3 + yOffset}}]],
  Graphics[Line[{{pos1 + xOffset2, height + barHeight * 3 + yOffset},
     {pos2 + xOffset2, height + barHeight * 3 + yOffset}}]]}

domain = {
   bar["Archaea", 1, .6, 0, 0],
   bar["Bacteria", 1, 0, 0, .6],
   bar["Eukarya", 1, 0, .6, 0]};

subgroupings1 = {
   bar["Crenarchaeota", 2, 1, .4, 0],
   bar["Euryarchaeota", 2, 1, 0, .1],
   bar["Proteobacteria", 2, .2, .15, .25],
   bar["Actinobacteria", 2, .1, .2, .3],
   bar["Firmicutes", 2, .1, .4, .5],
   bar["Tenericutes", 2, .3, .9, 1],
   bar["Planctomycetes", 2, 0, 0, .4],
   bar["Spirochaetes", 2, 0, .2, .6],
   bar["Thermotogae", 2, .6, .8, 1],
   bar["Deinococci", 2, .2, 0, .8],
   bar["Chlamydiae", 2, 0, .7, 1],
   bar["Cyanobacteria", 2, 0, 0, .4],
   bar["Other_Bacteria", 2, 0, .1, .7],
   bar["Other_Eukarya", 2, 0, .9, 0],
   bar["Alveolata", 2, .5, 1, .1],
   bar["Fungi/Metazoa", 2, .2, 1, .5],
   bar["Rhodophyta", 2, .2, .7, .3],
   bar["Viridiplantae", 2, .1, .6, .5],
   bar["Stramenopiles", 2, .6, .8, .1]};

connections = {
   connect["Crenarchaeota", "Euryarchaeota", 20],
   connect["Proteobacteria", "Other_Bacteria", 20],
   connect["Other_Eukarya", "Stramenopiles", 20],
   connect["Archaea", "Eukarya", 35]
  };
```

```
(* Show Visualization of 𝒟 = 𝒰ΣV^T *)

f1 = Show[{domain, subgroupings1, connections, dataTensor, uTensor, eigenValues,
    vTranspose, Graphics[Text[StyleForm["=", FontSize → 40], {0, -.3 x + 250}, {-1, 0}]]},
   BaseStyle → { FontSize →  10, FontFamily → "Courier"},  ImageSize → 1450];

Clear[eigenorganismsTensor, dataTensor, uRasters, uTensor, vTransposeRaster,
  vTranspose, expressionRaster, eigenValues, domain, subgroupings1, connections]

Show[f1]
```

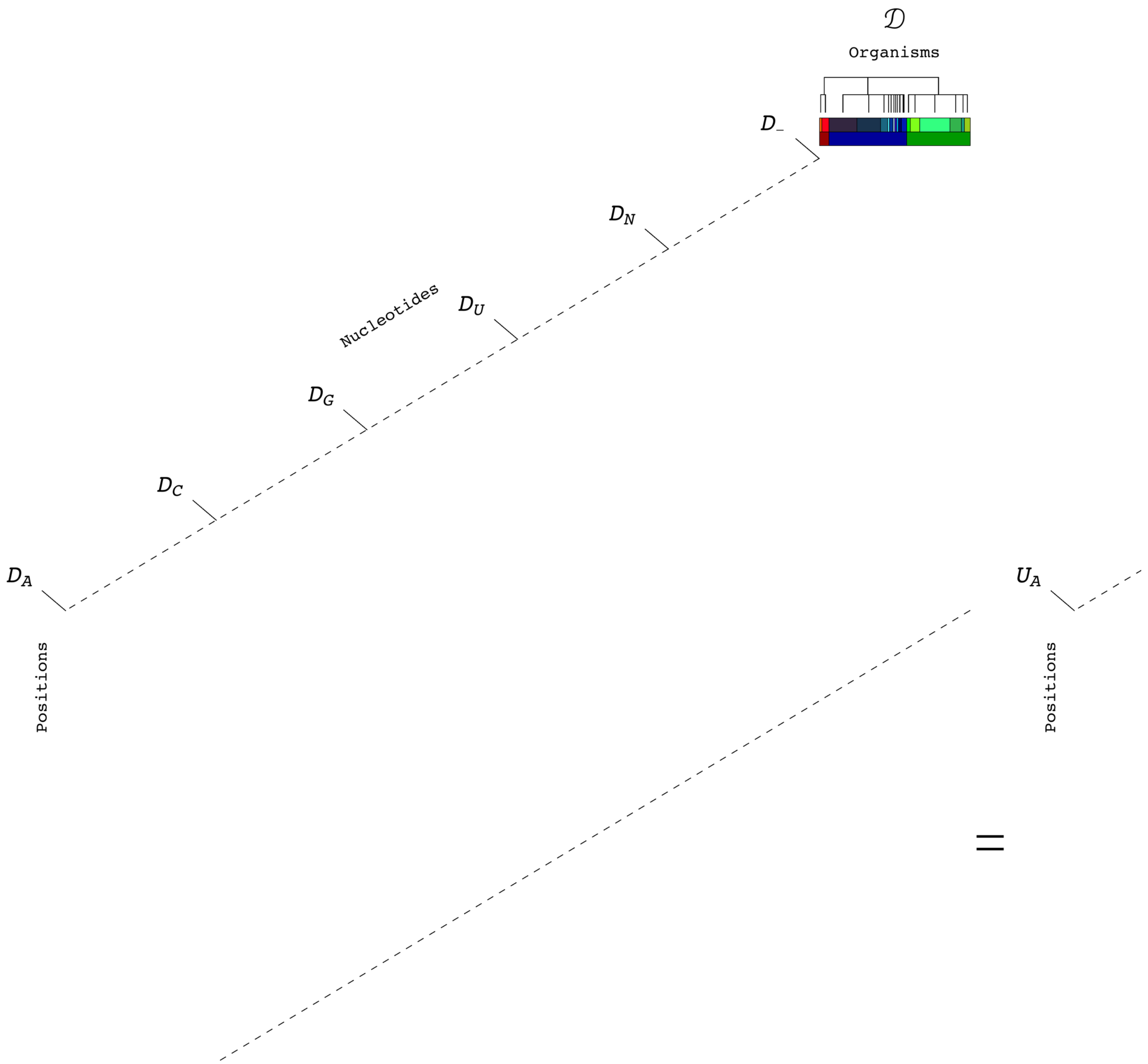

```
(* Create Eigenpositions Line Graph Display *)
```

```
(* Define Constants and Functions for Creating Color Bars and Dashed Lines *)

{topBar, bottomBar} = {.25, -.295};
barHeight = .045;

bar2[group_, color_, position_] :=
  Graphics[{color, EdgeForm[Thickness[.0005]], Rectangle[{Min[parse[group]], position + offset},
     {Max[parse[group]] + 1, position + barHeight + offset}]}];

dottedLines[groupList_, offset_] := {
  lineList = {};
  Do[lineList = Union[lineList, {Min[parse[groupList[[a]]]], Max[parse[groupList[[a]]]] + 1}],
   {a, 1, Dimensions[groupList][[1]]}];
  lineList = Complement[lineList, {1, organisms + 1}];
  lineList = Table[Graphics[{
      Thickness[Medium], Dashed, Opacity[.7], Line[{{lineList[[a]], .25 + offset},
        {lineList[[a]], -.25 + offset}}]}], {a, 1, Dimensions[lineList][[1]]}];
  Show[lineList,
   PlotRange → {{1, 75}, {-.4 + offset, .4 + offset}},
   AspectRatio → 1,
   Frame → True]
 }

createPlots[coordinates1_, coordinates2_, label_, offset_] := {
  points1 = Table[Point[coordinates1[[a]]], {a, 1, organisms}];
  points2 = Table[Point[coordinates2[[a]]], {a, 1, organisms}];
  Show[
   Graphics[{Red, Thickness[.001], Line[coordinates1], PointSize[.007], points1}],
   Graphics[{Blue, Thickness[.001], Line[coordinates2], PointSize[.007], points2}],
   Graphics[Line[{{1, .25 + offset}, {1, -.25 + offset},
      {organisms + 1, -.25 + offset}, {organisms + 1, .25 + offset}}]],
   Graphics[Line[{{1, 0 + offset}, {organisms + 1, 0 + offset}}]],
   Graphics[Text["0.2", {-2, .2 + offset}, {1, 0}]],
   Graphics[Text["-0.2", {-2, -.2 + offset}, {1, 0}]],
   Graphics[Line[{{1, .2 + offset}, {-1, .2 + offset}}]],
   Graphics[Line[{{1, -.2 + offset}, {-1, -.2 + offset}}]],
   Graphics[Text[label, {-10, .295 + offset}, {0, 1}]]]
 }
```

```
(* Create Eigenpositions 2 and 4 Plots *)

offset = 1.3;

domain = {
   bar2["Archaea + Bacteria + Eukarya", RGBColor[.75, .75, .75], bottomBar],
   bar2["Archaea", RGBColor[.6, 0, 0], bottomBar],
   bar2["Gamma_Subdivision", RGBColor[.8, .6, 1], bottomBar],
   bar2["Actinobacteria", RGBColor[.1, .2, .3], bottomBar]};

subgroupings1 = {
   bar2["Archaea + Bacteria + Eukarya", RGBColor[.75, .75, .75], topBar],
   bar2["Bacteria", RGBColor[0, 0, .5], topBar],
   bar2["Eukarya", RGBColor[0, .6, 0], topBar],
   bar2["Microsporidia", RGBColor[.75, .75, .75], topBar]};

lines = dottedLines[{"Archaea", "Bacteria", "Eukarya",
    "Microsporidia", "Gamma_Subdivision", "Actinobacteria"}, offset];

label = "(a)";
coordinates1 = Table[{a + .5, eigenpositions[[2, a]] + offset}, {a, 1, organisms}];
coordinates2 = Table[{a + .5, eigenpositions[[4, a]] + offset}, {a, 1, organisms}];
g = createPlots[coordinates1, coordinates2, label, offset];
eig2and4 = Show[domain, subgroupings1, lines, g];

(* Create Eigenpositions 3 and 5 Plots *)

offset = .67;

domain = {
   bar2["Archaea + Bacteria + Eukarya", RGBColor[.75, .75, .75], bottomBar],
   bar2["Archaea", RGBColor[.6, 0, 0], bottomBar],
   bar2["Microsporidia", RGBColor[0, .3, .1], bottomBar]};

subgroupings1 = {
   bar2["Archaea + Bacteria + Eukarya", RGBColor[.75, .75, .75], topBar],
   bar2["Archaea", RGBColor[.6, 0, 0], topBar],
   bar2["Microsporidia", RGBColor[0, .3, .1], topBar]};

lines = dottedLines[{"Archaea", "Microsporidia"}, offset];

label = "(b)";
coordinates1 = Table[{a + .5, eigenpositions[[3, a]] + offset}, {a, 1, organisms}];
coordinates2 = Table[{a + .5, eigenpositions[[5, a]] + offset}, {a, 1, organisms}];
g = createPlots[coordinates1, coordinates2, label, offset];
eig3and5 = Show[domain, subgroupings1, lines, g];
```

```
(* Create Eigenpositions 6 and 7 Plots *)

offset = .045;

domain = {
   bar2["Archaea + Bacteria + Eukarya", RGBColor[.75, .75, .75], topBar],
   bar2["Fungi/Metazoa", RGBColor[.2, 1, .5], topBar],
   bar2["Microsporidia", RGBColor[.75, .75, .75], topBar],
   bar2["Rhodophyta", RGBColor[.2, .7, .3], topBar]};

subgroupings1 = {
   bar2["Archaea + Bacteria + Eukarya", RGBColor[.75, .75, .75], bottomBar],
   bar2["Alveolata", RGBColor[.5, 1, .1], bottomBar],
   bar2["Fungi/Metazoa", RGBColor[.2, 1, .5], bottomBar],
   bar2["Microsporidia", RGBColor[.75, .75, .75], bottomBar]};

lines = dottedLines[{"Alveolata", "Fungi/Metazoa", "Microsporidia", "Rhodophyta"}, offset];

label = "(c)";
coordinates1 = Table[{a + .5, eigenpositions[[6, a]] + offset}, {a, 1, organisms}];
coordinates2 = Table[{a + .5, eigenpositions[[7, a]] + offset}, {a, 1, organisms}];
g = createPlots[coordinates1, coordinates2, label, offset];
eig6and7 = Show[domain, subgroupings1, lines, g];

(* Define Functions to Generate Color Bars and Line Segments of the Tree *)

yOffset = 1.59;
{level1, level2, level3} = {.48, .83, 1.04};

bar[group_, level_, red_, green_, blue_] :=
 Graphics[{RGBColor[red, green, blue], EdgeForm[Thickness[.0005]],
   Rectangle[{Min[parse[group]], level * barHeight + yOffset},
    {Max[parse[group]] + 1, barHeight + level * barHeight + yOffset}]}]

bar[group_, level_, red_, green_, blue_, height_] := {
  bar[group, level, red, green, blue],
  lineUp[group, .07, height]}

bar[group_, level_, red_, green_, blue_, height_, name2_, height2_] :=
  bar[group, level, red, green, blue, height];

connect[group1_, group2_, height_] := Graphics[Line[
   {{(Min[parse[group1]] + Max[parse[group1]] + 1) / 2, height + barHeight * 3 + yOffset},
    {(Min[parse[group2]] + Max[parse[group2]] + 1) / 2, height + barHeight * 3 + yOffset}}]]
```

```
lineUp[group1_, group2_, startHeight_, endHeight_] := {
  pos = (Min[parse[group1]] + Max[parse[group2]] + 1) / 2;
  Graphics[
   Line[{{pos, startHeight + barHeight * 3 + yOffset}, {pos, endHeight + barHeight * 3 + yOffset}}]]}

lineUp[group_, startHeight_, endHeight_] := lineUp[group, group, startHeight, endHeight];

bar[group_, altName_, level_, red_, green_, blue_, height_] :=
 {bar[group, level, red, green, blue], lineUp[group, .07, height]}

bar[group_, altName_, level_, red_, green_, blue_, height_, name2_, height2_] :=
  bar[group, level, red, green, blue, height, name2, height2];

addText[group_, height_, text_] := Graphics[Text[text,
   {(Min[parse[group]] + Max[parse[group]] + 1) / 2 + 4,
    height + barHeight * 3 + yOffset + .007}, {1, 0}, {0, -1}]]

addText[group_, height_, text_, flag_] := Graphics[Style[Text[" " <> text,
    {(Min[parse[group]] + Max[parse[group]] + 1) / 2 + .5, height + barHeight * 3 + yOffset},
    {1, 0}, {0, -1}], Background → RGBColor[1, 1, 1]]]

(* Create Color Bars and Tree *)

domain = {
   bar["Archaea", 1, .6, 0, 0],
   bar["Bacteria", 1, 0, 0, .6],
   bar["Eukarya", 1, 0, .6, 0]};

subgroupings1 = {
   bar["Crenarchaeota", 2, 1, .4, 0],
   bar["Euryarchaeota", 2, 1, 0, .1],
   bar["Proteobacteria", 2, .2, .15, .25],
   bar["Actinobacteria", 2, .1, .2, .3],
   bar["Firmicutes", 2, .1, .4, .5],
   bar["Tenericutes", 2, .3, .9, 1],
   bar["Planctomycetes", 2, 0, 0, .4],
   bar["Spirochaetes", 2, 0, .2, .6],
   bar["Thermotogae", 2, .6, .8, 1],
   bar["Deinococci", 2, .2, 0, .8],
   bar["Chlamydiae", 2, 0, .7, 1],
   bar["Cyanobacteria", 2, 0, 0, .4],
   bar["Other_Bacteria", 2, 0, .1, .7],
   bar["Other_Eukarya", 2, 0, .9, 0],
   bar["Alveolata", 2, .5, 1, .1],
   bar["Fungi/Metazoa", 2, .2, 1, .5],
   bar["Rhodophyta", 2, .2, .7, .3],
   bar["Viridiplantae", 2, .1, .6, .5],
   bar["Stramenopiles", 2, .6, .8, .1]};
```

```
subgroupings2 = {
   bar["Crenarchaeota", 3, 1, .4, 0, level2],
   bar["Halobacteria+", 3, 1, .6, 0, level1],
   bar["Methanobacteria+", 3, 1, 1, .2, level2, "Euryarchaeota", level1],
   connect["Crenarchaeota", "Methanobacteria+", level2],
   bar["Thermococci+", 3, 1, .2, .2, level1],
   connect["Halobacteria+", "Thermococci+", level1],
   bar["Alpha_Subdivision", "Alpha Subdivision", 3, .4, .3, .5, level1],
   bar["Beta_Subdivision", "Beta Subdivision", 3, .6, .45, .75, level1],
   bar["Gamma_Subdivision",
    "Gamma Subdivision", 3, .8, .6, 1, level2, "Proteobacteria", level1],
   bar["Delta/Epsilon_Subdivisions+", "Delta/Epsilon Subdivisions+", 3, .9, .75, 1, level1],
   connect["Alpha_Subdivision", "Delta/Epsilon_Subdivisions+", level1],
   bar["Corynebacterineae+", 3, .4, .6, .8, level1],
   bar["Streptomycineae", 3, .6, .8, 1, level1],
   connect["Corynebacterineae+", "Streptomycineae", level1],
   lineUp["Actinobacteria", level1, level3],
   addText["Actinobacteria", level1, "Actinobacteria"],
   addText["Actinobacteria", level2, "Bacteria"],
   bar["Bacilli", 3, .15, .6, .9, level1],
   bar["Clostridia", 3, .2, .8, 1, level1],
   bar["Tenericutes", 3, .3, .9, 1, level2],
   connect["Bacilli", "Clostridia", level1],
   addText["Firmicutes", level1, "Firmicutes"],
   lineUp["Firmicutes", level1, level2],
   bar["Spirochaetes", 3, 0, .2, .6, level2],
   bar["Thermotogae", 3, .6, .8, 1, level2],
   bar["Deinococci", 3, .2, 0, .8, level2],
   bar["Chlamydiae", 3, .0, .7, 1, level2],
   bar["Cyanobacteria", 3, 0, 0, .4, level2],
   bar["Other_Bacteria", "Other Bacteria", 3, 0, .1, .7, level2],
   connect["Gamma_Subdivision", "Other_Bacteria", level2],
   bar["Apicomplexa", 3, .2, .6, 0, level1],
   bar["Ciliophora+", 3, .1, .3, .15, level1],
   bar["Other_Eukarya", "Other Eukarya", 3, 0, .9, 0, level2],
   connect["Apicomplexa", "Ciliophora+", level1],
   lineUp["Alveolata", level1, level2],
   addText["Alveolata", level1, "Alveolata"],
   bar["Other_Fungi/Metazoa", "Other Fungi/Metazoa", 3, 0, .8, .25, level1],
   bar["Fungi", 3, 0, .6, .2, level1],
   bar["Microsporidia", 3, 0, .3, .1, level3, "Fungi/Metazoa", level1],
   addText["Microsporidia", level2, "Eukarya"],
   connect["Archaea", "Microsporidia", level3],
   lineUp["Archaea", level2, level3],
   addText["Archaea", level2, "Archaea"],
   bar["Metazoa", 3, .6, .7, .4, level1],
   connect["Other_Fungi/Metazoa", "Metazoa", level1],
   bar["Bangiophyceae+", 3, .2, .67, .5, level1],
   bar["Florideophyceae", 3, .15, .5, .35, level1],
   addText["Rhodophyta", level1, "Rhodophyta"],
   connect["Bangiophyceae", "Florideophyceae", level1],
   lineUp["Rhodophyta", level1, level2],
   bar["Viridiplantae", 3, .1, .6, .5, level2],
   connect["Other_Eukarya", "Stramenopiles", level2],
   bar["Stramenopiles", 3, .6, .8, .1, level2]};
```

```
text = {
   addText["Actinobacteria", level2, "Bacteria"],
   addText["Archaea", level2, "Archaea"],
   addText["Microsporidia", level2, "Eukarya"],
   addText["Rhodophyta", level1, "Rhodophyta"],
   addText["Microsporidia", level1, "Fungi/Metazoa"],
   addText["Gamma_Subdivision", level1, "Proteobacteria"],
   addText["Gamma_Subdivision", level1 - .415, "Gamma Subdivision", 1],
   addText["Actinobacteria", level1, "Actinobacteria"],
   addText["Alveolata", level1, "Alveolata"],
   addText["Microsporidia", level1 - .415, "Microsporidia", 1]};

(* Show Eigenpositions Line Graph Display *)

f2 = Show[{eig2and4, eig3and5, eig6and7, domain, subgroupings1, subgroupings2, text,
   Graphics[{Yellow, Opacity[0], Rectangle[{organisms, 1.47}, {organisms + 8, -.225}]}],
   Graphics[Text["Organisms", {organisms / 2, 2.8}, {1, 0}]],
   Graphics[Text["Eigenposition", {organisms + 15, .53}, {1, 0}, {0, -1}]],
   Graphics[Text["-2", {organisms + 8, 1.575}, {1, 0}]],
   Graphics[Text["-4", {organisms + 8, 1.03}, {1, 0}]],
   Graphics[Text["-3", {organisms + 8, .945}, {1, 0}]],
   Graphics[Text["-5", {organisms + 8, .4}, {1, 0}]],
   Graphics[Text["-6", {organisms + 8, .323}, {1, 0}]],
   Graphics[Text["-7", {organisms + 8, -.225}, {1, 0}]],
   Graphics[Text["Relative Nucletide Frequency", {-28, .68}, {0, 0}, {0, 1}]]},
  AspectRatio → 1.5, ImageSize → 550, BaseStyle → { FontSize → 10, FontFamily → "Courier"}]
```

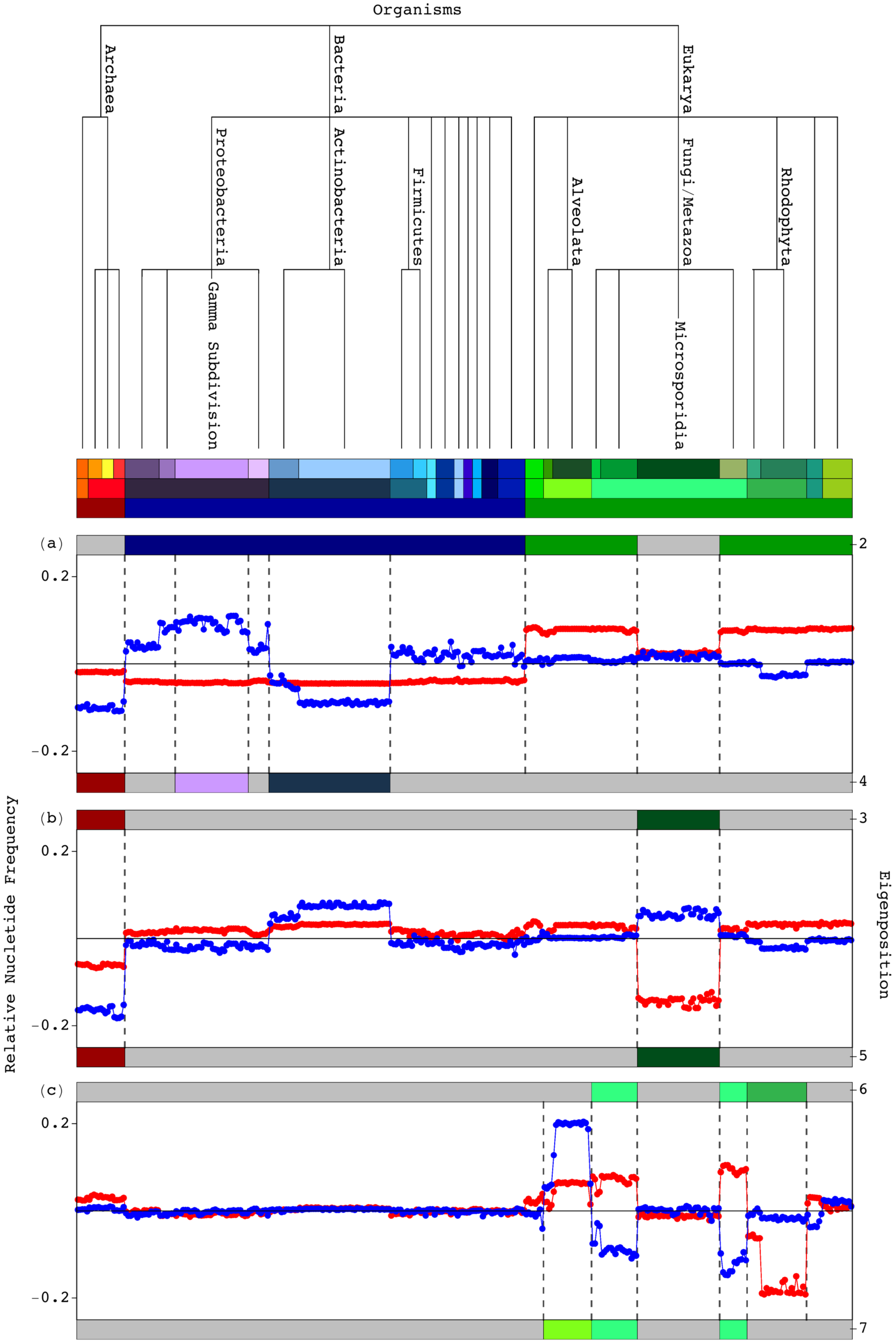
Organisms
Archaea
Bacteria
Eukarya
Proteobacteria
Actinobacteria
Firmicutes
Alveolata
Fungi/Metazoa
Rhodophyta
Gamma Subdivision
Microsporidia
(a)
(b)
(c)
0.2
-0.2
Relative Nucleotide Frequency
Eigenposition
2
4
3
5
6
7

```
(* Create Raster Display of Sigificant Eigenpositions with Taxonomy Annotation *)
```

```
(* Bar Chart Display of Fractions of Relative Nucleotide Frequency *)

x = 16;
gridx = Table[a, {a, 0, .12, .02}];
framex = Table[{gridx[[a]], Rotate[Round[gridx[[a]], .01], 90 Degree]}, {a, 1, 7}];
framex[[1, 2]] = Rotate["0   ", 90 Degree];
gridx = Table[gridx[[a]], {a, 1, 7}];
framey = Table[{a + 1, x - a - 6}, {a, 0, x - 6 - 1}];
insetBar = BarChart[
   Table[fractions[[x - a]], {a, 6, x - 2}],
   BarOrigin → Left,
   PlotRange → {{0, .13 * 1.0001}, {.5, x - 6 - 1 + .5}},
   Axes → False,
   Frame → True,
   ChartStyle → Red,
   FrameTicks → {None, framey, framex, None},
   GridLines → {gridx, None},
   AspectRatio → 1.1,
   BaseStyle → { FontSize → 11, FontFamily → "Courier"}];

x = 25;
gridx = Table[a, {a, 0, .75, .15}];
framex = Table[{gridx[[a]], Rotate[Round[gridx[[a]], .05], 90 Degree]}, {a, 1, 6}];
framex[[1, 2]] = Rotate["0    ", 90 Degree];
framey = Table[{a + 1, x - a}, {a, 0, x - 1}];
fractionChart = BarChart[
   Table[fractions[[x - a]], {a, 0, x - 1}],
   BarOrigin → Left,
   PlotRange → {{0, .75 * 1.0001}, {.5, x + .75}},
   ChartStyle → Red,
   Epilog → Inset[insetBar, {.16, .25}, {0, 0}, {.65, 22}, {1, 0}, Background → LightYellow],
   Axes → False,
   Frame → True,
   FrameTicks → {None, framey, framex, None},
   GridLines → {gridx, None},
   AspectRatio → 1,
   BaseStyle → { FontSize → 11, FontFamily → "Courier"}];
```

```
(* Create 2D Red & Green Raster Display of Sigificant Eigenpositions *)

contrast = 5;
displaying = Table[
   adjustedValue = contrast * eigenpositions[[i, j]];
   If[adjustedValue > 0,
    If[adjustedValue < 1,
     {adjustedValue, 0, 0}, {1, 0, 0}],
    If[adjustedValue > -1,
     {0, -adjustedValue, 0}, {0, 1, 0}]],
   {i, 25, 1, -1}, {j, 1, organisms}];
labely = "(b) Eigenpositions";

raster = {
   Graphics[Raster[displaying, {{1, -1}, {organisms + 1, -.02}}]],
   Graphics[Text[labely, {-9, -.98 / 2}, {0, -1}, {0, 1}], 11],
   Table[{
     Graphics[Text[a, {0, -.02 - (.98 / 25) * (a - .4)}, {1, 0}]],
     Graphics[Line[{{1, -.02 - (.98 / 25) * (a - .5)}, {2, -.02 - (.98 / 25) * (a - .5)}}]]},
    {a, 1, 25}]
  };

(* Define Functions to Generate Color Bars and Line Segments of the Tree *)

Clear[bar, connect, lineUp, addText]

bar[group_, level_, red_, green_, blue_] :=
 Graphics[{RGBColor[red, green, blue], EdgeForm[Thickness[.0003]], Rectangle[
    {Min[parse[group]], level * .08 - .06}, {Max[parse[group]] + 1, .08 + level * .08 - .06}]}]

bar[group_, altName_, level_, red_, green_, blue_, height_] := {
   bar[group, level, red, green, blue],
   pos = (Min[parse[group]] + Max[parse[group]] + 1) / 2;
   Graphics[Line[{{pos, .33}, {pos, height * .5 + .4}}]],
   Graphics[
    Style[Text[" " <> altName, {pos, .3}, {1, 0}, {0, -1}], Background → RGBColor[1, 1, 1]]]};

bar[group_, level_, red_, green_, blue_, height_] :=
  bar[group, group, level, red, green, blue, height];

bar[group_, level_, red_, green_, blue__, height_, group2_, height2_] := {
   bar[group, level, red, green, blue, height], addText[group, height2, group2]};

connect[group1_, group2_, height_] :=
  Graphics[Line[{{(Min[parse[group1]] + Max[parse[group1]] + 1) / 2, height * .5 + .4},
     {(Min[parse[group2]] + Max[parse[group2]] + 1) / 2, height * .5 + .4}}]];

lineUp[group1_, group2_, startHeight_, endHeight_] := {
   pos = (Min[parse[group1]] + Max[parse[group2]] + 1) / 2;
   Graphics[Line[{{pos, startHeight * .5 + .4}, {pos, endHeight * .5 + .4}}]]};

lineUp[group_, startHeight_, endHeight_] := lineUp[group, group, startHeight, endHeight];

addText[group1_, group2_, height_, text_] := Graphics[Style[Text[
     text, {(Min[parse[group1]] + Max[parse[group2]] + 1) / 2 + 4, height * .5 + .43}, {1, 0}, {0, -1}],
    Background → RGBColor[1, 1, 1]]];

addText[group_, height_, text_] := addText[group, group, height, text];
```

```
(* Create Color Bars and Tree *)

{level1, level2, level3} = {1.9, 3, 3.7};
domain = {
   bar["Archaea", 1, .6, 0, 0],
   bar["Bacteria", 1, 0, 0, .6],
   bar["Eukarya", 1, 0, .6, 0]};

subgroupings1 = {
   bar["Crenarchaeota", 2, 1, .4, 0],
   bar["Euryarchaeota", 2, 1, 0, .1],
   bar["Proteobacteria", 2, .2, .15, .25],
   bar["Actinobacteria", 2, .1, .2, .3],
   bar["Firmicutes", 2, .1, .4, .5],
   bar["Tenericutes", 2, .3, .9, 1],
   bar["Planctomycetes", 2, 0, 0, .4],
   bar["Spirochaetes", 2, 0, .2, .6],
   bar["Thermotogae", 2, .6, .8, 1],
   bar["Deinococci", 2, .2, 0, .8],
   bar["Chlamydiae", 2, 0, .7, 1],
   bar["Cyanobacteria", 2, 0, 0, .4],
   bar["Other_Bacteria", 2, 0, .1, .7],
   bar["Other_Eukarya", 2, 0, .9, 0],
   bar["Alveolata", 2, .5, 1, .1],
   bar["Fungi/Metazoa", 2, .2, 1, .5],
   bar["Rhodophyta", 2, .2, .7, .3],
   bar["Viridiplantae", 2, .1, .6, .5],
   bar["Stramenopiles", 2, .6, .8, .1]};
```

```
subgroupings2 = {
   bar["Crenarchaeota", 3, 1, .4, 0, level2],
   bar["Halobacteria+", 3, 1, .6, 0, level1],
   bar["Methanobacteria+", 3, 1, 1, .2, level2, "Euryarchaeota", level1],
   connect["Crenarchaeota", "Methanobacteria+", level2],
   bar["Thermococci+", 3, 1, .2, .2, level1],
   connect["Halobacteria+", "Thermococci+", level1],
   bar["Alpha_Subdivision", "Alpha Subdivision", 3, .4, .3, .5, level1],
   bar["Beta_Subdivision", "Beta Subdivision", 3, .6, .45, .75, level1],
   bar["Gamma_Subdivision",
    "Gamma Subdivision", 3, .8, .6, 1, level2, "Proteobacteria", level1],
   bar["Delta/Epsilon_Subdivisions+", "Delta/Epsilon Subdivisions+", 3, .9, .75, 1, level1],
   connect["Alpha_Subdivision", "Delta/Epsilon_Subdivisions+", level1],
   bar["Corynebacterineae+", 3, .4, .6, .8, level1],
   bar["Streptomycineae", 3, .6, .8, 1, level1],
   connect["Corynebacterineae+", "Streptomycineae", level1],
   lineUp["Actinobacteria", level1, level3],
   addText["Actinobacteria", level1, "Actinobacteria"],
   addText["Actinobacteria", level2, "Bacteria"],
   bar["Bacilli", 3, .15, .6, .9, level1],
   bar["Clostridia", 3, .2, .8, 1, level1],
   bar["Tenericutes", 3, .3, .9, 1, level2],
   connect["Bacilli", "Clostridia", level1],
   addText["Firmicutes", level1, "Firmicutes"],
   lineUp["Firmicutes", level1, level2],
   bar["Spirochaetes", 3, 0, .2, .6, level2],
   bar["Thermotogae", 3, .6, .8, 1, level2],
   bar["Deinococci", 3, .2, 0, .8, level2],
   bar["Chlamydiae", 3, .0, .7, 1, level2],
   bar["Cyanobacteria", 3, 0, 0, .4, level2],
   bar["Other_Bacteria", "Other Bacteria", 3, 0, .1, .7, level2],
   connect["Gamma_Subdivision", "Other_Bacteria", level2],
   bar["Apicomplexa", 3, .2, .6, 0, level1],
   bar["Ciliophora+", 3, .1, .3, .15, level1],
   bar["Other_Eukarya", "Other Eukarya", 3, 0, .9, 0, level2],
   connect["Apicomplexa", "Ciliophora+", level1],
   lineUp["Alveolata", level1, level2],
   addText["Alveolata", level1, "Alveolata"],
   bar["Other_Fungi/Metazoa", "Other Fungi/Metazoa", 3, 0, .8, .25, level1],
   bar["Fungi", 3, 0, .6, .2, level1],
   bar["Microsporidia", 3, 0, .3, .1, level3, "Fungi/Metazoa", level1],
   addText["Microsporidia", level2, "Eukarya"],
   connect["Archaea", "Microsporidia", level3],
   lineUp["Archaea", level2, level3],
   addText["Archaea", level2, "Archaea"],
   bar["Metazoa", 3, .6, .7, .4, level1],
   connect["Other_Fungi/Metazoa", "Metazoa", level1],
   bar["Bangiophyceae+", 3, .2, .67, .5, level1],
   bar["Florideophyceae", 3, .15, .5, .35, level1],
   addText["Rhodophyta", level1, "Rhodophyta"],
   connect["Bangiophyceae", "Florideophyceae", level1],
   lineUp["Rhodophyta", level1, level2],
   bar["Viridiplantae", 3, .1, .6, .5, level2],
   connect["Other_Eukarya", "Stramenopiles", level2],
   bar["Stramenopiles", 3, .6, .8, .1, level2]};
```

```
(* Show Sigificant Eigenpositions with Annotation *)

s1 = Show[{raster, subgroupings2, subgroupings1,
    domain, Graphics[{Opacity[0], Rectangle[{0, 0}, {440, -1}]}],
    Graphics[Text["(c) Fraction of\nNucleotide Frequency", {390, .215}, {0, 0}]],
    Graphics[Text["(a) Organisms", {organisms / 2.3, 2.3}, {-1, 0}]]},
  Epilog → Inset[fractionChart, {352, -1.025}, {0, 0}, {87.5, 10}],
  AspectRatio → .6,
  BaseStyle → { FontSize → 11, FontFamily → "Courier"},
  ImageSize → 1150]
```

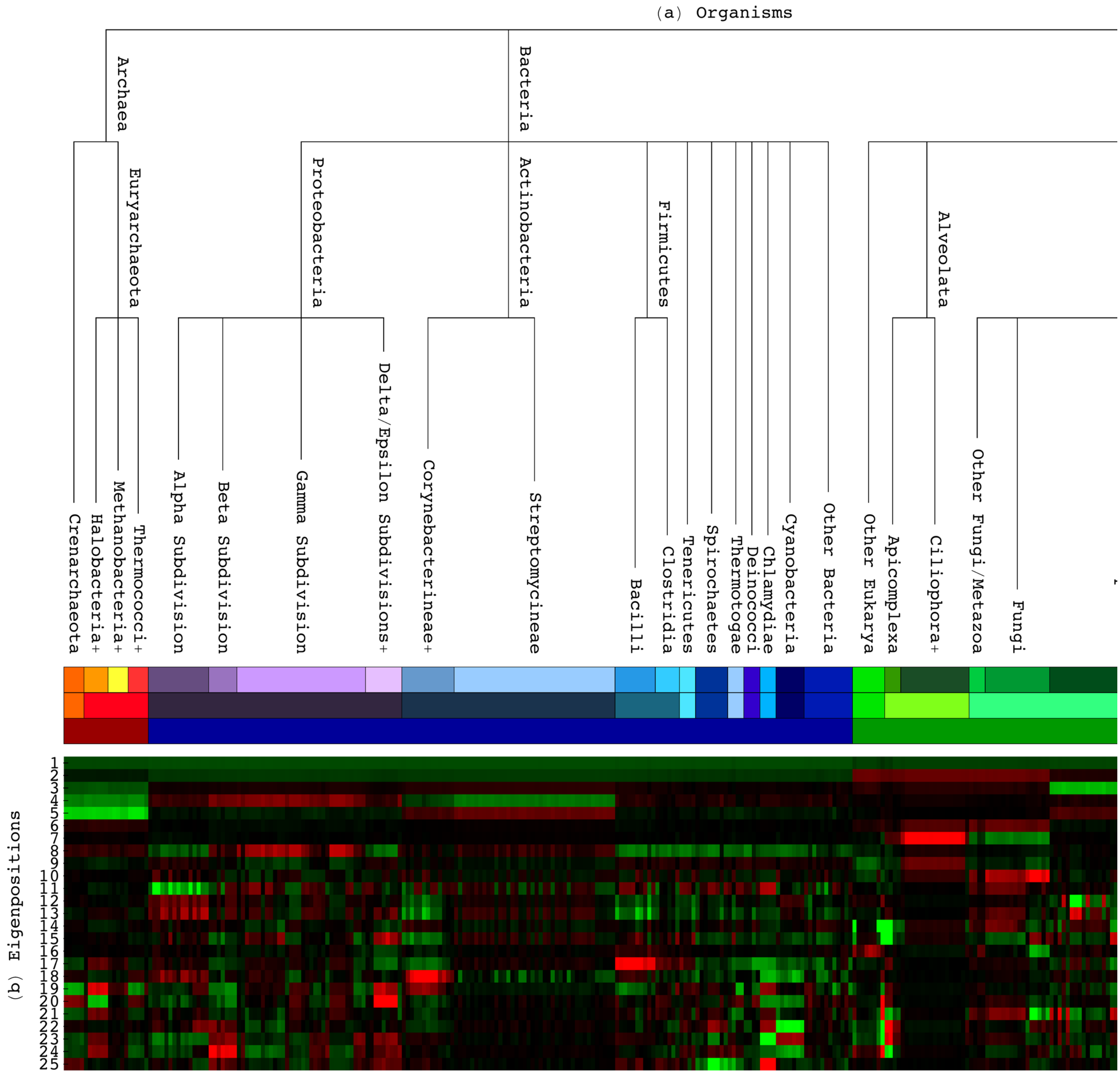

```
(* Create Raster Displays for Selected Positions of the Alignment *)

(* Define Function to Generate Raster *)

segmentMap = {"A", "C", "G", "U", "N", "Gap"};

getSelectedAlignment[segment_, pattern_, tail_, offset_, showPositions_, groupSize_,
  mitoOffset_] := {selectedVector = eigenorganisms[[Range[segment, positions * 6, 6], pattern]];
  posList = If[tail == "Anticorrelated", Range[groupSize, 1, -1],
    Range[positions - groupSize + 1, positions]];
  selectedPositions = Sort[Table[{a, selectedVector[[a]]}, {a, 1, positions}],
     OrderedQ[{{#1[[2]]}, {#2[[2]]}}] &][[posList, 1]];
  selectedAlignment = Transpose[alignment[[All, selectedPositions]]];
  selectedAlignment =
   ReplaceAll[selectedAlignment, {"A" → 1, "C" → 2, "G" → 3, "U" → 4, "N" → 5, "-" → 6}];
  If[showPositions, {
    yLabelPosition = -40 - 20 * mitoOffset;
    positionLabels = {Table[Graphics[
        Text[Style[selectedPositions[[a]], 8], {-3 + offset, 100 - groupSize + a - .5}, {1, 0}]],
       {a, 1, groupSize}],
      Table[Graphics[Line[{{0 + offset, 100 - groupSize + a - .5}, {5 + offset, 100 - groupSize +
            a - .5}}]], {a, 1, groupSize}]}}, yLabelPosition = -15 - 7.5 * mitoOffset];
  outputRaster = {
    Graphics[Raster[selectedAlignment, {{offset, 100 - groupSize}, {offset + organisms, 100}},
      ColorFunction → (Switch[#, 1, Red, 2, RGBColor[0, .5, 0], 3, Blue, 4,
          Yellow, 5, RGBColor[.5, .5, .5], 6, Black] &)], AspectRatio → 2],
    Graphics[Text["Positions of " <> segmentMap[[segment]] <> " Variation Most " <>
       tail <> " with Eigenposition " <> ToString[pattern],
      {yLabelPosition + offset, 99}, {1, 0}, {0, 1}]]};
  If[showPositions, outputRaster = {outputRaster, positionLabels}];
  outputRaster
 }

(* Define Taxonomic Grouping Bars for Selected Eigenpositions *)

bar[color_, start_, end_, offset_] :=
  Graphics[{color, EdgeForm[Thin], Rectangle[{start + offset, 101.25}, {end + offset, 104}]}];

eigen2Taxomomy[offset_, label_] := {
   bar[RGBColor[.75, .75, .75], 1, 21, offset],
   bar[RGBColor[0, 0, .6], 21, 196, offset],
   bar[RGBColor[0, .6, 0], 196, 244, offset],
   bar[RGBColor[.75, .75, .75], 244, 279, offset],
   bar[RGBColor[0, .6, 0], 279, 339, offset],
   Graphics[Text["Bacteria", {108.5 + offset, 105}, {0, -1}]],
   Graphics[Text["Eukarya-", {267.5 + offset, 107}, {0, -1}]],
   Graphics[Text["Microsporidia", {267.5 + offset, 105}, {0, -1}]],
   Graphics[Text[label <> " Organisms", {organisms / 2 + offset, 111.5}, {0, 1}]]};

eigen3Taxomomy[offset_, label_] := {
   bar[RGBColor[.6, 0, 0], 1, 21, offset],
   bar[RGBColor[.75, .75, .75], 21, 244, offset],
   bar[RGBColor[0, .3, .1], 244, 279, offset],
   bar[RGBColor[.75, .75, .75], 279, 339, offset],
   Graphics[Text["Archaea", {17 + offset, 105}, {0, -1}]],
   Graphics[Text["Microsporidia", {261.5 + offset, 105}, {0, -1}]],
   Graphics[Text[label <> " Organisms", {organisms / 2 + offset, 110.5}, {0, 1}]]};
```

```
eigen5Taxomomy[offset_, label_] := eigen3Taxomomy[offset, label];

(* Show Rasters of Positions with Greatest Relative Nucleotide Frequency Variation *)

f3a = Show[
   eigen2Taxomomy[0, ""],
   getSelectedAlignment[6, 2, "Correlated", 0, False, 124, 0],
   BaseStyle → { FontSize → 12, FontFamily → "Courier"},
   AspectRatio → 2, ImageSize → 720 / 2];
f3b = Show[
   eigen2Taxomomy[400, ""],
   getSelectedAlignment[6, 2, "Anticorrelated", 400, False, 100, 0],
   BaseStyle → { FontSize → 12, FontFamily → "Courier"},
   AspectRatio → 1.6, ImageSize → 720 / 2];
s4 = Show[
  eigen2Taxomomy[0, "(a)"],
  eigen2Taxomomy[408, "(b)"],
  getSelectedAlignment[6, 2, "Correlated", 0, True, 124, 0],
  getSelectedAlignment[6, 2, "Anticorrelated", 408, True, 100, 0],
  BaseStyle → { FontSize → 12, FontFamily → "Courier"},
  AspectRatio → 1, ImageSize → 750]
```

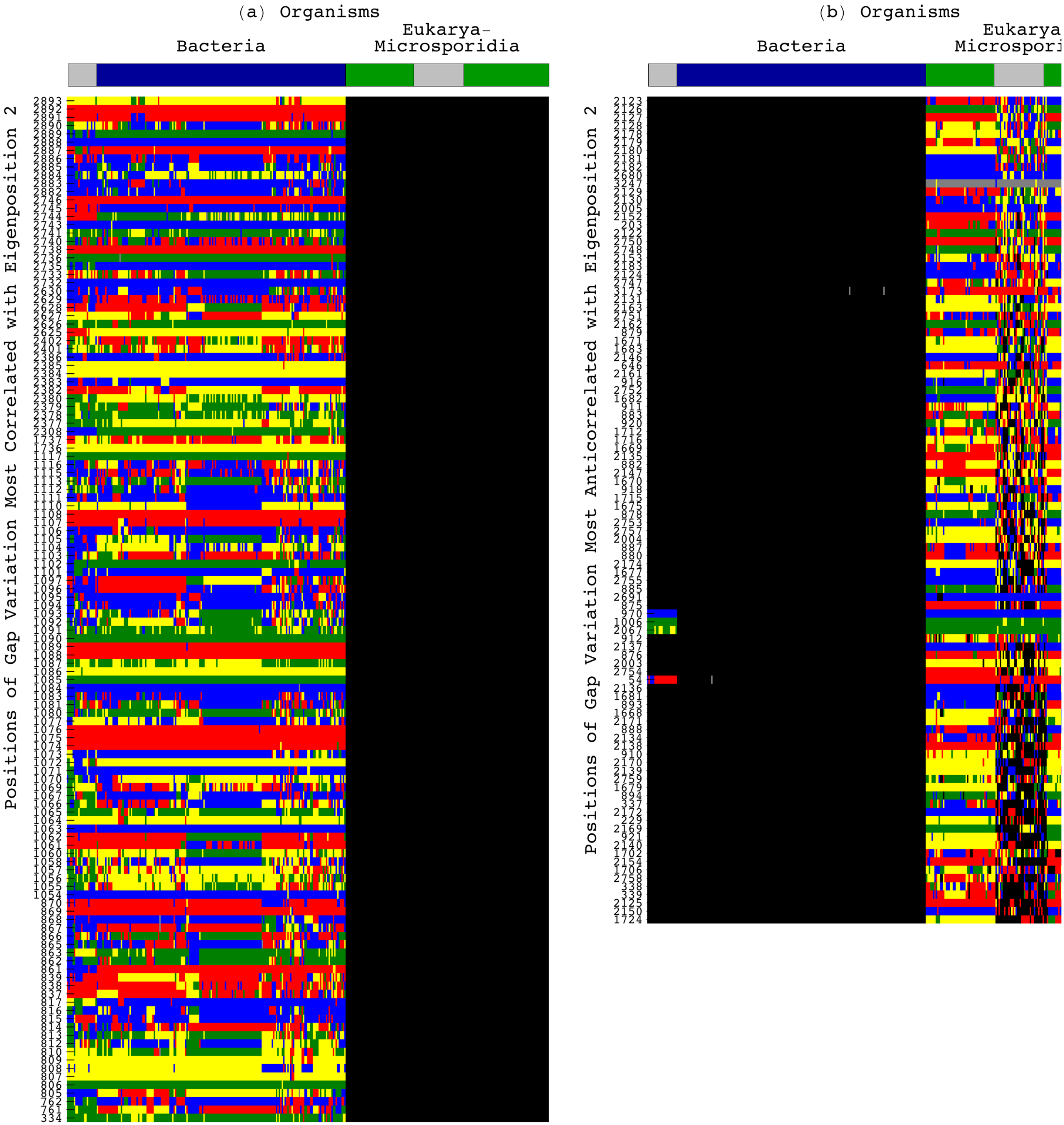
(a) Organisms
Bacteria
Eukarya-
Microsporidia
Positions of Gap Variation Most Correlated with Eigenposition 2
(b) Organisms
Bacteria
Eukarya
Microspori
Positions of Gap Variation Most Anticorrelated with Eigenposition 2

```
f4 = Show[
    eigen2Taxomomy[400, ""],
    getSelectedAlignment[1, 2, "Anticorrelated", 400, False, 100, 0],
    BaseStyle → { FontSize → 12, FontFamily → "Courier"},
    ImageSize → 720 / 2, AspectRatio → 1.6];
s7 = Show[
    eigen2Taxomomy[0, ""],
    getSelectedAlignment[1, 2, "Correlated", 0, False, 100, 0],
    BaseStyle → { FontSize → 12, FontFamily → "Courier"},
    ImageSize → 720 / 2, AspectRatio → 1.6];
s8 = Show[
   eigen2Taxomomy[0, "(a)"],
   eigen2Taxomomy[408, "(b)"],
   getSelectedAlignment[1, 2, "Correlated", 0, True, 100, 0],
   getSelectedAlignment[1, 2, "Anticorrelated", 408, True, 100, 0],
   BaseStyle → { FontSize → 12, FontFamily → "Courier"},
   ImageSize → 750, AspectRatio → .8]
```

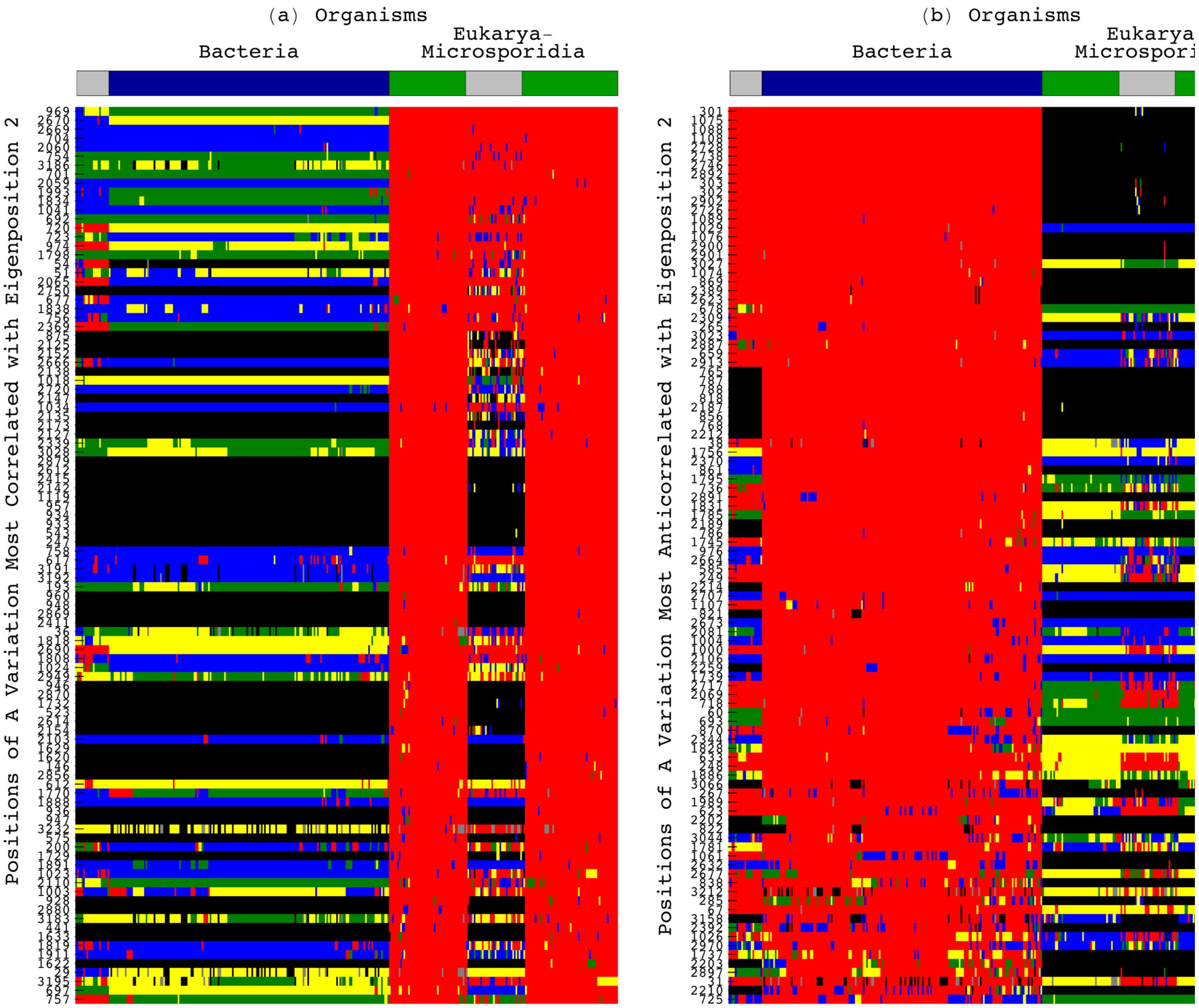

```
s12 = Show[
  eigen3Taxomomy[-408, "(a)"],
  eigen3Taxomomy[0, "(b)"],
  eigen5Taxomomy[408, "(c)"],
  getSelectedAlignment[2, 3, "Anticorrelated", -408, True, 100, 0],
  getSelectedAlignment[3, 3, "Anticorrelated", 0, True, 100, 0],
  getSelectedAlignment[4, 3, "Anticorrelated", 408, True, 100, 0],
  BaseStyle → {FontSize → 12, FontFamily → "Courier"},
  AspectRatio → .534, ImageSize → 1125]
```

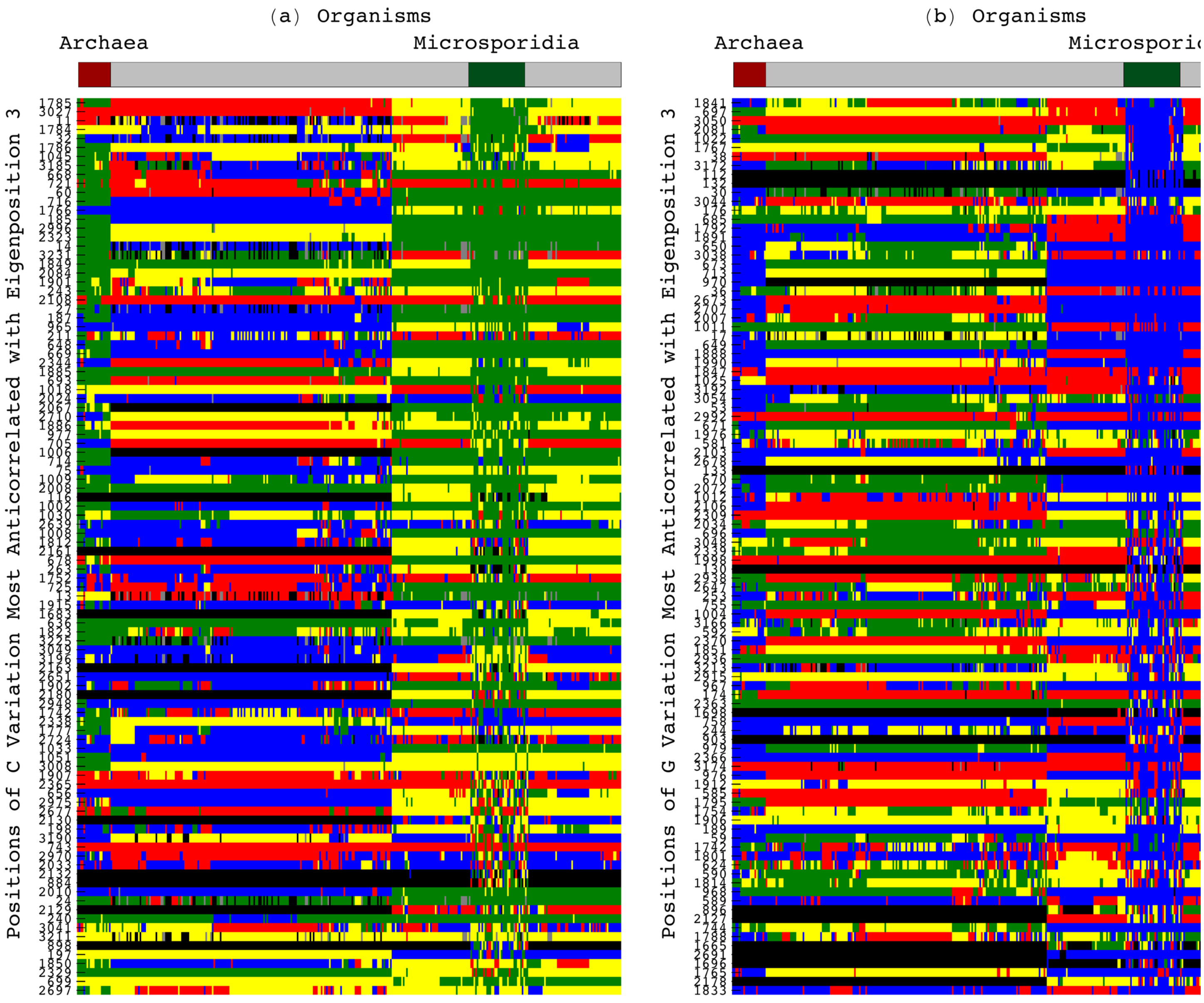

```
s13 = Show[
  eigen5Taxomomy[-408, "(a)"],
  eigen5Taxomomy[0, "(b)"],
  eigen5Taxomomy[408, "(c)"],
  getSelectedAlignment[2, 5, "Correlated", -408, True, 100, 0],
  getSelectedAlignment[4, 5, "Correlated", 0, True, 100, 0],
  getSelectedAlignment[6, 5, "Correlated", 408, True, 100, 0],
  BaseStyle → { FontSize → 12, FontFamily → "Courier"},
  ImageSize → 1125 , AspectRatio → .534]
```

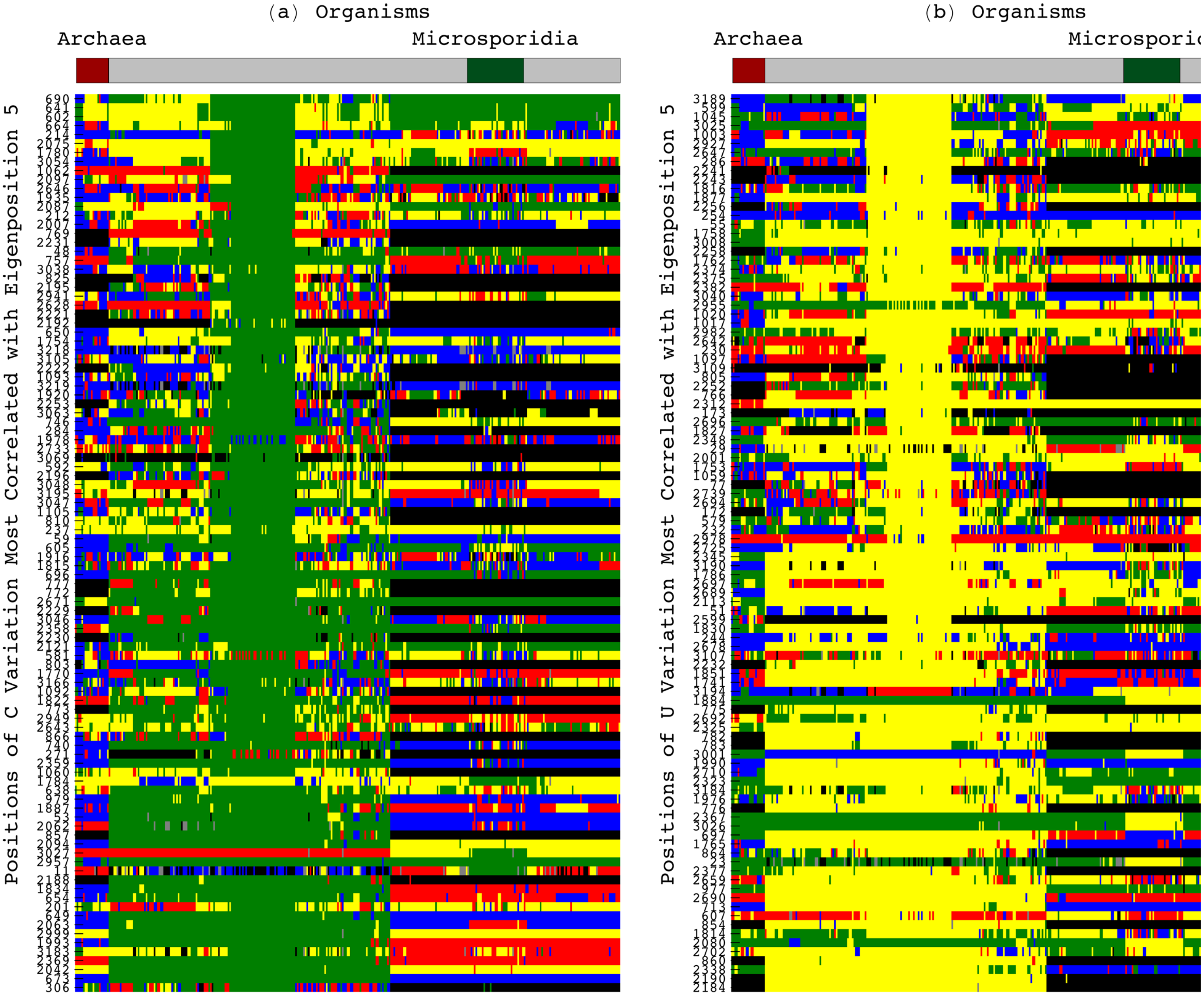

```
s14a = Show[
  eigen5Taxomomy[0, "(a)"],
  eigen5Taxomomy[408, "(b)"],
  getSelectedAlignment[1, 5, "Anticorrelated", 0, True, 100, 0],
  getSelectedAlignment[2, 5, "Anticorrelated", 408, True, 100, 0],
  BaseStyle → {FontSize → 12, FontFamily → "Courier"},
  AspectRatio → .8, ImageSize → 750]
```

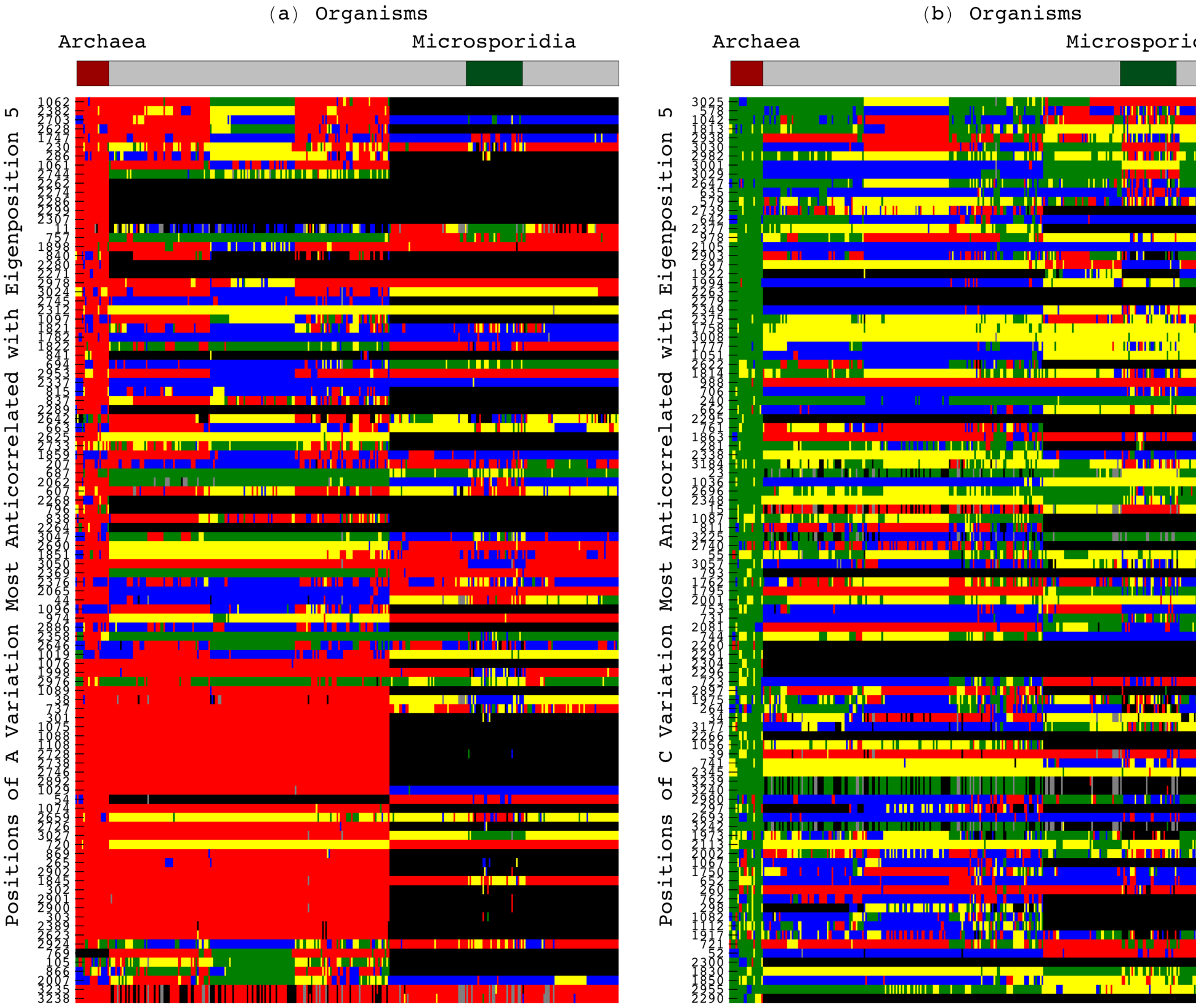

```
s14b = Show[
  eigen5Taxomomy[0, "(c)"],
  eigen5Taxomomy[408, "(d)"],
  getSelectedAlignment[3, 5, "Anticorrelated", 0, True, 100, 0],
  getSelectedAlignment[4, 5, "Anticorrelated", 408, True, 100, 0],
  BaseStyle → {FontSize → 12, FontFamily → "Courier"},
  AspectRatio → .8, ImageSize → 750]
```

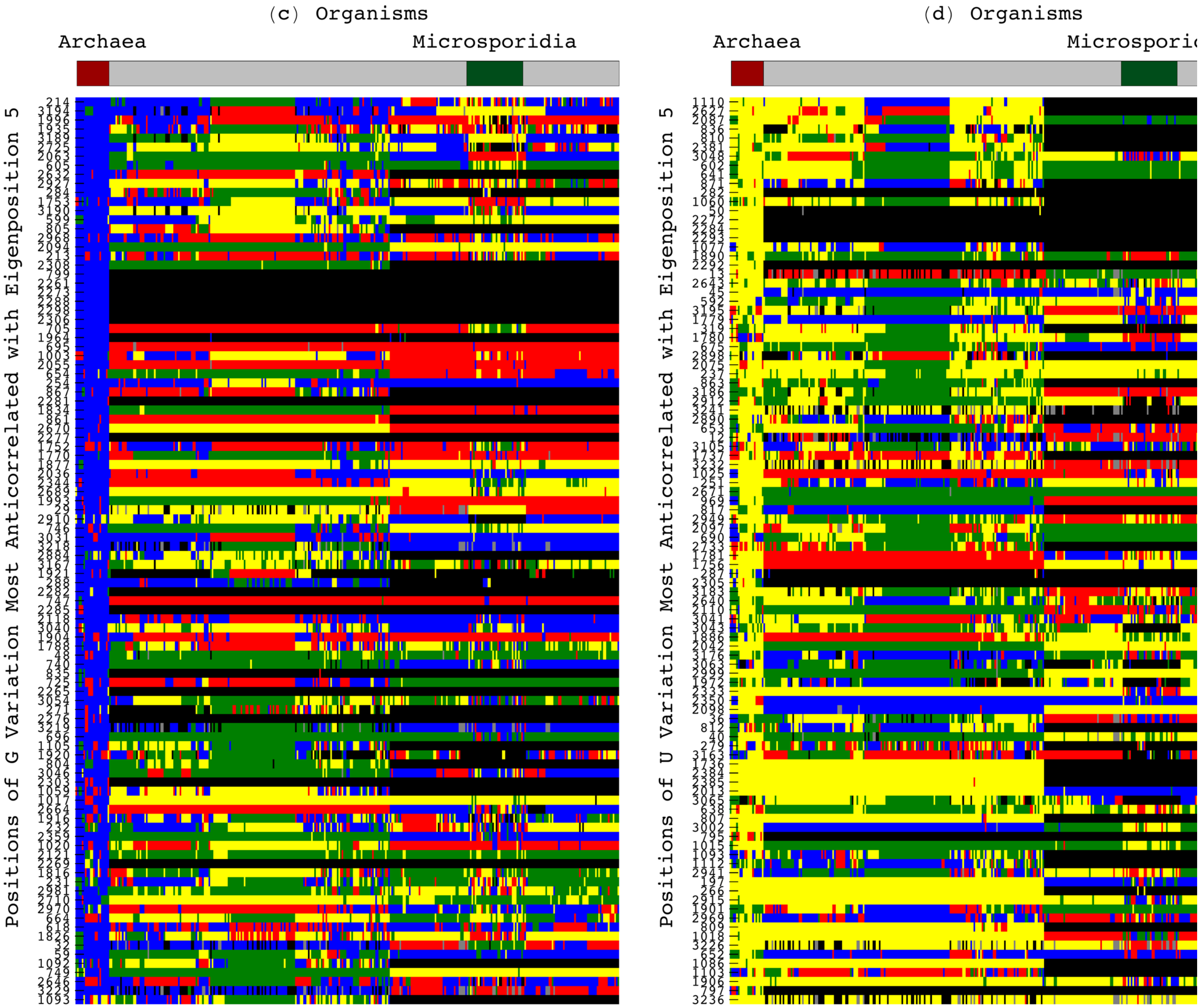

```
(* Compare Significant Alignment Positions with Mitochandrial 16S rRNA Sequences  *)

segment = 6;
pattern = 3;
offset = -376;
labelY = "Positions of Gap Variation Most Anticorrelated with Eigenosition 3";

selectedVector = eigenorganisms[[Range[segment, positions * 6, 6], pattern]];
selectedPositions = Sort[Table[{a, selectedVector[[a]]}, {a, 1, positions}],
     OrderedQ[{{#1[[2]]}, {#2[[2]]}}] &][[1 ;; 100, 1]];
selectedAlignment = Transpose[alignment[[All, selectedPositions]]];
selectedAlignment =
  ReplaceAll[selectedAlignment, {"A" → 1, "C" → 2, "G" → 3, "U" → 4, "N" → 5, "-" → 6}];

negativeCorr = {
   Graphics[Raster[Reverse[selectedAlignment], {{offset, 0}, {offset + organisms, 100}},
     ColorFunction → (Switch[#, 1, Red, 2, RGBColor[0, .5, 0], 3, Blue, 4,
          Yellow, 5, RGBColor[.5, .5, .5], 6, Black] &)], AspectRatio → 1.6],
   Table[Graphics[Text[Style[selectedPositions[[101 - a]], 8], {-3 + offset, a - .5}, {1, 0}]],
    {a, 1, 100}],
   Table[Graphics[Line[{{0 + offset, a - .5}, {5 + offset, a - .5}}]], {a, 1, 100}],
   Graphics[Text[labelY, {-64 + offset, 99}, {1, 0}, {0, 1}]]};

stream = path <> "Dataset_S7.txt";
mito = Import[stream, "Table"];
mito = mito[[All, 2 ;; Dimensions[mito][[2]]]];

mitoSequence = mito[[5 ;; positions + 4]];
mitoTaxonomy = mito[[4]];

mitoOrganisms = Dimensions[mitoSequence][[2]];
mitoSequence = mitoSequence[[selectedPositions]];
mitoSequence = ReplaceAll[mitoSequence, {"A" → 1, "C" → 2, "G" → 3, "U" → 4, "N" → 5, "-" → 6}];
```

```
parseMito[group_] := {
  regX = StringSplit[group, " + "];
  If[Dimensions[regX][[1]] > 1, {
    groupPositions = {},
    Do[groupPositions = Union[groupPositions, Flatten[Position[mitoTaxonomy, regX[[i]]]]]
     , {i, 1, Dimensions[regX][[1]]}]},
   groupPositions = Flatten[Position[mitoTaxonomy, group]]];
  {Min[groupPositions], Max[groupPositions] + 1}
 }

mitoRaster[mitoText_] := {
   Graphics[
    Raster[Reverse[mitoSequence], ColorFunction → (Switch[#, 1, Red, 2, RGBColor[0, .5, 0], 3,
         Blue, 4, Yellow, 5, RGBColor[.5, .5, .5], 6, Black] &)], AspectRatio → 1.6],
   Table[Graphics[Line[{{0, a - .5}, {5, a - .5}}]], {a, 1, 100}],
   Graphics[Text[mitoText, {mitoOrganisms / 2, 110.5}, {0, 1}]]};

bar[color_, group_] := Graphics[{color, EdgeForm[Thin],
   Rectangle[{parseMito[group][[1, 1]], 101.25}, {parseMito[group][[1, 2]], 104}]}]

mitoTaxomomy = {
   bar[RGBColor[.5, 1, .1], "Alveolata"],
   bar[RGBColor[.2, .2, 0], "Euglenozoa + Amoebozoa"],
   bar[RGBColor[0, .6, .2], "Fungi"],
   bar[RGBColor[.6, .7, .4], "Metazoa + Jakobida"],
   bar[RGBColor[.1, .6, .5], "Viridiplantae + Stramenopiles + Rhodophyta + Rhizaria"],
   Graphics[Text["Metazoa", {
      Mean[parseMito["Metazoa"][[1]]], 105}, {0, -1}]],
   Graphics[Text["1", {
      Mean[parseMito["Alveolata"][[1]]] - 3, 105}, {0, -1}]],
   Graphics[Text["2", {
      Mean[parseMito["Euglenozoa + Amoebozoa"][[1]]] + 3, 105}, {0, -1}]],
   Graphics[Text["3", {
      Mean[parseMito["Fungi"][[1]]], 105}, {0, -1}]],
   Graphics[Text["4", {
      Mean[parseMito["Viridiplantae + Stramenopiles + Rhodophyta + Rhizaria"][[1]]], 105},
     {0, -1}]]};
```

```
f5bc = Show[
  getSelectedAlignment[6, 3, "Anticorrelated", -360, True, 100, 1],
  eigen3Taxomomy[-360, "(b)"],
  mitoRaster ["(c) Mitochondria"],
  mitoTaxomomy,
  BaseStyle → { FontSize → 12, FontFamily → "Courier"},
  ImageSize → 750, AspectRatio → .8]
```

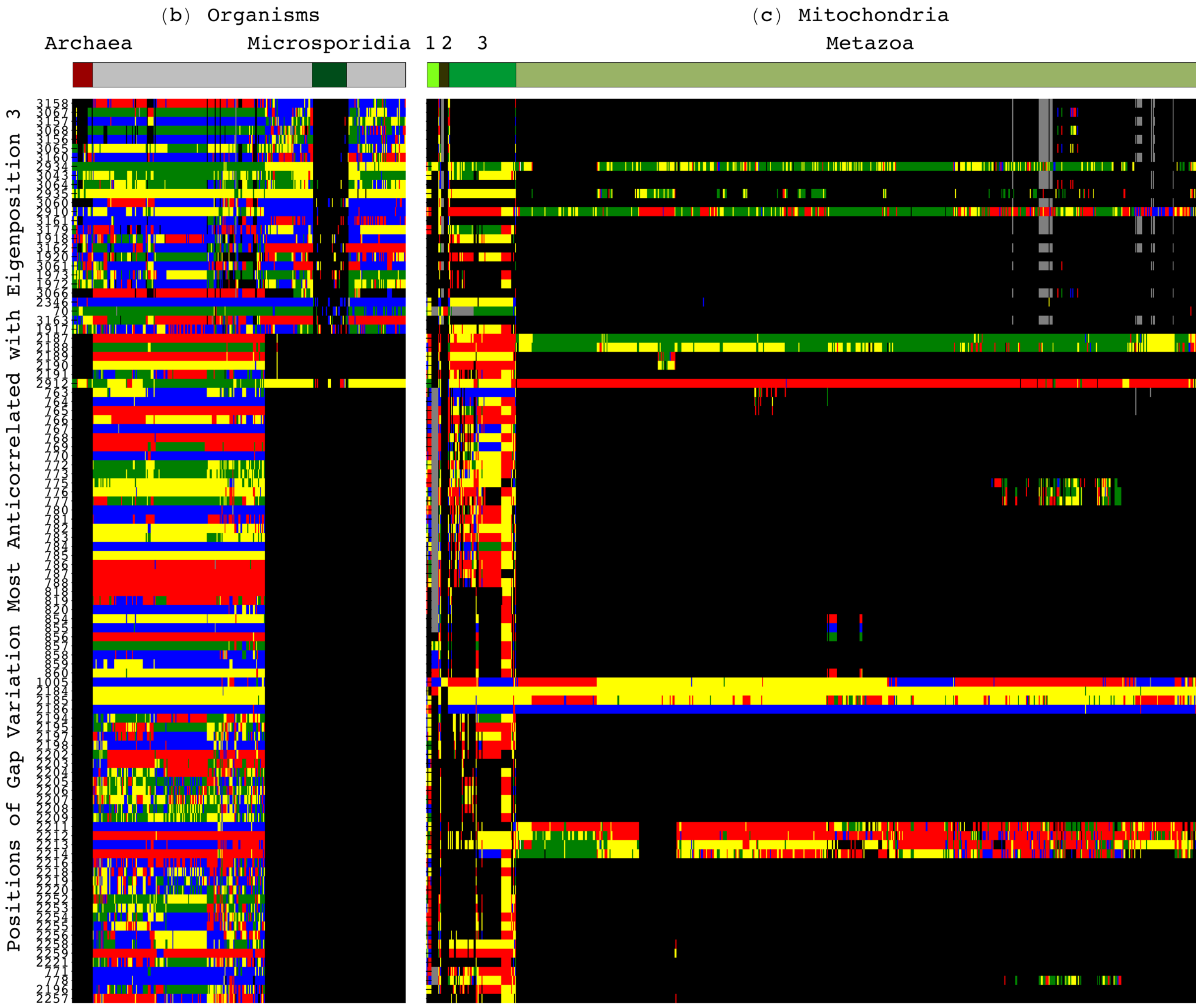

Supplement: Mathematica Notebook S2 — Mode-1 higher-order singular value decomposition (HOSVD) of the 16S rRNA alignment. A PDF format file, readable by Adobe Acrobat Reader. (PDF) [file pone.0018768.s003.pdf]
