# Supplementary material for: Tensor Decomposition Reveals Concurrent Evolutionary Convergences and Divergences and Correlations with Structural Motifs in Ribosomal RNA
Source: PLoS One. 2011 Apr 29;6(4):e18768. doi: 10.1371/journal.pone.0018768 (PMC3094155; doi:10.1371/journal.pone.0018768)

(* © Andrew M. Gross and Orly Alter 2011, All Rights Reserved *)

(* Mode-1 HOSVD of 23S rRNA Sequence Alignment Data from 75 Organisms *)

(* Initialize *)

```
Clear["Global`*"]
organismAnnotations = 2;
positionAnnotations = 1;
```

(* Define Path to Datasets *)

```
path = "Desktop/rRNA/Data/";
```

(* Read Alignment *)

```
stream = path <> "Dataset_S5.txt";
matrix = Import[stream, "Table"];

alignment = Transpose[Drop[ matrix, organismAnnotations, positionAnnotations]];
positionNames = Drop[matrix[[All, positionAnnotations]], organismAnnotations];
organismNames = Drop[matrix[[organismAnnotations]], positionAnnotations];
{organisms, positions} = Dimensions[alignment];
Clear[stream, matrix, matrix1, matrix2];
```

(* Compute Mode-1 HOSVD *)

(* Convert Alignment to Third-Order Tensor *)

```
{vecA, vecC, vecG, vecU, vecN, vecGap} = DiagonalMatrix[Table[1.0, {6}]];
data23S = ReplaceAll[alignment,
   {"A" → vecA, "C" → vecC, "G" → vecG, "U" → vecU, "N" → vecN, "-" → vecGap}];
data23S = Flatten[ Transpose[data23S, {3, 1, 2}], 1];

{eigenorganisms, eigenexpressions, eigenpositions} =
  SingularValueDecomposition[data23S, Min[Dimensions[data23S]]];
eigenexpressions = Diagonal[eigenexpressions];
eigenexpressions = Select[eigenexpressions, # > 0 &];
eigenpositions = Transpose[eigenpositions];
eigenpositions[[2]] = -eigenpositions[[2]];
eigenorganisms[[All, 2]] = -eigenorganisms[[All, 2]];
eigenpositions[[3]] = -eigenpositions[[3]];
eigenorganisms[[All, 3]] = -eigenorganisms[[All, 3]];
eigenpositions[[5]] = -eigenpositions[[5]];
eigenorganisms[[All, 5]] = -eigenorganisms[[All, 5]];
degrees = Dimensions[eigenexpressions]〚1〛;
fractions = eigenexpressions^2 / Sum[eigenexpressions〚a〛^2, {a, 1, degrees}];
entropy = -N[Sum[fractions〚a〛 Log[fractions〚a〛], {a, 1, degrees}] / Log[degrees]];
entropy = N[ Round[100 entropy] / 100]
```

```
0.3
```

```
(* Read Taxonomy Annotations *)

stream = path <> "Dataset_S4.txt";
matrix = Import[stream, "Table"];
taxonomy = Transpose[Drop[matrix, 1, 3]];
Clear[stream, matrix];

(* Define Function to Find All Positions of a Group *)

parse[group_] := {
  regX = StringSplit[group, " + "];
  If[Dimensions[regX][[1]] > 1, {
    groupPositions = {},
    Do[{
      level = Min[Position[taxonomy, regX[[i]]][[All, 1]]],
      groupPositions =
       Union[groupPositions, Flatten[Position[taxonomy[[level]], regX[[i]]]]]
     }, {i, 1, Dimensions[regX][[1]]}]
   },
   {regX = StringSplit[group, " - "],
    If[Dimensions[regX][[1]] > 1, {
      level = Min[Position[taxonomy, regX[[1]]][[All, 1]]],
      groupPositions = Flatten[Position[taxonomy[[level]], regX[[1]]]],
      Do[{
        level = Min[Position[taxonomy, regX[[i]]][[All, 1]]],
        groupPositions =
         Complement[groupPositions, Flatten[Position[taxonomy[[level]], regX[[i]]]]]
       }, {i, 2, Dimensions[regX][[1]]}]
     },
     {level = Min[Position[taxonomy, group][[All, 1]]],
      groupPositions = Flatten[Position[taxonomy[[level]], group]]
     }]}];
  groupPositions
 }
```

```
(* Calculate Enrichment of Significant Eigenpositions in Taxonomic Groups *)

(* Define Cutoffs *)

groupSize = 15;
pValueCutoff = .05;

(* Count Taxonomy Annotations *)

taxonomicGroups = Table[Union[taxonomy[[i]]], {i, 1, 3}];
countTable = Table[{taxonomicGroups[[a, b]], Table[0, {6}, {3}]}, {a, 1,
    Dimensions[taxonomicGroups][[1]]}, {b, 1, Dimensions[taxonomicGroups[[a]]][[1]]}];

Do[{
   annotations = taxonomy[[i]];
   stages = taxonomicGroups[[i]];
   numbers =
    Flatten[Table[{Count[annotations, stages[[a]]]}, {a, 1, Dimensions[stages][[1]]}]];
   annotations = Table[{annotations[[a]]}, {a, 1, organisms}];
   Do[{
     eigenposition = Table[{eigenpositions[[f, a]]}, {a, 1, organisms}],
     pattern = Sort[Join[eigenposition, annotations, 2], OrderedQ[{#2, #1}] &][[All, 2]],
     Do[
      countTable[[i, a, 2, f - 1]] = {
        numbers[[a]],
        Count[pattern[[1 ;; groupSize]], stages[[a]]],
        Count[pattern[[organisms - groupSize + 1 ;; organisms]], stages[[a]]]},
      {a, 1, Dimensions[stages][[1]]}]},
    {f, 2, 7}]},
  {i, 1, 3}];

flatCountTable = Flatten[countTable, 1];
getNumbers[annotation_, eigenposition_] :=
 flatCountTable[[Position[flatCountTable[[All, 1]], annotation][[1, 1]]]][[
  2, eigenposition - 1]]

(* Compute Significance of Eigenposition Association with Taxonomic Groups *)

getEnrichment[group_, eigenposition_, tail_] := {
  regX = StringSplit[group, " + "];
  If[Dimensions[regX][[1]] > 1,
   nums = Sum[getNumbers[regX[[a]], eigenposition], {a, 1, Dimensions[regX][[1]]}],
   {regX = StringSplit[group, " - "],
    If[Dimensions[regX][[1]] > 1,
     nums = getNumbers[regX[[1]], eigenposition] -
       Sum[getNumbers[regX[[a]], eigenposition], {a, 2, Dimensions[regX][[1]]}],
     nums = getNumbers[group, eigenposition]]}];
  If[tail == "Correlated", tailGroup = 2, tailGroup = 3];
  pValue = Sum[N[PDF[HypergeometricDistribution[groupSize, nums[[1]], organisms], k]],
    {k, nums[[tailGroup]], groupSize}];
  {eigenposition, group, nums[[tailGroup]], nums[[1]], ScientificForm[pValue, 2]}
 }
```

```
corrTable =
  acorrTable =
   {{"", "Taxonomic Group", "n", "N", "P-value"}};
corrList = {
   {2, "Eukarya - Microsporidia"},
   {3, "Eukarya - Microsporidia"},
   {4, "Proteobacteria"},
   {5, "Microsporidia"}};
acorrList = {
   {2, "Bacteria"},
   {3, "Microsporidia + Archaea"},
   {4, "Firmicutes"},
   {5, "Archaea"}};
Do[
 corrTable = Append[
   corrTable,
   getEnrichment[corrList[[a, 2]],
     corrList[[a, 1]],
     "Correlated"][[1]]],
 {a, 1, Dimensions[corrList][[1]]}]
Do[
 acorrTable = Append[
   acorrTable,
   getEnrichment[acorrList[[a, 2]],
     acorrList[[a, 1]],
     "Anticorrelated"][[1]]],
 {a, 1, Dimensions[acorrList][[1]]}]
xHeadings = {{
    "", "Correlated", SpanFromLeft, SpanFromLeft,
    SpanFromLeft, "Anticorrelated", SpanFromLeft, SpanFromLeft, SpanFromLeft}};
yHeadings = Transpose[
   {{"", "", Rotate["Eigenposition", Pi / 2], SpanFromAbove, SpanFromAbove, SpanFromAbove}}];
Grid[Join[yHeadings, Join[xHeadings, Join[corrTable, acorrTable[[All, 2 ;; 5]], 2]], 2],
 Alignment → {Left, Bottom}, Spacings → {Scaled[.01], Scaled[.02]}, Frame → All]
```

		Correlated				Anticorrelated			
		Taxonomic Group	n	N	P-value	Taxonomic Group	n	N	P-value
Eigenposition	2	Eukarya - Microsporidia	8	8	3.8×10^{-7}	Bacteria	15	57	9.7×10^{-3}
	3	Eukarya - Microsporidia	8	8	3.8×10^{-7}	Microsporidia + Archaea	10	10	3.6×10^{-9}
	4	Proteobacteria	15	23	2.2×10^{-10}	Firmicutes	12	13	2.2×10^{-10}
	5	Microsporidia	4	4	1.1×10^{-3}	Archaea	6	6	2.5×10^{-5}

```
(* Calculate Enrichment of Motifs Conserved in Taxonomic Groups *)

(* Read Base Pairing *)

stream = path <> "Dataset_S6.txt";
matrix = Import[stream, "Table"];
pairing = Transpose[Drop[matrix, organismAnnotations, positionAnnotations]];
Clear[stream, matrix];

(* Fold Eigenorganisms into a Third-Order Tensor *)

eigenorganismsTensor = Table[eigenorganisms[[Range[a, positions * 6, 6], All]], {a, 1, 6}];
Dimensions[eigenorganismsTensor]

{6, 6636, 75}

(* Define Cutoffs *)

conservationValue1 = 80;
conservationValue2 = 20;
conservationValue3 = 60;
groupSize = 200;
nearZero = .00001;
nucleotides = {"A", "C", "G", "U", "N", "-"};
motifs = {"unpairedA", "unpairedC", "unpairedG", "unpairedU", "unpairedN",
   "helix", "pairedA", "pairedC", "pairedG", "pairedU", "pairedN", "nucGap"};
stages = {"Group", "All"};
allPositions = Range[1, organisms];

(* Calculate Nucleotide and Basepair Frequencies for All Organisms *)

nucleotideCount = Table[Count[alignment[[All, i]], nucleotides[[j]]],
   {i, 1, positions}, {j, 1, Dimensions[nucleotides][[1]]}];
sequenceLength = organisms - nucleotideCount[[All, 6]];
bpCount =
  Table[{Count[pairing[[All, i]], "Y"], Count[pairing[[All, i]], "N"]}, {i, 1, positions}];

ntFrequncyAll = N[(nucleotideCount / organisms) * 100];
bpFrequencyAll = N[(bpCount / sequenceLength) * 100];

(* Calculate Correlation of First Eigenposition and Eigenorganisms with Frequency *)

ones = Table[1, {a, 1, organisms}];
eigenpositions[[1]].(ones / Sqrt[ones.ones])

-0.996065

frequencies = Flatten[ntFrequncyAll];
eigenorganisms[[All, 1]].(frequencies / Sqrt[frequencies.frequencies])

-0.999814
```

```
(* Parse Datasets to Include Only Organisms of Given Group(s) *)

parseAll[group_] := {
  groupPositions = parse[group][[1]];
  restPositions = Complement[allPositions, groupPositions];
  groupAlignment = alignment[[groupPositions]];
  groupBasePairing = pairing[[groupPositions]];
  restAlignment = alignment[[restPositions]];
  restBasePairing = pairing[[restPositions]];
  groupLength = Dimensions[groupPositions][[1]];
  restLength = Dimensions[restPositions][[1]];
  groupNucleotideCount = Table[Count[groupAlignment[[All, i]], nucleotides[[j]]],
    {i, 1, positions}, {j, 1, Dimensions[nucleotides][[1]]}];
  groupLengths = groupLength - groupNucleotideCount[[All, 6]];
  restLengths = sequenceLength - groupLengths;
  restCount = nucleotideCount - groupNucleotideCount;
  groupLengths = Replace[groupLengths, 0 → nearZero, {1}];
  restLengths = Replace[restLengths, 0 → nearZero, {1}];
  ntFrequencyGroup = N[(groupNucleotideCount / groupLength) * 100];
  ntFrequencyRest = N[(restCount / restLength) * 100];
 }

(* Calculate Basepair Frequencies *)

bpFrequencies[] := {
  bpCountGroup = Table[{Count[groupBasePairing[[All, i]], "Y"],
     Count[groupBasePairing[[All, i]], "N"]}, {i, 1, positions}];
  bpCountRest = bpCount - bpCountGroup;
  bpFrequencyGroup = N[(bpCountGroup / groupLengths) * 100];
  bpFrequencyRest = N[(bpCountRest / restLengths) * 100];
 }

(* Calculate Basepair Annotations *)

bpAnnotations[seg_] := {
  bpFrequencies[];
  segment = If[seg > 6, seg - 6, seg];
  paired = unpaired = nuc = Table["-", {positions}];
  Do[{
    If[(ntFrequncyAll[[i, segment]] > conservationValue1),
     {nuc[[i]] = "All",
      If[bpFrequencyAll[[i, 1]] > bpFrequencyAll[[i, 2]],
       paired[[i]] = "All", unpaired[[i]] = "All"]}],
    If[((ntFrequencyGroup[[i, segment]] > conservationValue1) &&
       (ntFrequencyRest[[i, segment]] < conservationValue2)),
     {nuc[[i]] = "Group",
      If[(bpFrequencyGroup[[i, 1]] > bpFrequencyGroup[[i, 2]]),
       paired[[i]] = "Group", unpaired[[i]] = "Group"]}]},
   {i, 1, positions}],
  Switch[segment,
    1, {pairedA = paired, unpairedA = unpaired, nucA = nuc},
    2, {pairedC = paired, unpairedC = unpaired, nucC = nuc},
    3, {pairedG = paired, unpairedG = unpaired, nucG = nuc},
    4, {pairedU = paired, unpairedU = unpaired, nucU = nuc},
    5, {pairedN = paired, unpairedN = unpaired, nucN = nuc},
    6, {pairedGap = paired, unpairedGap = unpaired, nucGap = nuc}];
 }
```

```
(* Calculate Structure Annotations *)

structAnnotations[] := {
  bpFrequencies[];
  helix = Table["-", {positions}];
  annotation = "Group";
  Do[If[(bpFrequencyGroup[[i, 1]] > conservationValue3 &&
      bpFrequencyGroup[[i, 1]] > bpFrequencyRest[[i, 1]]), helix[[i]] = annotation],
   {i, 1, positions}]
 }

(* Get Enrichment of Motifs Conserved in Taxonomic Groups *)

getEnrichments[group_, tail_, eigenorganism_, motif_, segment_, groupSize_] := {
  segmentNumber = Position[nucleotides, segment][[1, 1]];
  motifNumber = Position[motifs, motif][[1, 1]];
  parseAll[group];
  If[motif == "helix", structAnnotations[], bpAnnotations[motifNumber]];
  annotations =
   Switch[motifNumber, 1, unpairedA, 2, unpairedC, 3, unpairedG, 4, unpairedU, 5, pairedN,
    6, helix, 7, pairedA, 8, pairedC, 9, pairedG, 10, pairedU, 11, pairedN, 12, nucGap];
  numbers = Flatten[Table[{Count[annotations, stages[[a]]]},
     {a, 1, Dimensions[stages][[1]]}]];
  annotations = Table[{annotations[[a]]}, {a, 1, positions}];
  pattern =
   Sort[Join[eigenorganismsTensor[[segmentNumber, All, {eigenorganism}]], annotations, 2],
     OrderedQ[{#2, #1}] &][[All, 2]];
  countTable = Table[{
     numbers[[a]],
     Count[Flatten[pattern[[1 ;; groupSize]]], stages[[a]]],
     Count[Flatten[pattern[[positions - groupSize + 1 ;; positions]]], stages[[a]]]},
    {a, 1, Dimensions[stages][[1]]}];
  counts = countTable[[1]];
  If[tail == "Correlated", tailGroup = 2, tailGroup = 3];
  pValue = Sum[N[PDF[HypergeometricDistribution[groupSize, counts[[1]], positions], k]],
    {k, counts[[tailGroup]], groupSize}];
  result = {counts[[tailGroup]], counts[[1]], ScientificForm[pValue, 2]}
 }
```

```
(* Create Formatted Table of Selected Enrichments *)

addTableRow[eigenorganism_, isEigFirst_, segment_, isSegmentFirst_,
  motif_, isMotifFirst_, isCorr_, corrGroup_, isCorrFirst_, isAnticorr_,
  acorrGroup_, isAcorrFirst_, groupSizeCorr_, groupSizeAcorr_] :=
 {head = {If[isEigFirst, eigenorganism, SpanFromAbove],
    If[isSegmentFirst, segment, SpanFromAbove], If[isMotifFirst, motif, SpanFromAbove]};
  corrSection = If[isCorr, Join[If[isCorrFirst, {corrGroup}, {SpanFromAbove}],
     getEnrichments[corrGroup, "Correlated",
       eigenorganism, motif, segment, groupSizeCorr][[1]]],
    If[isCorrFirst, {"", SpanFromLeft, SpanFromLeft, SpanFromLeft},
     {SpanFromAbove, SpanFromBoth, SpanFromBoth, SpanFromBoth}]];
  acorrSection = If[isAnticorr,
    Join[If[isAcorrFirst, {acorrGroup}, {SpanFromAbove}], getEnrichments[acorrGroup,
       "Anticorrelated", eigenorganism, motif, segment, groupSizeAcorr][[1]]],
    If[isAcorrFirst, {"", SpanFromLeft, SpanFromLeft, SpanFromLeft},
     {SpanFromAbove, SpanFromBoth, SpanFromBoth, SpanFromBoth}]];
  result = Join[head, corrSection, acorrSection];
  result
 }

addTableRow[eigenorganism_, isEigFirst_, segment_, isSegmentFirst_, motif_, isMotifFirst_,
  isCorr_, corrGroup_, isCorrFirst_, isAnticorr_, acorrGroup_, isAcorrFirst_] :=
 addTableRow[eigenorganism, isEigFirst, segment, isSegmentFirst,
  motif, isMotifFirst, isCorr, corrGroup, isCorrFirst,
  isAnticorr, acorrGroup, isAcorrFirst, groupSize, groupSize]

enrichmentTable =
  Join[
   {{"", "", "", "Correlated", SpanFromLeft, SpanFromLeft,
     SpanFromLeft, "Anticorrelated", SpanFromLeft, SpanFromLeft, SpanFromLeft}},
   {{"  ", "Tensor \n Slice", "Structure \n Motif", "Taxonomic \n Group",
     "n", "N", "P-value", "Taxonomic \n Group", "n", "N", "P-value"}},
   addTableRow[2, True, "A", True, "unpairedA", True, True,
    "Eukarya - Microsporidia", True, True, "Bacteria", True],
   addTableRow[2, False, "-", True, "nucGap", True, True,
    "Eukarya - Microsporidia", False, True, "Bacteria", False, 200, 91],
   addTableRow[2, False, "-", False, "unpairedA", True, True, "Bacteria",
    True, True, "Eukarya - Microsporidia", True, 200, 91],
   addTableRow[3, True, "A", True, "unpairedA", False, True,
    "Bacteria", False, True, "Archaea + Microsporidia", True],
   addTableRow[3, False, "-", True, "nucGap", True, True, "Bacteria",
    False, True, "Archaea + Microsporidia", False, 200, 100],
   addTableRow[3, False, "-", False, "unpairedA", True, False,
    Null, True, True, "Bacteria", True, 200, 100],
   addTableRow[4, True, "A", True, "unpairedA", False, True,
    "Proteobacteria", True, True, "Firmicutes", True],
   addTableRow[5, True, "A", False, "unpairedA", False, True,
    "Microsporidia", True, True, "Archaea", True],
   addTableRow[5, False, "-", True, "nucGap", True, True, "Microsporidia",
    False, True, "Archaea", False, 200, 199], addTableRow[5, False, "-", False,
    "unpairedA", True, False, Null, True, True, "Microsporidia", True, 200, 199]
  ];
```

```
yHeadings = Transpose[{Join[{""}, {Rotate["Eigenorganism", Pi / 2]},
     Table[SpanFromAbove, {Dimensions[enrichmentTable][[1]] - 2}]]}];

enrichmentTable = ReplaceAll[enrichmentTable, "unpairedA" → "Unpaired A"];
enrichmentTable = ReplaceAll[enrichmentTable, "-" → "Gap"];
enrichmentTable = ReplaceAll[enrichmentTable, "nucGap" → "Gap"]; enrichmentTable =
 ReplaceAll[enrichmentTable, "Eukarya - Microsporidia" → "Eukarya -\n Microsporidia"];
enrichmentTable = ReplaceAll[enrichmentTable,
   "Archaea + Microsporidia" → "Archaea +\n Microsporidia"];

Grid[Join[yHeadings, enrichmentTable, 2],
 Alignment → {Left, Bottom}, Spacings → {Scaled[.01], Scaled[.02]}, Frame → All ]
```

				Correlated				Anticorrelated			
Eigenorganism		Tensor Slice	Structure Motif	Taxonomic Group	n	N	P-value	Taxonomic Group	n	N	P-value
	2	A	Unpaired A	Eukarya - Microsporidia	59	59	1.7×10^{-94}	Bacteria	41	41	6.1×10^{-65}
		Gap	Gap		136	145	2.3×10^{-220}		14	14	2.9×10^{-27}
			Unpaired A	Bacteria	15	41	2.9×10^{-13}	Eukarya - Microsporidia	8	59	1.1×10^{-6}
	3	A			28	41	4.8×10^{-34}	Archaea + Microsporidia	11	11	1.4×10^{-17}
		Gap	Gap		12	14	3.5×10^{-17}		41	45	2.2×10^{-74}
			Unpaired A					Bacteria	8	41	1.3×10^{-7}
	4	A		Proteobacteria	8	8	5.9×10^{-13}	Firmicutes	5	5	2.4×10^{-8}
	5			Microsporidia	16	31	5.1×10^{-17}	Archaea	39	49	6.6×10^{-52}
		Gap	Gap		191	387	3.3×10^{-245}		15	59	1.1×10^{-10}
			Unpaired A					Microsporidia	9	31	1.9×10^{-7}

```
(* Create Raster Display of Sigificant Eigenpositions with Taxonomy Annotation *)
```

```
(* Bar Chart Display of Fractions of Relative Nucleotide Frequency *)

x = 16;
gridx = Table[a, {a, 0, .04, .01}];
framex = Table[{gridx[[a]], Rotate[Round[gridx[[a]], .01], 90 Degree]}, {a, 1, 5}];
framex[[1, 2]] = Rotate["0   ", 90 Degree];
gridx = Table[gridx[[a]], {a, 1, 5}];
framey = Table[{a + 1, x - a - 6}, {a, 0, x - 6 - 1}];
insetBar = BarChart[
   Table[fractions[[x - a]], {a, 6, x - 2}],
   BarOrigin → Left,
   PlotRange → {{0, .04 * 1.0001}, {.5, x - 6 - 1 + .5}},
   Axes → False,
   Frame → True,
   ChartStyle → Red,
   FrameTicks → {None, framey, framex, None},
   GridLines → {gridx, None},
   AspectRatio → 1.1,
   BaseStyle → { FontSize →  11, FontFamily →  "Courier"}];

x = 25;
gridx = Table[a, {a, 0, .8, .2}];
framex = Table[{gridx[[a]], Rotate[Round[gridx[[a]], .05], 90 Degree]}, {a, 1, 5}];
framex[[1, 2]] = Rotate["0    ", 90 Degree];
framey = Table[{a + 1, x - a}, {a, 0, x - 1}];
fractionChart = BarChart[
   Table[fractions[[x - a]], {a, 0, x - 1}],
   BarOrigin → Left,
   PlotRange → {{0, .8 * 1.0001}, {.5, x + .8}},
   ChartStyle → Red,
   Epilog →
    Inset[insetBar, {.16, .25}, {0, 0}, {.65, 22}, {1, 0}, Background → LightYellow],
   Axes → False,
   Frame → True,
   FrameTicks → {None, framey, framex, None},
   GridLines → {gridx, None},
   AspectRatio → 1,
   BaseStyle → { FontSize →  11, FontFamily →  "Courier"}];
```

```
(* Create 2D Red & Green Raster Display of Sigificant Eigenpositions *)

contrast = 5;
displaying = Table[
   adjustedValue = contrast * eigenpositions[[i, j]];
   If[adjustedValue > 0,
    If[adjustedValue < 1,
     {adjustedValue, 0, 0}, {1, 0, 0}],
    If[adjustedValue > -1,
     {0, -adjustedValue, 0}, {0, 1, 0}]],
   {i, 25, 1, -1}, {j, 1, organisms}];
labely = "(b) Eigenpositions";

raster = {
   Graphics[Raster[displaying, {{1, -1}, {organisms + 1, -.02}}]],
   Graphics[Text[labely, {-1.5, -.98 / 2}, {0, -1}, {0, 1}], 11],
   Table[{
     Graphics[Text[a, {.6, -.02 - (.98 / 25) * (a - .4)}, {1, 0}]],
     Graphics[Line[{{1, -.02 - (.98 / 25) * (a - .5)}, {1.5, -.02 - (.98 / 25) * (a - .5)}}]]},
    {a, 1, 25}]
  };

(* Define Functions Used to Generate Color Bars and Line Segments of the Tree *)

Clear[bar, connect, lineUp, addText]

barWidth = .12;

bar[group_, level_, red_, green_, blue_] :=
 Graphics[{RGBColor[red, green, blue],
   EdgeForm[Thickness[.0003]], Rectangle[{Min[parse[group]], level * barWidth - .1},
    {Max[parse[group]] + 1, barWidth + level * barWidth - .1}]}]

bar[group_, altName_, level_, red_, green_, blue_, height_] := {
   bar[group, level, red, green, blue],
   pos = (Min[parse[group]] + Max[parse[group]] + 1) / 2;
   Graphics[Line[{{pos, 2 * barWidth + .06}, {pos, height * .5 + .23}}]],
   Graphics[Style[Text["  " <> altName, {pos, 2 * barWidth + .06}, {1, 0}, {0, -1}],
     Background -> RGBColor[1, 1, 1]]]};

bar[group_, level_, red_, green_, blue_, height_] :=
  bar[group, group, level, red, green, blue, height];

bar[group_, level_, red_, green_, blue__, height_, group2_, height2_] := {
   bar[group, level, red, green, blue, height], addText[group, height2, group2]};

connect[group1_, group2_, height_] :=
  Graphics[Line[{{(Min[parse[group1]] + Max[parse[group1]] + 1) / 2, height * .5 + .23},
     {(Min[parse[group2]] + Max[parse[group2]] + 1) / 2, height * .5 + .23}}]];
```

```
lineUp[group1_, group2_, startHeight_, endHeight_] := {
   pos = (Min[parse[group1]] + Max[parse[group2]] + 1) / 2;
   Graphics[Line[{{pos, startHeight * .5 + .23}, {pos, endHeight * .5 + .23}}]]};

lineUp[group_, startHeight_, endHeight_] := lineUp[group, group, startHeight, endHeight];

addText[group1_, group2_, height_, text_] := Graphics[Style[Text[
     text, {(Min[parse[group1]] + Max[parse[group2]] + 1) / 2 + 1, height * .5 + .25},
     {1, 0}, {0, -1}], Background → RGBColor[1, 1, 1]]];

addText[group_, height_, text_] := addText[group, group, height, text];

(* Create Color Bars and Tree *)

domain = {
   bar["Archaea", 1, .6, 0, 0],
   bar["Bacteria", 1, 0, 0, .6],
   bar["Eukarya", 1, 0, .6, 0]};

{level1, level2} = {1.3, 2};

subgroupings1 = {
   bar["Euryarchaeota", 2, .6, 0, 0, level2],
   bar["Proteobacteria", 2, .2, .15, .25, level1],
   bar["Actinobacteria", 2, .1, .2, .3, level1],
   bar["Firmicutes", 2, .1, .4, .5, level1],
   bar["Tenericutes", 2, .3, .9, 1, level1],
   bar["Spirochaetes+", 2, 0, .2, .6, level1],
   bar["Deinococci", 2, .2, 0, .8, level1],
   bar["Chlamydiae+", 2, 0, .7, 1, level1],
   bar["Apicomplexa+", 2, .4, .6, 0, level1],
   bar["Microsporidia", 2, 0, .3, .1, level1],
   bar["Viridiplantae", 2, .1, .6, .5, level1],
   connect["Proteobacteria", "Chlamydiae+", level1],
   connect["Apicomplexa+", "Viridiplantae", level1],
   lineUp["Bacteria", level1, level2],
   lineUp["Eukarya", level1, level2],
   connect["Archaea", "Eukarya", level2]};

text = {
   addText["Bacteria", level1, "Bacteria"],
   addText["Archaea", level1, "Archaea"],
   addText["Eukarya", level1, "Eukarya"]
  };
```

(* Show Sigificant Eigenpositions with Annotation *)

```
s2 = Show[{raster, subgroupings1, domain,
    text, Graphics[{Opacity[0], Rectangle[{0, 0}, {108, -1}]}],
    Graphics[Text["(c) Fraction of\nNucleotide Frequency", {87.5, .20}, {0, 0}]],
    Graphics[Text["(a) Organisms", {organisms / 2.3, 1.28}, {-1, 0}]]
  },
  Epilog → Inset[fractionChart, {79, -1.025}, {0, 0}, {20, 10}],
  AspectRatio → .4,
  BaseStyle → { FontSize → 11, FontFamily → "Courier"},
  ImageSize → 1150]
```

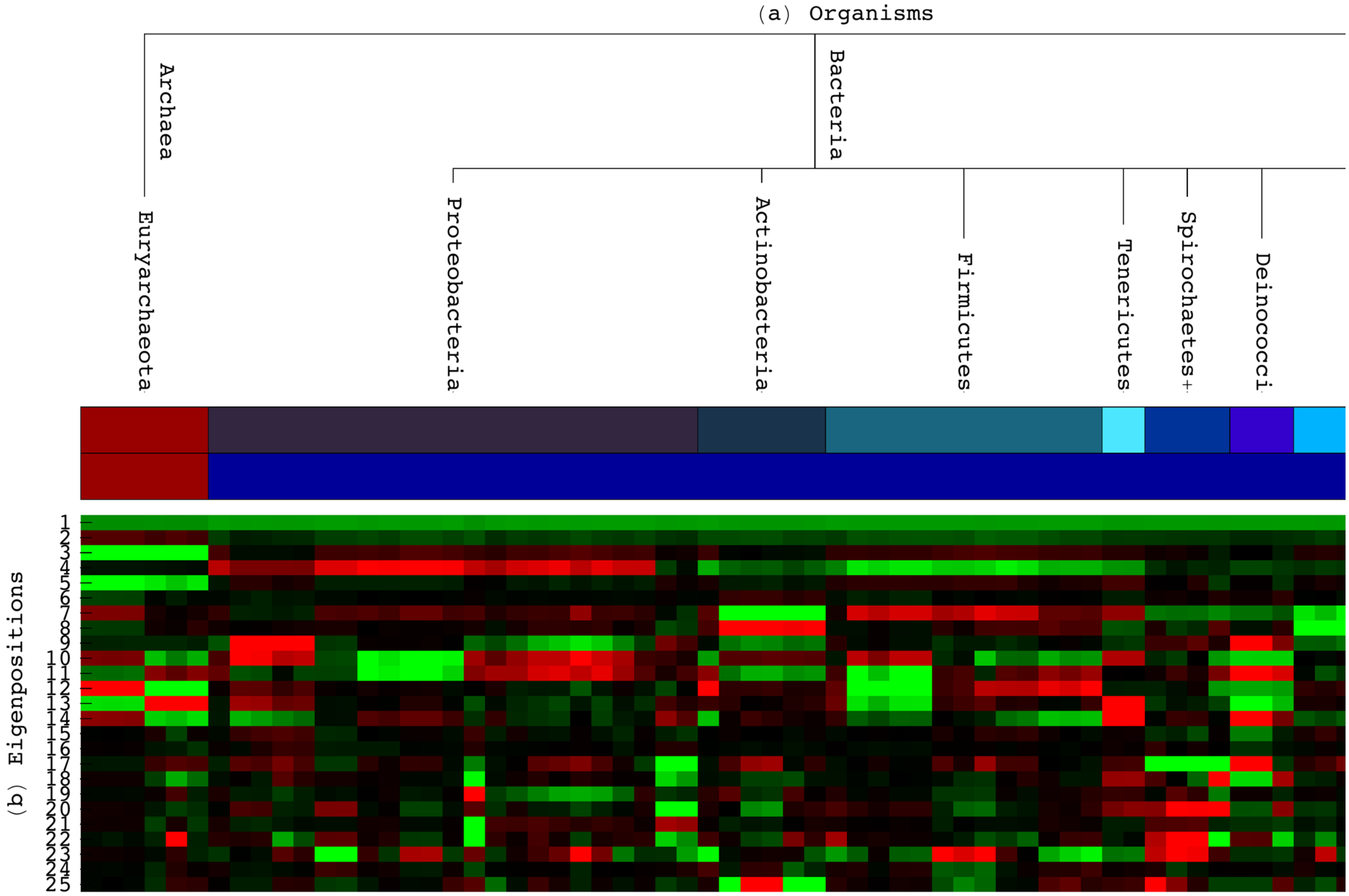

```
(* Create Eigenpositions Line Graph Display *)

(* Define Constants and Functions for Creating Color Bars and Dashed Lines *)

{topBar, bottomBar} = {.45, -.495};
barHeight = .045;

bar2[group_, color_, position_] := Graphics[
   {color, EdgeForm[Thickness[.0005]], Rectangle[{Min[parse[group]], position + offset},
     {Max[parse[group]] + 1, position + barHeight + offset}]}];

dottedLines[groupList_, offset_] := {
  lineList = {};
  Do[lineList =
    Union[lineList, {Min[parse[groupList[[a]]]], Max[parse[groupList[[a]]]] + 1}],
   {a, 1, Dimensions[groupList][[1]]}];
  lineList = Complement[lineList, {1, organisms + 1}];
  lineList = Table[Graphics[{
       Thickness[Medium], Dashed, Opacity[.7],
       Line[{{lineList[[a]], .45 + offset}, {lineList[[a]], -.45 + offset}}]}],
    {a, 1, Dimensions[lineList][[1]]}];
  Show[lineList,
   PlotRange → {{1, 75}, {-.6 + offset, .6 + offset}},
   AspectRatio → 1,
   Frame → True]
 }

createPlots[coordinates1_, coordinates2_, label_, offset_] := {
  points1 = Table[Point[coordinates1[[a]]], {a, 1, organisms}];
  points2 = Table[Point[coordinates2[[a]]], {a, 1, organisms}];
  Show[
   Graphics[{Red, Thickness[.001], Line[coordinates1], PointSize[.007], points1}],
   Graphics[{Blue, Thickness[.001], Line[coordinates2], PointSize[.007], points2}],
   Graphics[Line[{{1, .45 + offset}, {1, -.45 + offset},
      {organisms + 1, -.45 + offset}, {organisms + 1, .45 + offset}}]],
   Graphics[Line[{{1, 0 + offset}, {organisms + 1, 0 + offset}}]],
   Graphics[Text["0.3", {0, .3 + offset}, {1, 0}]],
   Graphics[Text["-0.3", {0, -.3 + offset}, {1, 0}]],
   Graphics[Line[{{1, .3 + offset}, {.5, .3 + offset}}]],
   Graphics[Line[{{1, -.3 + offset}, {.5, -.3 + offset}}]],
   Graphics[Text[label, {-.5, topBar + 1.5 * barHeight + offset}, {1, 1}]]]
 }
```

```
(* Create Eigenpositions 2 and 4 Plots *)

offset = 1.26;

domain = {
   bar2["Archaea + Bacteria + Eukarya", RGBColor[.75, .75, .75], topBar],
   bar2["Eukarya", RGBColor[0, .6, 0], topBar],
   bar2["Microsporidia", RGBColor[.75, .75, .75], topBar],
   bar2["Bacteria", RGBColor[0, 0, .5], topBar]};

subgroupings1 = {
   bar2["Archaea + Bacteria + Eukarya", RGBColor[.75, .75, .75], bottomBar],
   bar2["Firmicutes", RGBColor[.1, .4, .5], bottomBar],
   bar2["Proteobacteria", RGBColor[.2, .15, .25], bottomBar]};

lines = dottedLines[
   {"Bacteria", "Eukarya", "Microsporidia", "Firmicutes", "Proteobacteria"}, offset];

label = "(a)";
coordinates1 = Table[{a + .5, eigenpositions[[2, a]] + offset}, {a, 1, organisms}];
coordinates2 = Table[{a + .5, eigenpositions[[4, a]] + offset}, {a, 1, organisms}];
g = createPlots[coordinates1, coordinates2, label, offset];
eig2and3 = Show[domain, subgroupings1, lines, g];

(* Create Eigenpositions 3 and 5 Plots *)

offset = .22;

domain = {
   bar2["Archaea + Bacteria + Eukarya", RGBColor[.75, .75, .75], topBar],
   bar2["Microsporidia", RGBColor[0, .3, .1], topBar],
   bar2["Archaea", RGBColor[.6, 0, 0], topBar]
  };

subgroupings1 = {
   bar2["Archaea + Bacteria + Eukarya", RGBColor[.75, .75, .75], bottomBar],
   bar2["Archaea", RGBColor[.6, 0, 0], bottomBar],
   bar2["Microsporidia", RGBColor[0, .3, .1], bottomBar]};

lines = dottedLines[{"Archaea", "Microsporidia"}, offset];

label = "(b)";
coordinates1 = Table[{a + .5, eigenpositions[[3, a]] + offset}, {a, 1, organisms}];
coordinates2 = Table[{a + .5, eigenpositions[[5, a]] + offset}, {a, 1, organisms}];
g = createPlots[coordinates1, coordinates2, label, offset];
eig4and5 = Show[domain, subgroupings1, lines, g];
```

```
(* Define Functions to Generate Color Bars and Line Segments of the Tree *)

yOffset = 1.75;
{level1, level2} = {.6, .95};

bar[group_, level_, red_, green_, blue_] :=
 Graphics[{RGBColor[red, green, blue], EdgeForm[Thickness[.0005]],
   Rectangle[{Min[parse[group]], level * barHeight + yOffset},
    {Max[parse[group]] + 1, barHeight + level * barHeight + yOffset}]}]

bar[group_, level_, red_, green_, blue_, height_] := {
  bar[group, level, red, green, blue],
  lineUp[group, .025, height]}

bar[group_, level_, red_, green_, blue_, height_, name2_, height2_] :=
  bar[group, level, red, green, blue, height];

connect[group1_, group2_, height_] := Graphics[Line[
   {{(Min[parse[group1]] + Max[parse[group1]] + 1) / 2, height + barHeight * 3 + yOffset},
    {(Min[parse[group2]] + Max[parse[group2]] + 1) / 2, height + barHeight * 3 + yOffset}}]]

lineUp[group1_, group2_, startHeight_, endHeight_] := {
  pos = ( Min[parse[group1]] + Max[parse[group2]] + 1) / 2;
  Graphics[Line[
    {{pos, startHeight + barHeight * 3 + yOffset}, {pos, endHeight + barHeight * 3 + yOffset}}]]}

lineUp[group_, startHeight_, endHeight_] := lineUp[group, group, startHeight, endHeight];

bar[group_, altName_, level_, red_, green_, blue_, height_] :=
 {bar[group, level, red, green, blue], lineUp[group, .025, height]}

bar[group_, altName_, level_, red_, green_, blue_, height_, name2_, height2_] :=
  bar[group, level, red, green, blue, height, name2, height2];

addText[group_, height_, text_] := Graphics[Text[text,
   {(Min[parse[group]] + Max[parse[group]] + 1) / 2 + 1,
    height + barHeight * 3 + yOffset + .03}, {1, 0}, {0, -1}]]

addText[group_, height_, text_, flag_] := Graphics[Style[Text[" " <> text,
    {(Min[parse[group]] + Max[parse[group]] + 1) / 2 + .5, height + barHeight * 3 + yOffset},
    {1, 0}, {0, -1}], Background → RGBColor[1, 1, 1]]]
```

```
(* Create Color Bars and Tree *)

domain = {
   bar["Archaea", 1, .6, 0, 0],
   bar["Bacteria", 1, 0, 0, .6],
   bar["Eukarya", 1, 0, .6, 0]};

subgroupings1 = {
   bar["Euryarchaeota", 2, .6, 0, 0, level2],
   bar["Proteobacteria", 2, .2, .15, .25, level1],
   bar["Actinobacteria", 2, .1, .2, .3, level1],
   bar["Firmicutes", 2, .1, .4, .5, level1],
   bar["Tenericutes", 2, .3, .9, 1, level1],
   bar["Spirochaetes+", 2, 0, .2, .6, level1],
   bar["Deinococci", 2, .2, 0, .8, level1],
   bar["Chlamydiae+", 2, 0, .7, 1, level1],
   bar["Apicomplexa+", 2, .4, .6, 0, level1],
   bar["Microsporidia", 2, 0, .3, .1, level1],
   bar["Viridiplantae", 2, .1, .6, .5, level1],
   connect["Proteobacteria", "Chlamydiae+", level1],
   connect["Apicomplexa+", "Viridiplantae", level1],
   lineUp["Bacteria", level1, level2],
   lineUp["Eukarya", level1, level2],
   connect["Archaea", "Eukarya", level2]};

text = {
   addText["Bacteria", level1, "Bacteria"],
   addText["Archaea", level1, "Archaea"],
   addText["Eukarya", level1, "Eukarya"],
   addText["Proteobacteria", 0, "Proteobacteria"],
   addText["Microsporidia", 0, "Microsporidia"],
   addText["Firmicutes", 0, "Firmicutes"]
  };
```

```
(* Show Eigenpositions Line Graph Display *)

s3 = Show[{eig2and3, eig4and5, domain, subgroupings1, text,
    Graphics[{Yellow, Opacity[0], Rectangle[{organisms, 1.47}, {organisms + 8, -.225}]}],
    Graphics[Text["Organisms", {organisms / 2, 2.9}, {1, 0}]],
    Graphics[Text["Eigenposition", {organisms + 5, .53}, {1, 0}, {0, -1}]],
    Graphics[Text["-2", {organisms + 3, 1.735}, {1, 0}]],
    Graphics[Text["-4", {organisms + 3, .795}, {1, 0}]],
    Graphics[Text["-3", {organisms + 3, .695}, {1, 0}]],
    Graphics[Text["-5", {organisms + 3, -.245}, {1, 0}]],
    Graphics[Text["Relative Nucletide Frequency", {-6, .68}, {0, 0}, {0, 1}]]},
  AspectRatio → 1, ImageSize → 550, BaseStyle → { FontSize → 10, FontFamily → "Courier"}]
```

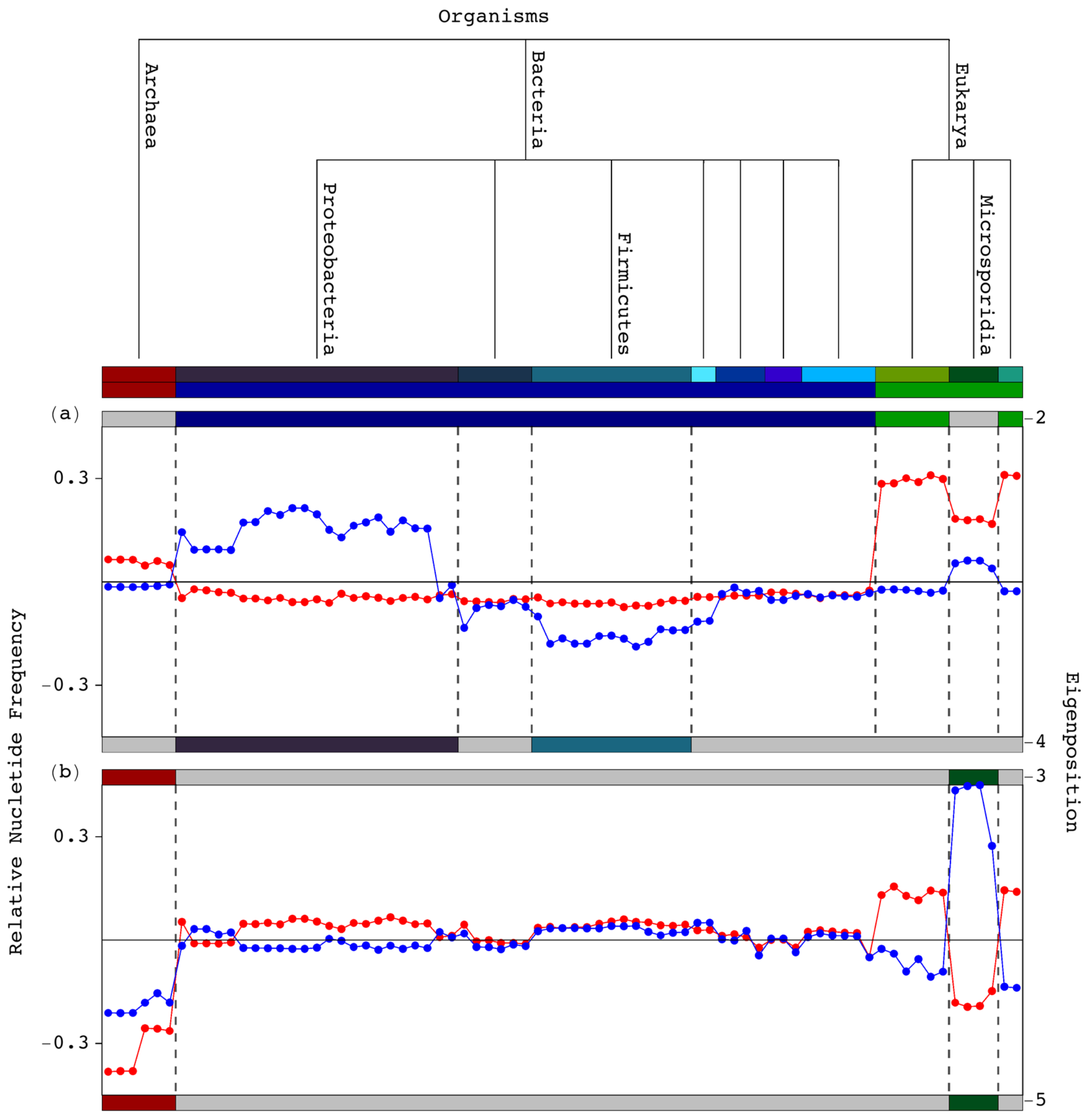

```
(* Create Raster Displays for Selected Positions of the Alignment *)

(* Define Function to Generate Raster *)

segmentMap = {"A", "C", "G", "U", "N", "Gap"};

getSelectedAlignment[segment_, pattern_, tail_,
  offset_, showPositions_, clusterSize_, mitoOffset_] := {
  selectedVector = eigenorganisms[[Range[segment, positions * 6, 6], pattern]];
  posList = If[tail == "Anticorrelated",
    Range[clusterSize, 1, -1], Range[positions - clusterSize + 1, positions]];
  selectedPositions = Sort[Table[{a, selectedVector[[a]]}, {a, 1, positions}],
     OrderedQ[{{#1[[2]]}, {#2[[2]]}}] &][[posList, 1]];
  selectedAlignment = Transpose[alignment[[All, selectedPositions]]];
  selectedAlignment =
   ReplaceAll[selectedAlignment, {"A" → 1, "C" → 2, "G" → 3, "U" → 4, "N" → 5, "-" → 6}];
  If[showPositions, {
    yLabelPosition = -8 + mitoOffset;
    positionLabels = {Table[Graphics[
        Text[Style[selectedPositions[[a]], 8], {-1 + offset, 100 - clusterSize + a - .5}, {1, 0}]
       {a, 1, clusterSize}],
      Table[Graphics[Line[{{-.75 + offset, 100 - clusterSize + a - .5},
          {0 + offset, 100 - clusterSize + a - .5}}]],
       {a, 1, clusterSize}]}}, yLabelPosition = -5 - 7.5 * mitoOffset];
  outputRaster = {
    Graphics[
     Raster[selectedAlignment, {{offset, 100 - clusterSize}, {offset + organisms, 100}},
      ColorFunction → (Switch[#, 1, Red, 2, RGBColor[0, .5, 0], 3, Blue, 4,
          Yellow, 5, RGBColor[.5, .5, .5], 6, Black] &)], AspectRatio → 2],
    Graphics[Text["Positions of " <> segmentMap[[segment]] <> " Variation Most " <>
       tail <> " with Eigenposition " <> ToString[pattern],
      {yLabelPosition + offset, 99}, {1, 0}, {0, 1}]]};
  If[showPositions, outputRaster = {outputRaster, positionLabels}];
  outputRaster
 }

Clear[yLabelPosition]
```

```
(* Define Taxonomic Grouping Bars for Selected Eigenpositions *)

bar[group_, color_, offset_] := Graphics[{color, EdgeForm[Thin],
    Rectangle[{Min[parse[group]] + offset, 101.25}, {Max[parse[group]] + 1 + offset, 105}]}];

eigen2Taxomomy[offset_, label_] := {
   bar["Archaea + Bacteria + Eukarya", RGBColor[.75, .75, .75], offset],
   bar["Eukarya", RGBColor[0, .6, 0], offset],
   bar["Microsporidia", RGBColor[.75, .75, .75], offset],
   bar["Bacteria", RGBColor[0, 0, .5], offset],
   Graphics[Text["Bacteria", {Mean[parse["Bacteria"][[1]]] + offset, 106}, {0, -1}]],
   Graphics[Text["Eukarya-", {Mean[parse["Eukarya"][[1]]] + offset, 108}, {0, -1}]],
   Graphics[Text["Microsporidia", {Mean[parse["Eukarya"][[1]]] + offset, 106}, {0, -1}]],
   Graphics[Text[label <> " Organisms", {organisms / 2 + offset, 113}, {0, 1}]]};

eigen3Taxomomy[offset_, label_] := {
   bar["Archaea + Bacteria + Eukarya", RGBColor[.75, .75, .75], offset],
   bar["Archaea", RGBColor[.6, 0, 0], offset],
   bar["Microsporidia", RGBColor[0, .3, .1], offset],
   Graphics[Text["Archaea", {Mean[parse["Archaea"][[1]]] + offset, 106}, {0, -1}]],
   Graphics[
    Text["Microsporidia", {Mean[parse["Microsporidia"][[1]]] + offset, 106}, {0, -1}]],
   Graphics[Text[label <> " Organisms", {organisms / 2 + offset, 113}, {0, 1}]]};

eigen4Taxomomy[offset_, label_] := {
   bar["Archaea + Bacteria + Eukarya", RGBColor[.75, .75, .75], offset],
   bar["Firmicutes", RGBColor[.1, .4, .5], offset],
   bar["Proteobacteria", RGBColor[.2, .15, .25], offset],
   Graphics[Text["Firmicutes", {Mean[parse["Firmicutes"][[1]]] + offset, 106}, {0, -1}]],
   Graphics[
    Text["Proteobacteria", {Mean[parse["Proteobacteria"][[1]]] + offset, 106}, {0, -1}]],
   Graphics[Text[label <> " Organisms", {organisms / 2 + offset, 113}, {0, 1}]]};

eigen5Taxomomy[offset_, label_] := eigen3Taxomomy[offset, label];
```

```
(* Show Rasters of Positions with Greatest Relative Nucleotide Frequency Variation *)

s6 = Show[
  eigen2Taxomomy[-1, "(a)"],
  eigen2Taxomomy[89, "(b)"],
  getSelectedAlignment[6, 2, "Correlated", 0, True, 200, 0][[1]],
  getSelectedAlignment[6, 2, "Anticorrelated", 90, True, 91, 0][[1]],
  BaseStyle → {FontSize → 10, FontFamily → "Courier"},
  AspectRatio → 1.5, ImageSize → 700]
```

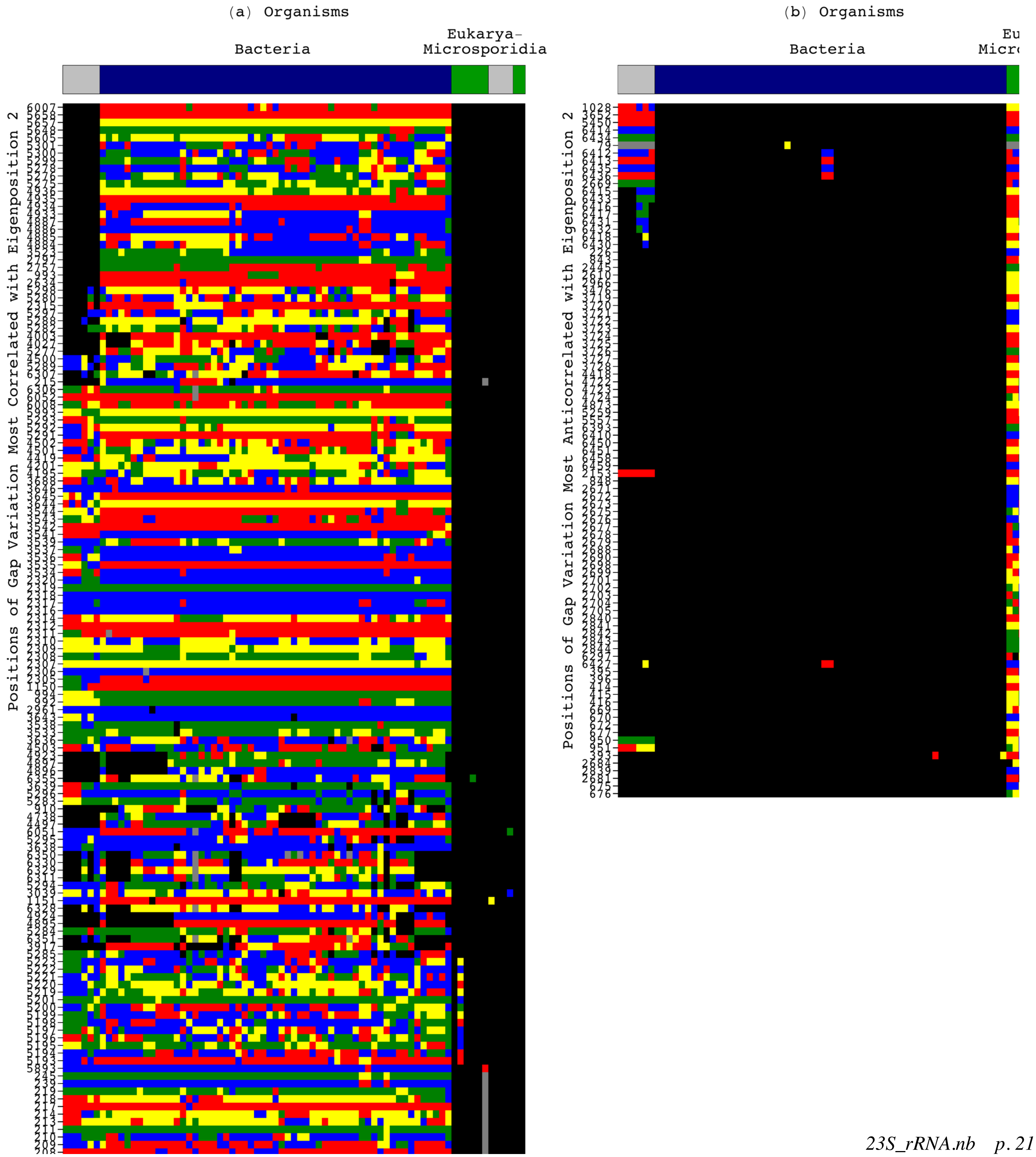

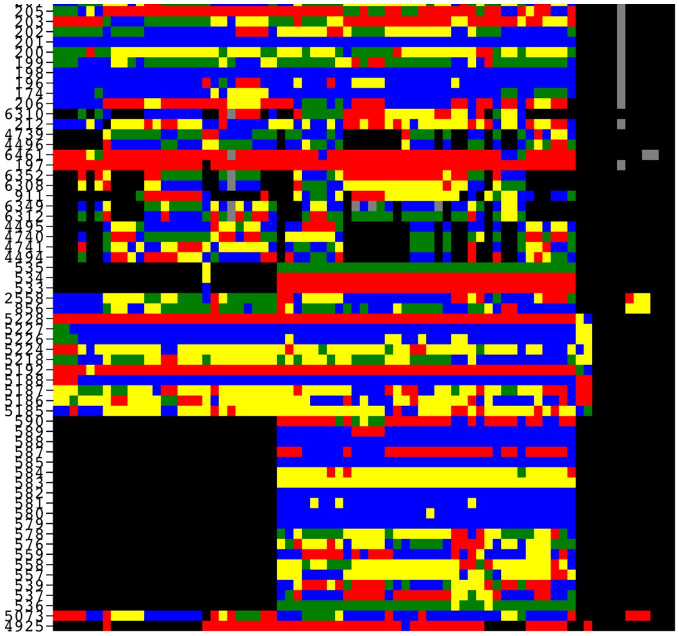

205
203
202
201
200
199
198
196
174
206
6310
212
4739
4496
6461
197
6352
6308
911
6349
6312
4495
4740
4741
4494
535
534
533
2558
856
5228
5227
5226
5224
5218
5192
5188
5187
5186
5185
590
589
588
587
585
584
583
582
581
580
579
578
576
559
558
557
539
537
536
5073
4925

```
s9 = Show[
  eigen2Taxomomy[-1, "(a)"],
  eigen2Taxomomy[89, "(b)"],
  getSelectedAlignment[1, 2, "Correlated", 0, True, 200, 0][[1]],
  getSelectedAlignment[1, 2, "Anticorrelated", 90, True, 200, 0][[1]],
  BaseStyle → {FontSize → 10, FontFamily → "Courier"},
  AspectRatio → 1.5, ImageSize → 700]
```

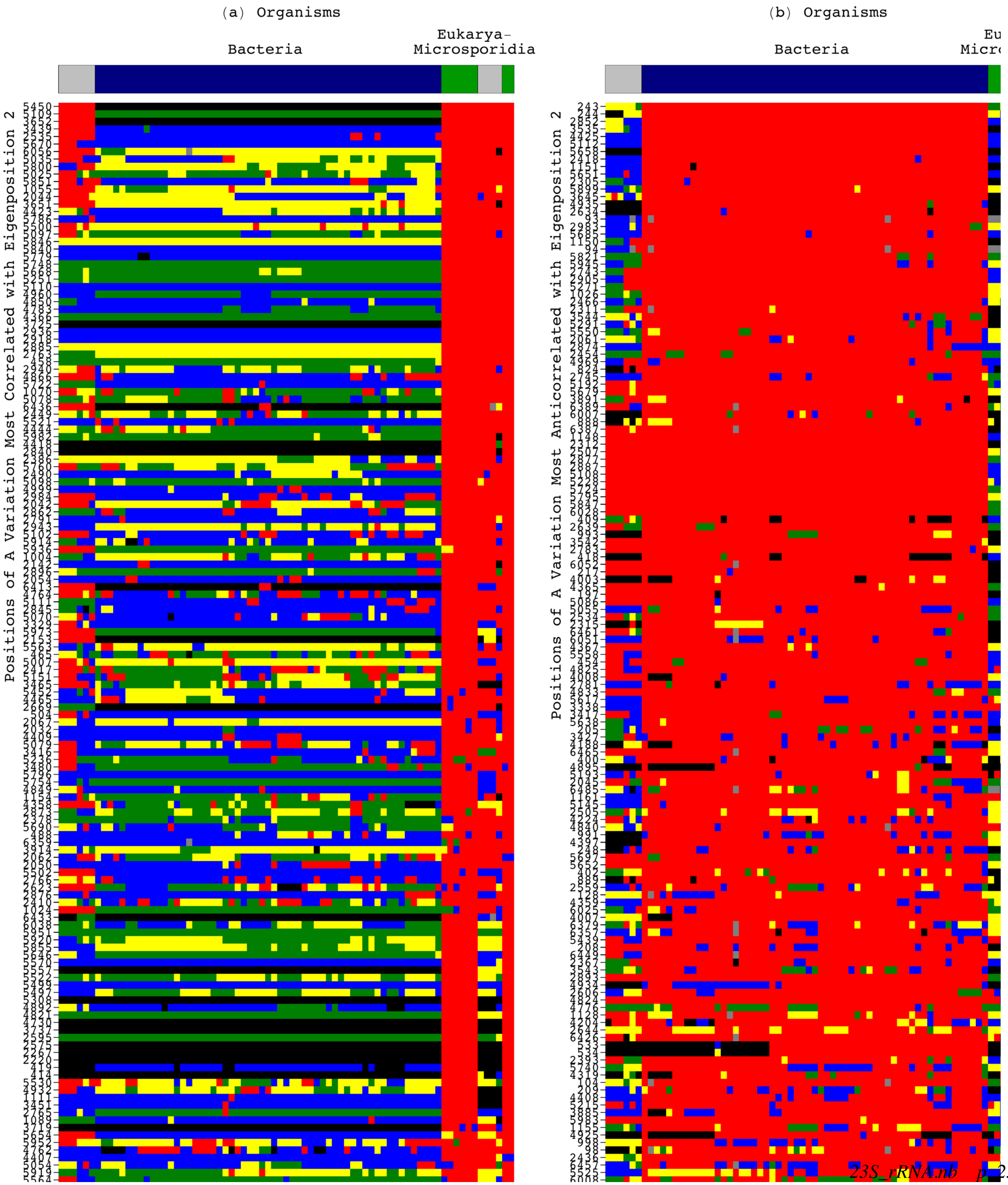

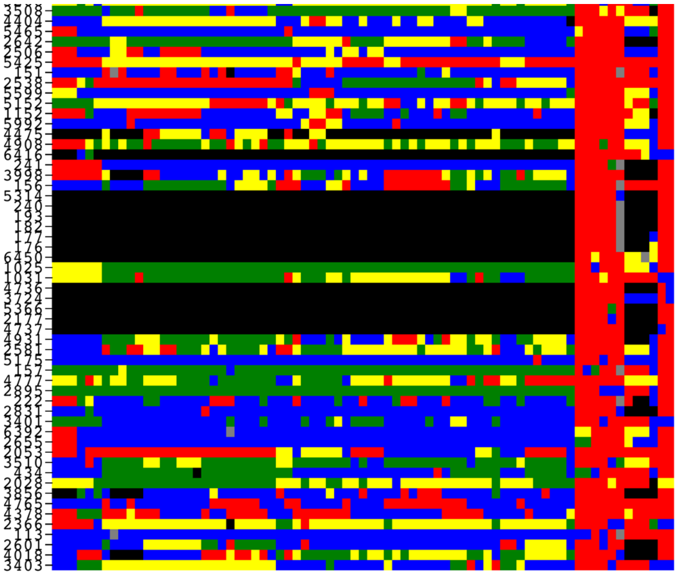

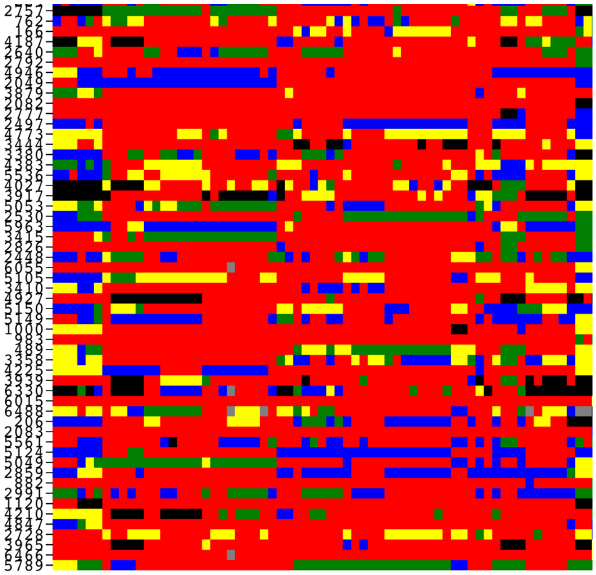

```
s10 = Show[
  eigen3Taxomomy[-1, "(a)"],
  eigen3Taxomomy[89, "(b)"],
  getSelectedAlignment[6, 3, "Correlated", 0, True, 200, 0][[1]],
  getSelectedAlignment[6, 3, "Anticorrelated", 90, True, 100, 0][[1]],
  BaseStyle → {FontSize → 10, FontFamily → "Courier"},
  AspectRatio → 1.5, ImageSize → 700]
```

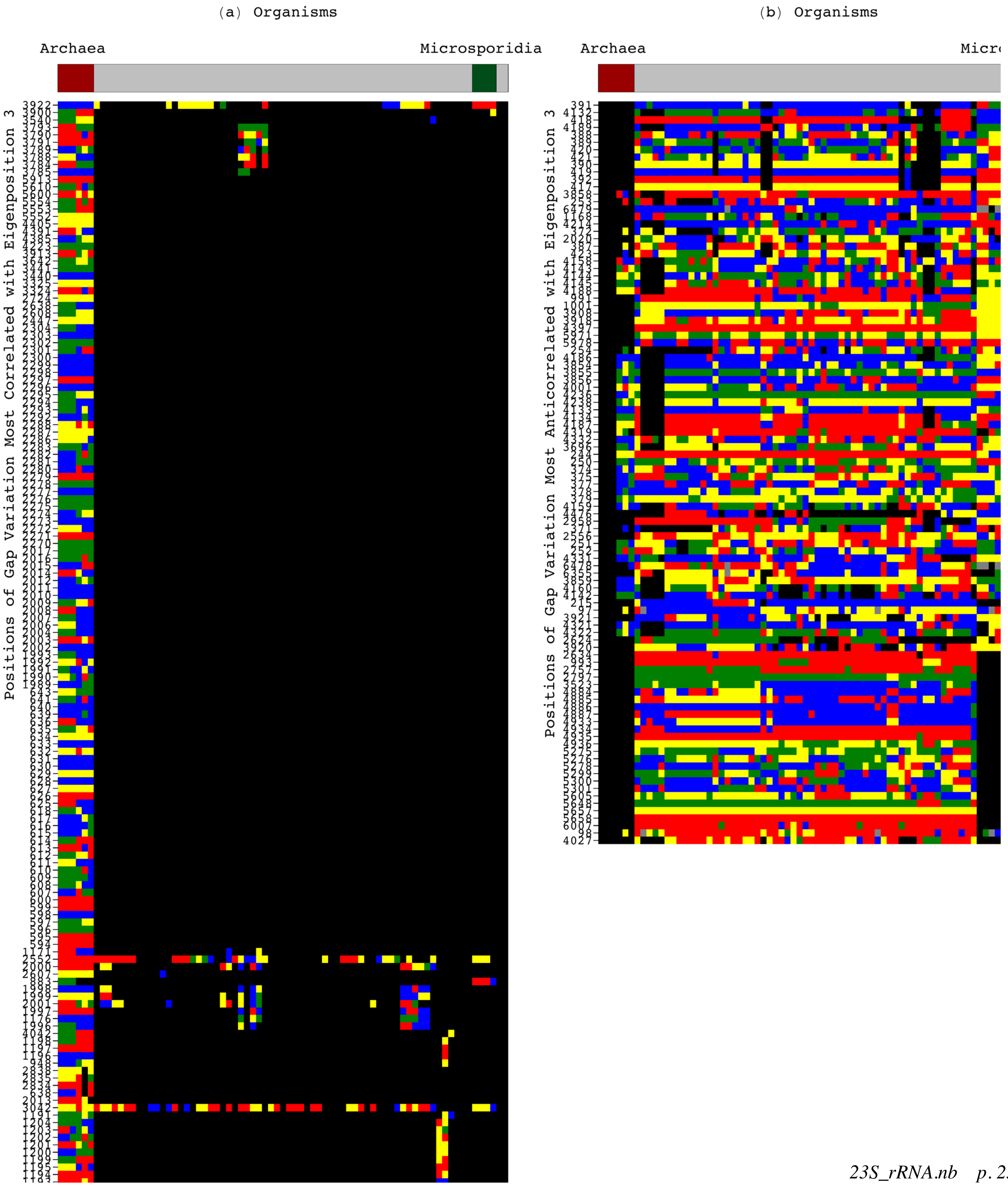

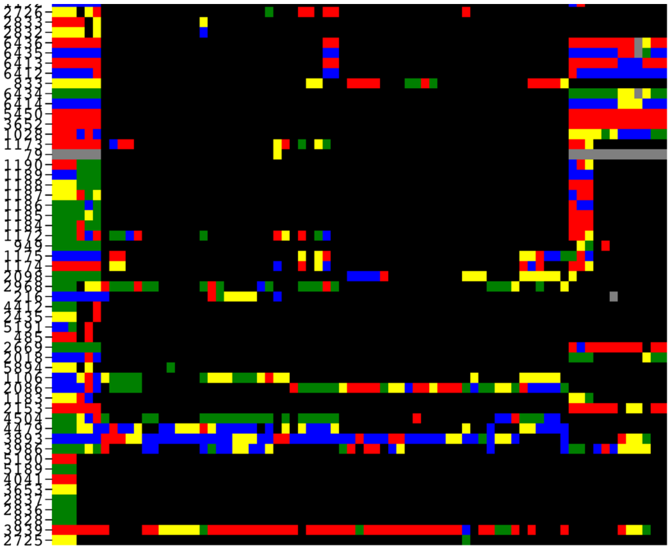
2726
2833
2832
6436
6435
6413
6412
833
6434
6414
5450
3652
1028
1173
79
1190
1189
1188
1187
1186
1185
1184
1172
949
1175
1174
2098
2968
216
4412
2435
5191
485
2669
2018
5894
1106
2086
1183
2153
4504
4479
3893
3986
5190
5189
4041
3653
2837
2836
828
3939
2725

```
s11 = Show[
  eigen3Taxomomy[-1, "(a)"],
  eigen3Taxomomy[89, "(b)"],
  getSelectedAlignment[1, 3, "Correlated", 0, True, 200, 0][[1]],
  getSelectedAlignment[1, 3, "Anticorrelated", 90, True, 200, 0][[1]],
  BaseStyle → { FontSize → 10, FontFamily → "Courier"},
  AspectRatio → 1.5, ImageSize → 700]
```

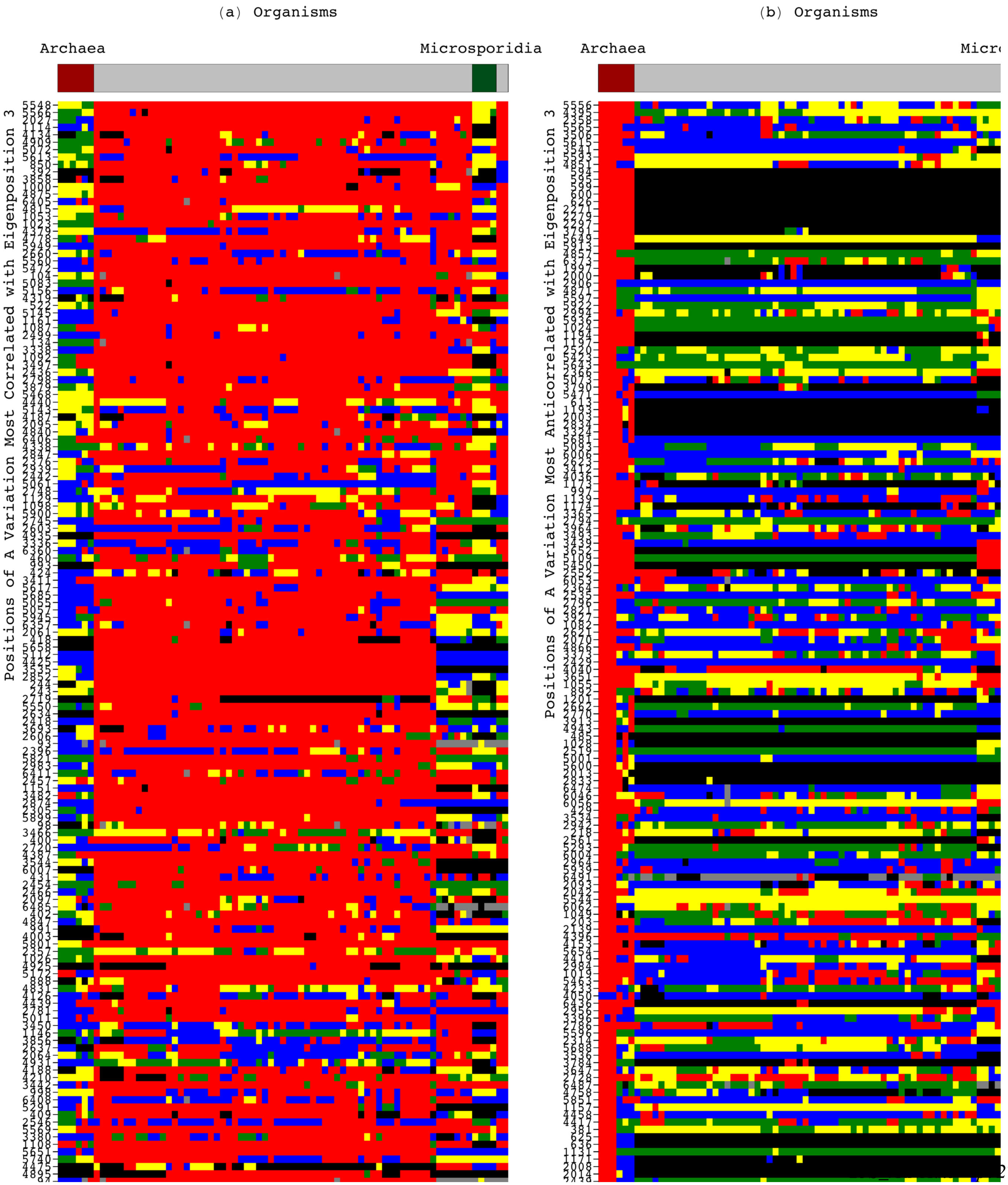

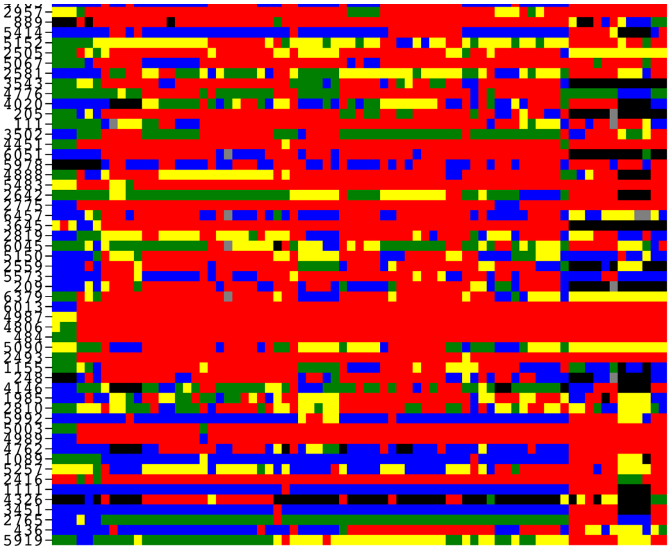

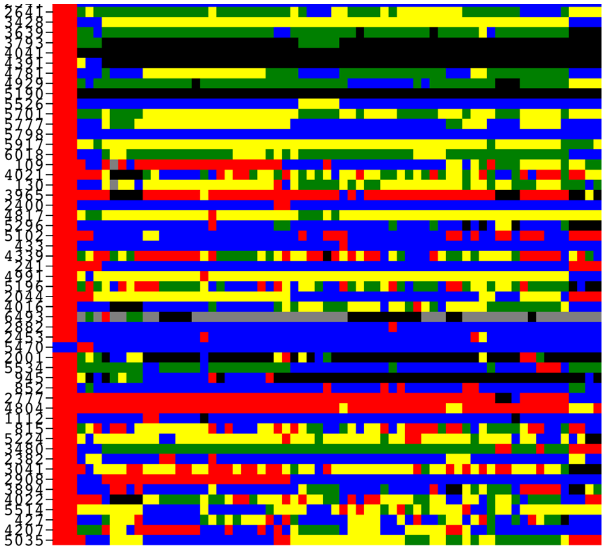

```
s16 = Show[
  eigen5Taxomomy[-1, "(a)"],
  eigen5Taxomomy[89, "(b)"],
  getSelectedAlignment[6, 5, "Correlated", 0, True, 200, 0][[1]],
  getSelectedAlignment[6, 5, "Anticorrelated", 90, True, 199, 0][[1]],
  BaseStyle → { FontSize → 10, FontFamily → "Courier"},
  AspectRatio → 1.5, ImageSize → 700]
```

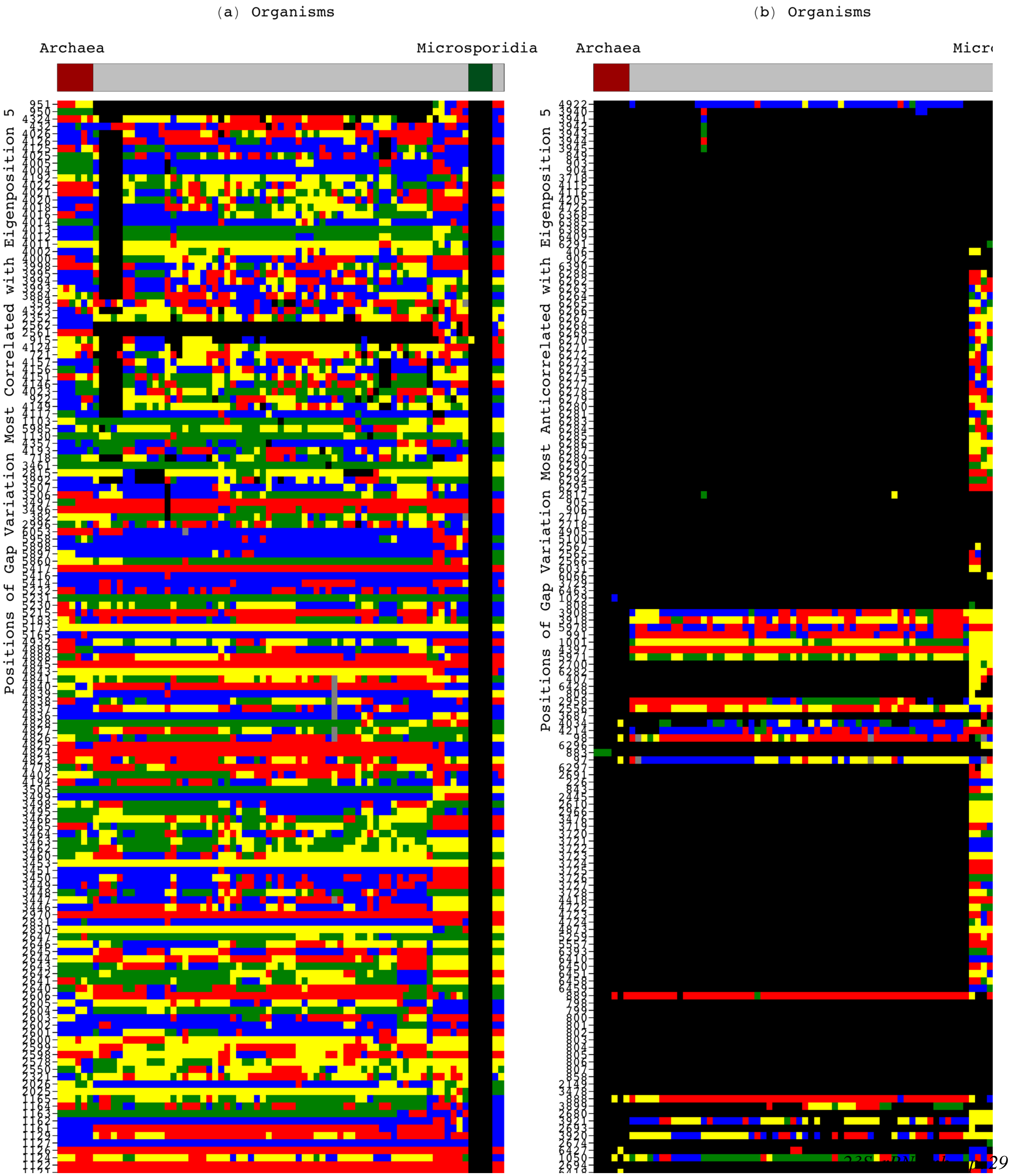

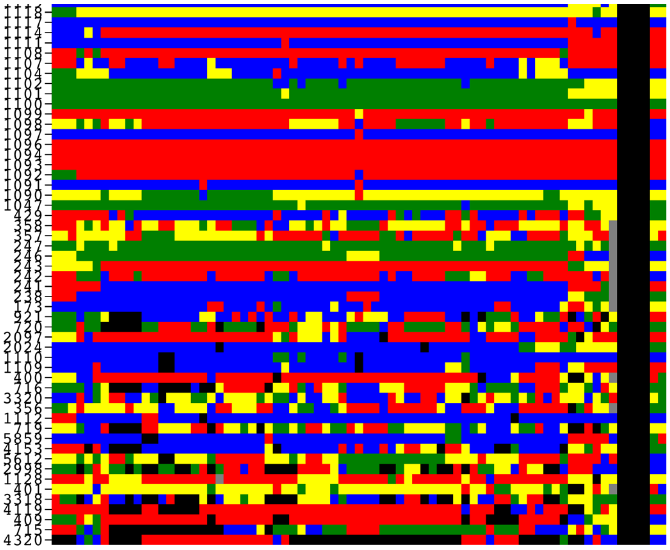

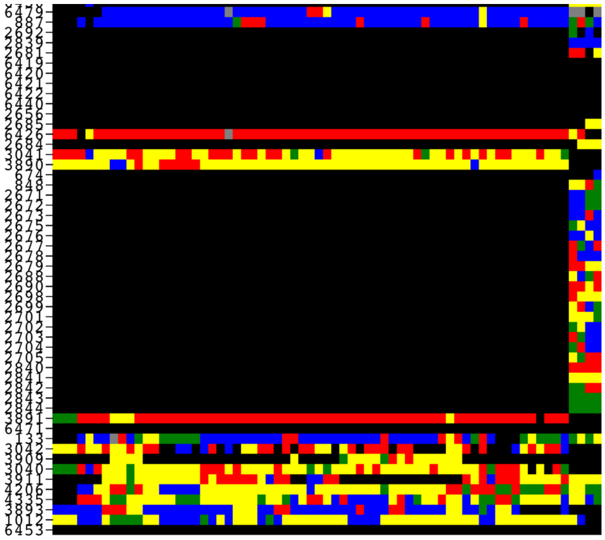

```
s17 = Show[
  eigen5Taxomomy[-1, "(a)"],
  eigen5Taxomomy[89, "(b)"],
  getSelectedAlignment[1, 5, "Correlated", 0, True, 200, 0][[1]],
  getSelectedAlignment[1, 5, "Anticorrelated", 90, True, 200, 0][[1]],
  BaseStyle → { FontSize → 10, FontFamily → "Courier"},
  AspectRatio → 1.5, ImageSize → 700]
```

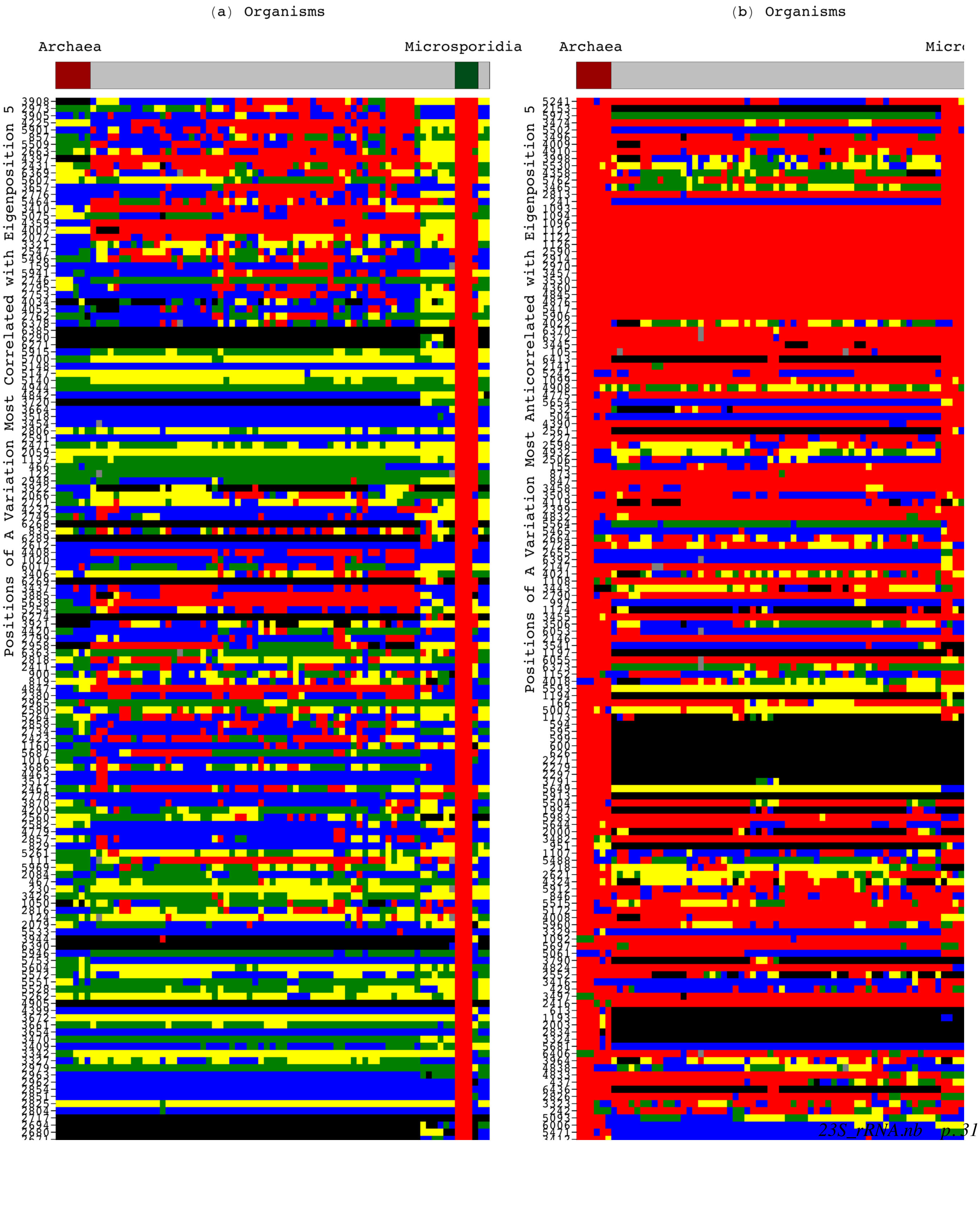

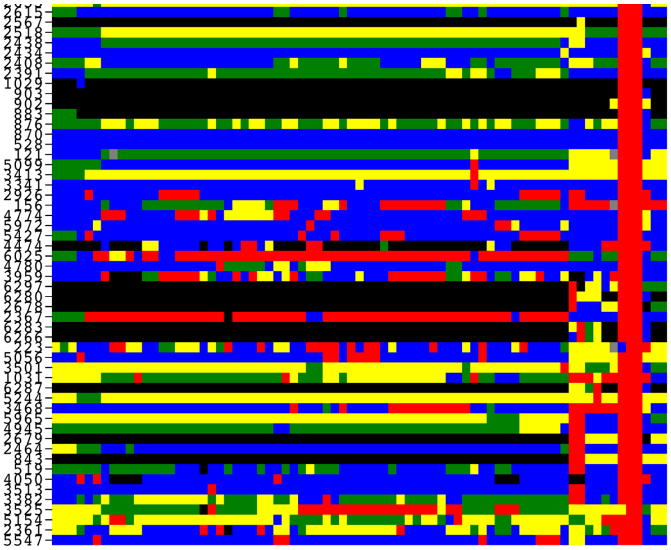

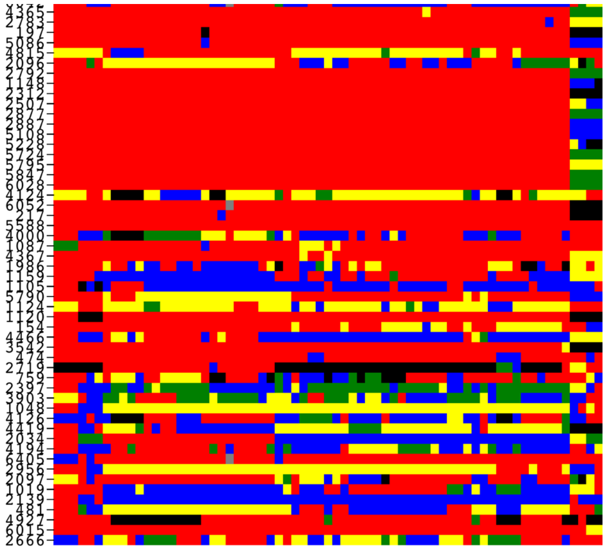

Supplement: Mathematica Notebook S4 — Mode-1 HOSVD of the 23S rRNA alignment. A PDF format file, readable by Adobe Acrobat Reader. (PDF) [file pone.0018768.s005.pdf]
